# Mortality from HIV‐associated meningitis in sub‐Saharan Africa: a systematic review and meta‐analysis

**DOI:** 10.1002/jia2.25416

**Published:** 2020-01-19

**Authors:** Mark W Tenforde, Alida M Gertz, David S Lawrence, Nicola K Wills, Brandon L Guthrie, Carey Farquhar, Joseph N Jarvis

**Affiliations:** ^1^ Division of Allergy and Infectious Diseases University of Washington School of Medicine Seattle WA USA; ^2^ Department of Epidemiology University of Washington School of Public Health Seattle WA USA; ^3^ Botswana Harvard AIDS Institute Partnership Gaborone Botswana; ^4^ Department of Clinical Research Faculty of Infectious and Tropical Diseases London School of Hygiene and Tropical Medicine London United Kingdom; ^5^ Welcome Centre for Infectious Diseases Research in Africa Infectious Disease and Molecular Medicine Unit University of Cape Town Cape Town South Africa; ^6^ Department of Global Health University of Washington Seattle WA USA

**Keywords:** cryptococcal meningitis, pneumococcal meningitis, TB meningitis, sub‐Saharan Africa, systematic review

## Abstract

**Introduction:**

HIV‐associated cryptococcal, TB and pneumococcal meningitis are the leading causes of adult meningitis in sub‐Saharan Africa (SSA). We performed a systematic review and meta‐analysis with the primary aim of estimating mortality from major causes of adult meningitis in routine care settings, and to contrast this with outcomes from clinical trial settings.

**Methods:**

We searched PubMed, EMBASE and the Cochrane Library for published clinical trials (defined as randomized‐controlled trials (RCTs) or investigator‐managed prospective cohorts) and observational studies that evaluated outcomes of adult meningitis in SSA from 1 January 1990 through 15 September 2019. We performed random effects modelling to estimate pooled mortality, both in clinical trial and routine care settings. Outcomes were stratified as short‐term (in‐hospital or two weeks), medium‐term (up to 10 weeks) and long‐term (up to six months).

**Results and discussion:**

Seventy‐nine studies met inclusion criteria. In routine care settings, pooled short‐term mortality from cryptococcal meningitis was 44% (95% confidence interval (95% CI):39% to 49%, 40 studies), which did not differ between amphotericin (either alone or with fluconazole) and fluconazole‐based induction regimens, and was twofold higher than pooled mortality in clinical trials using amphotericin based treatment (21% (95% CI:17% to 25%), 17 studies). Pooled short‐term mortality of TB meningitis was 46% (95% CI: 33% to 59%, 11 studies, all routine care). For pneumococcal meningitis, pooled short‐term mortality was 54% in routine care settings (95% CI:44% to 64%, nine studies), with similar mortality reported in two included randomized‐controlled trials. Few studies evaluated long‐term outcomes.

**Conclusions:**

Mortality rates from HIV‐associated meningitis in SSA are very high under routine care conditions. Better strategies are needed to reduce mortality from HIV‐associated meningitis in the region.

## 1 Introduction

Approximately 37 million people were living with HIV worldwide in 2017, with over two‐thirds in sub‐Saharan Africa (SSA) 1. Although access to combined antiretroviral therapy (ART) has improved, a large proportion of people living with HIV (PLHIV) still present with advanced immune suppression and are at high risk for HIV‐related infections 2, 3. Central nervous system (CNS) infections are a major cause of mortality in PLHIV in SSA, with cryptococcal, TB and pneumococcal meningitis the most common microbiologically confirmed aetiologies 4, 5. Cryptococcal meningitis alone results in approximately 15% of HIV‐associated deaths worldwide, with almost three‐quarters of these in SSA 6.

Treatment for HIV‐associated meningitis is challenging even in well‐resourced settings. Recommended cryptococcal meningitis management involves initial “induction” therapy with intravenous amphotericin (ideally for one week with flucytosine 7, which remains unavailable throughout most of Africa 8), an effective but highly toxic drug 9, followed by consolidation and maintenance therapy with fluconazole 10. However, less potent fluconazole monotherapy is often used in resource‐constrained settings due to low cost, ease of oral administration and better drug toxicity profile. Additionally, cryptococcal meningitis survival is associated with performance of therapeutic lumbar punctures (LPs) to reduce intracranial pressure (ICP) 11, 12, intravenous fluid (IVF) hydration and electrolyte supplementation and monitoring to prevent life‐threatening amphotericin‐related toxicities 9, 10, 13, and appropriate follow‐up after hospital discharge to initiate antiretroviral therapy (ART) around four to six weeks to reduce the risk of immune‐reconstitution inflammatory syndrome 14. Significant barriers exist to optimal management in most SSA settings, such as lack of more potent antifungal drugs, lack of manometers to measure ICP, hospital understaffing, limited laboratory services and loss to follow‐up after hospital discharge. Similarly, effective management of TB meningitis is complicated in resource‐constrained settings with challenges in management of common antituberculous drug toxicities, long duration of treatment (of a minimum of six months), poor sensitivity of diagnostic studies and delays for culture results, emergence of drug resistance and lack of integration of TB and HIV services 15, 16. Survival from pneumococcal meningitis can be negatively affected by delayed healthcare access and lack of timely initiation of effective antimicrobial therapy 17.

Several systematic reviews have evaluated outcomes of HIV‐associated meningitides in Africa, but each has important limitations. A recent review of long‐term outcomes from HIV‐associated cryptococcal meningitis included only a small subset of studies with long‐term outcomes data which may not be generally representative 18. In a previous systematic review of TB meningitis in Africa, investigators did not differentiate between short‐ and long‐term outcomes and combined outcomes from studies using heterogeneous case definitions (e.g. microbiologically diagnosed TB or TB diagnosed clinically using various criteria), limiting interpretation 19. A prior well‐conducted systematic review of pneumococcal meningitis in Africa focused on paediatric populations 20.

Given the large burden and mortality from HIV‐associated meningitis in SSA and limitations of prior studies, we performed a systematic review and meta‐analysis to evaluate short‐ and long‐term outcomes of common HIV‐associated meningitides (cryptococcal, TB and pneumococcal). We estimated mortality in “routine care” studies (which we defined as studies in which hospital care was provided primarily by the local medical team, for example, retrospective cohort studies), providing realistic global burden estimates important for resource allocation and prevention efforts such as cryptococcal antigen (CrAg) screening 21. We compared outcomes from routine care to “clinical trial” studies (which we defined as randomized‐controlled trials (RCTs) or prospective cohorts with regular day‐to‐day management of patients involving study investigators) to characterize the outcomes gap and provide insights into potential reductions in mortality that might be realized through improved management strategies.

## 2 Methods

### 2.1 Types of outcomes measures

Our overall aim was to determine the mortality associated with the most common HIV‐associated meningitides in sub‐Saharan Africa, captured at time points reflecting acute‐ and long‐term outcomes. Our mortality endpoints were defined as: (1) Short‐term (death ≤2 weeks after hospitalization or enrolment); (2) medium‐term (death ≤10 weeks); and long‐term (≤6 months). Study deviations from these pre‐defined timepoints were described in the analysis.

### 2.2 Types of studies and participants

We included published RCTs and observational studies (cohort studies, case‐control studies, cross‐sectional audits or surveillance studies) of adults (≥18‐years) treated for cryptococcal, TB and/or pneumococcal meningitis. Inclusion criteria included: (1) Study from sub‐Saharan Africa (or restricted to patients from sub‐Saharan Africa in multi‐centre studies with participants also enrolled from outside of the region 22); (2) ≥15 participants treated for cryptococcal, TB or pneumococcal meningitis; and 3) full period of observation after 1990 (to reflect emergence of HIV in SSA and modern antimicrobial therapy regimens). Exclusion criteria included: (1) Paediatric study or with a majority of patients <18‐years old without disaggregated results provided in adults; (2) case report, case series, or <15 participants with cryptococcal, TB or pneumococcal meningitis; (3) antimicrobial treatment not available or treatment with therapies either not used or contraindicated (e.g. acetazolamide for cryptococcal meningitis) 22, 23; (4) non‐representative or biased sampling (e.g. significant RCT recruitment delay causing immortal time bias 14, 24; (5) missing mortality data for >15% of participants or mortality outcomes not provided; (6) diagnostic criteria not clearly defined; (7) repeat data from another study; or 8) not a full article (e.g. conference abstract).

For cryptococcal meningitis, we included studies of cases confirmed by cerebrospinal fluid (CSF) testing (i.e. positive India ink, culture or CrAg), excluding those with a significant number (>10%) of patients diagnosed through peripheral blood CrAg testing alone, as blood CrAg testing is unable to definitively confirm central nervous system involvement. For TB meningitis, we included studies of either microbiologically confirmed cases or with diagnostic criteria that combined CSF findings and other supportive evidence, for example, isolation of *Mycobacterium tuberculosis* at another site or suggestive imaging 25. For pneumococcal meningitis, we included studies with mortality for microbiologically confirmed cases or, when results were not disaggregated with other cases, if a majority of cases (>50%) were microbiologically confirmed. These definitions were used so that outcomes would reflect mortality from the aetiologies of interest rather than other potential causes.

### 2.3 Search method and data collection

**Figure 1:**
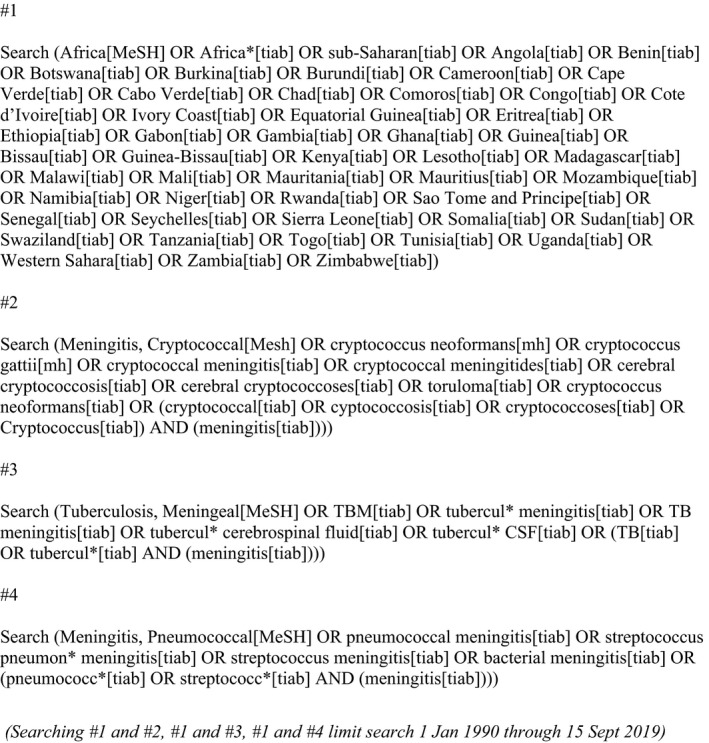
PubMed search strategy.

We searched for studies from 1 January 1990 through 30 April 2018 using PubMed, EMBASE, and the Cochrane Central Register of Controlled Trials (CENTRAL). The search was updated through 31 December 2018 in PubMed and EMBASE with a subsequent update through 15 September 2019 in PubMed. Our search strategy combined geographic and cryptococcal, TB and pneumococcal meningitis search terms (Figure 1 and Appendix S1). We excluded conference abstracts and studies that were not peer‐reviewed. No restrictions were placed on age or language. The search strategy and protocol was developed by the authors before the search; the study protocol was not published.

Articles obtained in database searches were aggregated and de‐duplicated in Covidence 26. Two reviewers (MWT and AMG) independently performed a primary title and abstract search to identify potentially eligible studies. The authors then reviewed full text articles to assess for inclusion, with data from included studies abstracted using standardized data collection forms. Secondary review was provided by DSL and NW. Discrepancies were decided through consensus or adjudication from JNJ if needed. We contacted authors as needed for clarification. Reference lists of included articles as well as relevant reviews were searched to identify other potential studies. We generated a flow diagram, following PRISMA recommendations, and summary tables of excluded full‐text articles with rationale (in Appendix S1), and reporting was conducted per PRISMA recommendations 27.

### 2.4 Data synthesis and analysis

Data were pooled using standard random effects meta‐analyses for proportions, with variance of proportions stabilized using Freeman‐Tukey double arcsine transformation before pooling 28. We estimated the pooled proportion of deaths and 95% confidence interval for each pathogen (*Cryptococcus*, TB and pneumococcus) by study type (clinical trial vs routine care study) and by timepoint (short‐term, medium‐term and long‐term mortality). Patient outcomes data were combined for multiple routine care studies from the same facility if the studies included patients from non‐overlapping periods. Heterogeneity was assessed with I^2^ values, representing the percentage of total variability due to between‐study heterogeneity. Results were presented using forest plots, along with summary tables of study and patient characteristics (included in Appendix S1). Analyses were conducted in R Studio using the *metaprop* command 29.

### 2.5 Sub‐group analyses

We performed additional analyses to stratify estimates by clinically important confounders. For cryptococcal meningitis, we stratified outcomes by induction therapy; amphotericin B with and without flucytosine (for <2 or ≥2 weeks), fluconazole with flucytosine, fluconazole or treatment not specified. Outcomes from induction regimens with amphotericin B alone or amphotericin B with fluconazole were combined, as no mortality difference has been documented between groups 30, 31. For TB meningitis, we stratified analyses by studies with microbiological‐confirmed cases and with a majority of cases defined using combined microbiological and clinical criteria without *Mycobacterium tuberculosis* confirmed from CSF culture, smear or PCR. For pneumococcal meningitis, we stratified analyses by treatment regimen. We further stratified outcomes by region (Southern, Eastern, Middle (Central) and Western Africa) as defined by the United Nations geoscheme 32, and studies conducted after 2000 or before 2000. We initially planned additional stratified analyses by HIV prevalence (≥50% or <50%) within a population, but most studies included a majority or only HIV‐positive patients and we therefore did not perform sub‐group analyses based on HIV status.

### 2.6 Dealing with missing data and assessing risk of bias

We described missingness of data, including clinical characteristics (e.g. HIV status) and mortality. We performed intention‐to‐treat analysis when multiple groups were compared. Outcomes were reported for patients with known outcomes at each mortality timepoint. This was done so as not to systematically bias estimates toward higher survival as would have done if those lost to follow‐up were all assumed to have survived. If loss to follow‐up exceeded 15%, we excluded the study from the analysis at that timepoint due to the high likelihood that outcomes were not missing at random (i.e. those who died were more likely to be lost to follow‐up). Given the heterogeneity of study types, we undertook a subjective assessment of study quality based on previous guidance for systematic reviews 33. This incorporated quality of reporting of important covariates, for example, timing of ART in ART‐naïve patients treated for cryptococcal meningitis. Randomized‐controlled trials of HIV‐associated cryptococcal meningitis have previously been assessed for risk of bias using GRADE criteria 30.

## 3 Results and discussion

### 3.1 Overall search findings

**Figure 2:**
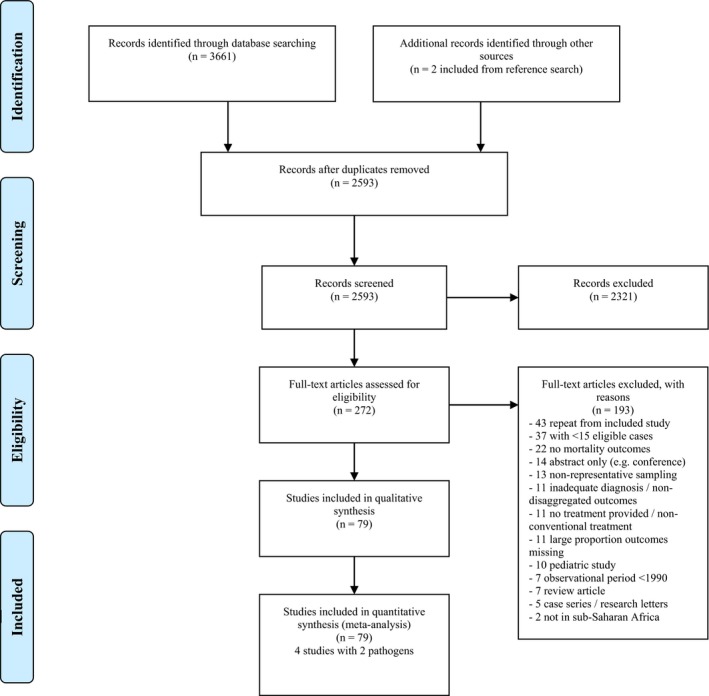
PRISMA diagram.

The initial database search yielded 3661 titles, with two additional articles found that met inclusion criteria. After removing duplicates, 2593 titles and abstracts were reviewed, with 272 selected for full text review. Of these, 193 were excluded (Figure 2 and Appendix S1). Seventy‐nine studies were included from the primary database search and additional references.

### 3.2 Cryptococcal meningitis outcomes from treatment provided under “routine care” settings

Forty‐one observational studies with 10,139 cases of cryptococcal meningitis were included, most in HIV‐positive patients treated at district or referral hospitals (Appendix S1). The majority of studies were judged of low quality, with limited description of clinical management (e.g. performance of therapeutic lumbar punctures, antifungal therapy dose/duration, timing of ART following initiation of antifungal therapy in ART‐naïve patients). Seventeen were prospective cohorts and one was a mixed prospective and retrospective cohort 34, which we classified as routine care studies. Three of these prospective cohorts described more active involvement from a study team [35, 36, 37], but at irregular intervals with day‐to‐day management under local conditions. In 10% (4/41) of included studies, the period of observation began prior to 2000. Seventeen countries were represented, with greatest representation from South Africa (14 studies) 34, 37, 38, 39, 40, 41, 42, 43, 44, 45, 46, 47, 48, 49, Ethiopia (three studies) 50, 51, 52 and Uganda (three studies) 53, 54, 55; other countries had two or fewer studies 11, 35, 36, 56, 57, 58, 59, 60, 61, 62, 63, 64, 65, 66, 67, 68, 69, 70, 71, 72, 73, 74. Amphotericin B‐based induction therapy (with or without fluconazole) was the predominant induction regimen in 16 studies, fluconazole in 13 studies, and a mix of treatments (45% fluconazole and 43% amphotericin B) in one study 44, with antifungal regimens not specified in 11 studies. In 28 studies with details on ART status, 26% (1792/6777) had a history of current or previous ART use. Reasons for exclusion are detailed in Appendix S1; these included non‐representative sampling 24, 75, 76, 77, high loss to follow‐up or duration of follow‐up not clear 62, 78, antifungal treatment not available 23, 79, 80 and use of treatment regimens not used or contraindicated in management 81, 82, 83.

**Figure 3:**
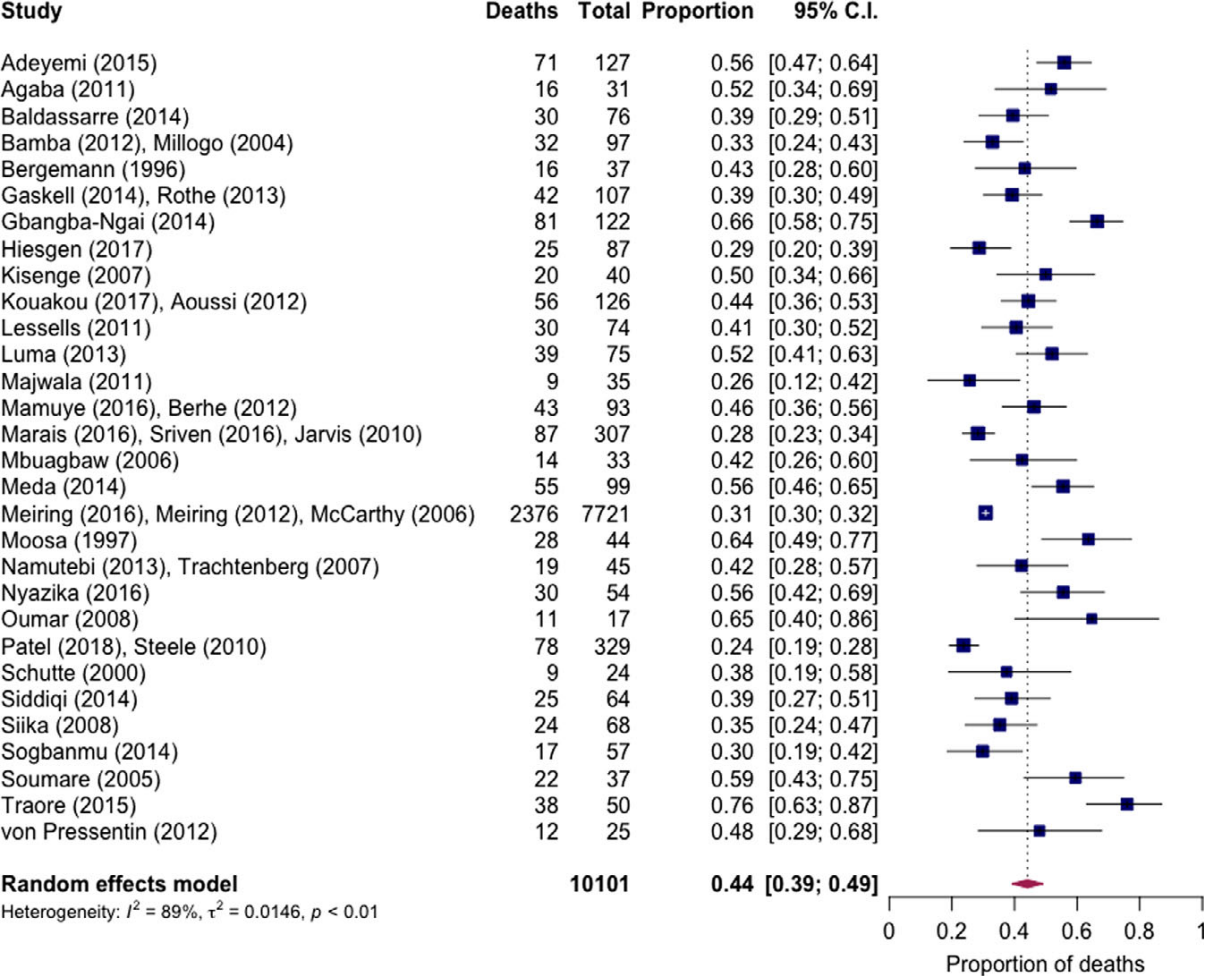
Overall pooled short‐term mortality of cryptococcal meningitis in routine care settings.

Short‐term mortality was reported in 40/41 included studies (35 in‐hospital and five studies with two‐week mortality reported). Pooled short‐term mortality was 44% (95% CI: 39% to 49%, 40 studies), with high between‐study heterogeneity (I^2^ = 89%) (Figure 3). Mortality was lowest in Southern Africa (37% (95% CI: 31% to 43%), I^2^ = 86%, 16 studies) and highest in Central Africa (55% (95% CI: 41% to 69%), I^2^ = 75%, three studies) and West Africa (54% (95% CI: 40% to 67%), I^2^ = 83%, eight studies) (Figure S1). Restricting to studies with a full observation period after 2000, pooled mortality was 44% (95% CI: 39% to 49%, I^2^ =  90%, 36 studies) (similar in a post‐hoc analysis restricted to studies with a full period of observation after 2005, with a pooled mortality of 42% (95% CI: 36% to 47%, I^2^ = 89%, 29 studies)). For these studies, short‐term mortality was 41% (95% CI: 32% to 51%, I^2^ = 91%, 15 studies) with exclusive or predominant amphotericin B‐based induction therapy (none using flucytosine), 41% (95% CI: 31% to 50%, I^2^ = 89%, 11 studies) with exclusive or predominant fluconazole, and 50% (95% CI: 39% to 61%, I^2^ = 78%, nine studies) with treatment not specified. One study was excluded in which the proportion of patients receiving amphotericin B‐based and fluconazole induction therapy was almost equivalent 44.

Few studies reported medium‐ or long‐term mortality. Medium‐term mortality (defined as death ≤10‐weeks, except ≤3‐months in one study) was described in five studies with 422 patients in Southern Africa (two studies) or East Africa (three studies), with a pooled mortality of 51% (95% CI: 43% to 60%, I^2^ = 60%) (Figure S2). Long‐term mortality was described in three studies (at nine months in one and 12 months in two 35, 42, 56) with 294 patients in Southern Africa (two studies) and East Africa (one study). Pooled mortality was 63% (95% CI: 46% to 78%, I^2^ = 79%) (Figure S3). Studies from Botswana 56, with standard amphotericin B and fluconazole induction therapy, and Malawi 35, with high‐dose fluconazole (800 mg/day) induction therapy, reported one‐year mortality of 65% (142/219) and 77% (43/56) respectively among those not lost to follow‐up. The third study, a prospective cohort from South Africa, reported a nine‐month mortality of 37% (7/19) 42. Few participants in this study had baseline altered mental status (11% (2/19) noted to have confusion), an established predictor of mortality 84, and investigators may have recruited less seriously ill patients. Regions in SSA with the highest acute mortality (Central and Western Africa) did not report long‐term mortality data.

### 3.3 Cryptococcal meningitis outcomes for treatment provided under clinical trial settings

We included 18 studies from clinical trials (randomized trials or investigator‐managed cohorts that compared ≥1 treatment regimen for cryptococcal meningitis) with a total of 2048 cases, almost all HIV positive. These included 10 RCTs and eight prospective cohorts, most at referral centres and all conducted after 2000 (Appendix S1). The quality was judged to be good for most studies (details on therapeutic lumbar puncture details in 12/18, timing of ART initiation in 17/18 and description of antifungal regimen in all studies), and most RCTs had been previously evaluated using GRADE criteria 30. Nine countries were represented (including several multi‐country RCTs); greatest representation was from South Africa (six studies) 85, 86, 87, 88, 89, 90, Uganda (six studies) 22, 91, 92, 93, 94, 95 and Malawi (four studies) 7, 22, 96, 97, with other countries represented in two or fewer studies 98, 99, 100. Ten of the eighteen studies excluded patients on ART at the time of enrolment. Two studies described fluconazole monotherapy as the only regimen and a third RCT evaluated fluconazole as one treatment arm 93, 97, 100, two RCTs evaluated combined flucytosine and fluconazole therapy 7, 97, and the rest described use of amphotericin B with or without flucytosine or fluconazole. Reasons for study exclusion are detailed in Appendix S1: These included use of abnormal treatment regimens 101, 102, 103, 104, studies with non‐representative sampling, for example, delayed enrolment for one week after initiation of antifungal therapy 14, and a study that reported longer‐term outcomes from only a subset of patients from an included study 105. For RCTs that compared standard regimens to experimental regimens not part of clinical practice, for example, adjunctive interferon‐gamma or sertraline 87, 106, or therapies demonstrated to be harmful, for example, adjunctive dexamethasone 22, we restricted our analysis to patients who received standard antifungal regimens. For one RCT that recruited patients both in African and Asian health centres, we restricted our analysis to patients from Africa 22.

**Figure 4:**
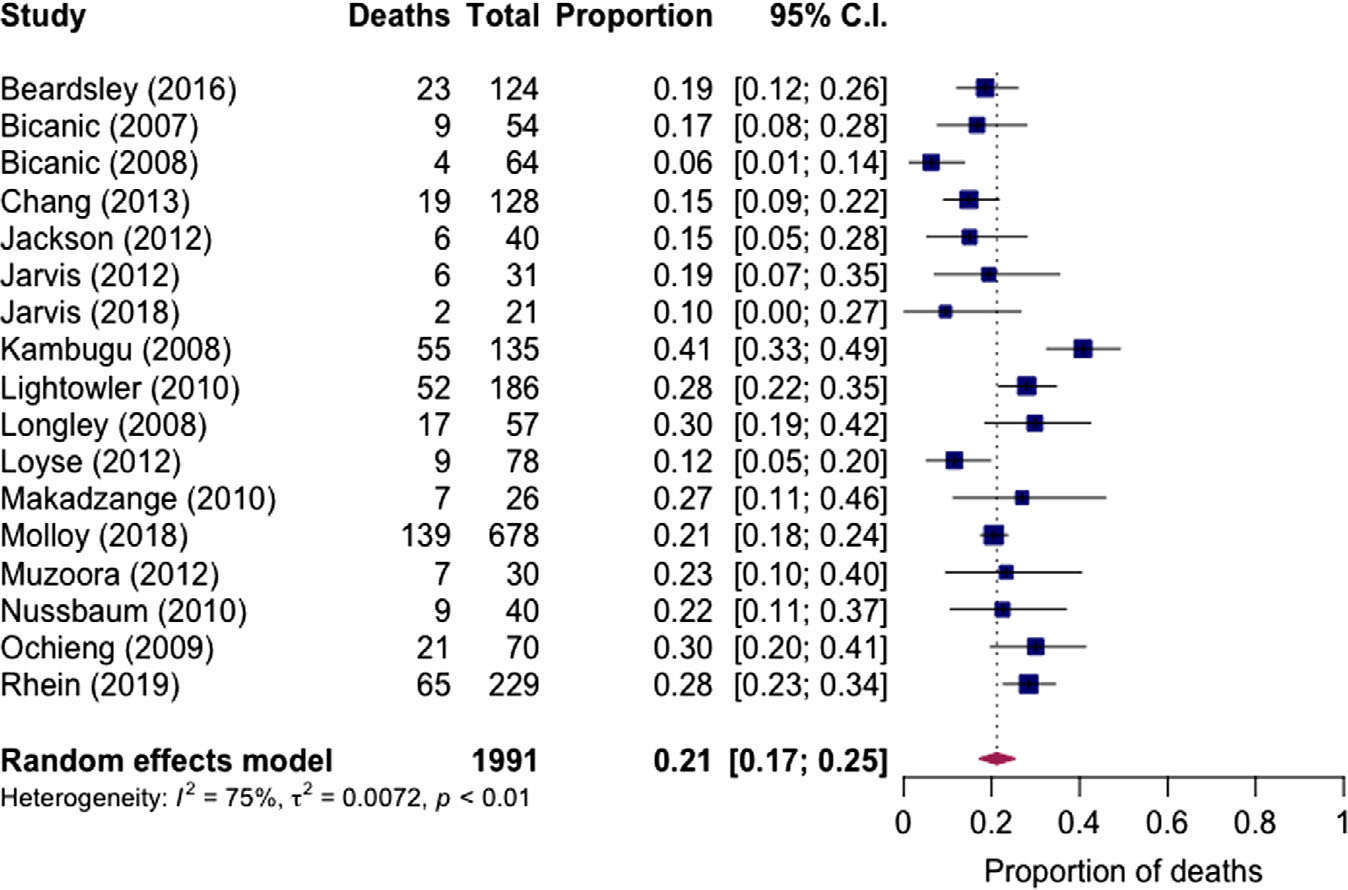
Pooled short‐term mortality of cryptococcal meningitis in clinical trial settings.

Short‐term mortality was reported in 17 studies (all reporting two‐week outcome) with 1991 cases. Pooled two‐week mortality was 21% (95% CI: 17% to 25%, I^2^ = 75%, 17 studies) (Figure 4). Stratified by treatment regimen, mortality was 12% (95% CI: 7% to 18%, I^2^ = 40%, 5 studies) for amphotericin and flucytosine induction therapy, 17% (95% CI: 12% to 22%, I^2^ = 0%, two studies) for flucytosine with fluconazole, 23% (95% CI: 19% to 28%, I^2^ = 72%, 12 studies) for amphotericin with or without fluconazole and 30% (95% CI: 21% to 40%, I^2^ = 0%, three studies) for fluconazole alone. Little mortality difference was observed for shorter (<2 weeks) and longer (two‐week) amphotericin‐based induction regimens.

Medium‐term mortality (at 10 weeks except four months in one study 92) was reported in 13 studies with 1487 cases, with a pooled mortality of 37% (95% CI: 33% to 41%, I^2^ = 42%) (Figure S4). Stratified by induction regimen, mortality was 30% (95% CI: 25% to 34%, I^2^ = 0%, five studies) for amphotericin with flucytosine, 35% (95% CI: 29% to 42%, I^2^ = 0%, two studies) for flucytosine with fluconazole, 39% (95% CI: 35% to 43%, I^2^ = 14%, eight studies) for amphotericin with or without fluconazole and 49% (95% CI: 39% to 60%, I^2^ = 29%, three studies) for fluconazole. Long‐term mortality (within six months except 18 weeks in one) was described in six studies with 614 cases, with a pooled mortality of 44% (95% CI: 36% to 52%, I^2^ = 71%) (Figure S5). Stratified by regimen, mortality was 33% (95% CI: 21% to 45%, one study) for amphotericin with flucytosine, 46% (95% CI: 36% to 55%, I^2^ = 78%, four studies) for amphotericin with or without fluconazole and 50% (95% CI: 30% to 70%, one study) in a small study from Zimbabwe of fluconazole monotherapy.

### 3.4 TB meningitis outcomes

Twelve studies with 1008 TB meningitis cases were included, all routine care studies and all but one with a majority or all HIV positive (median 84%) 107. One recent study in Zambia, with 19% culture‐confirmed adults TB meningitis cases and a 86% HIV prevalence, contributed to over half of these cases 108. Study quality was relatively low, with most studies not including details of ART or antituberculous treatment or average duration of hospitalization. Five included cases before 2000 34, 109, 110, 111, 112, whereas seven had a full period of observation after 2000 53, 60, 67, 107, 108, 113, 114. Eight countries were represented, with greatest representation from South Africa (three studies), Uganda (two studies) and Zambia (two studies). Six studies included exclusively definite cases (with positive CSF AFB smear, culture or PCR) and six included mostly probable/possible cases (range 0% to 21% microbiologically confirmed, with two studies using Marais criteria 25, 108). One study specified use of adjunctive dexamethasone 111. Several potential studies were excluded on full‐text review (Appendix S1): Reasons included TB case series, for example, exclusively multi‐drug resistant cases 115, 116, 117, high risk of selection bias 42, 118, 119, 120, significant missingness of outcomes 121, 122, 123 and unknown or inadequate description of diagnostic criteria 50, 74, 124, 125, 126.

**Figure 5:**
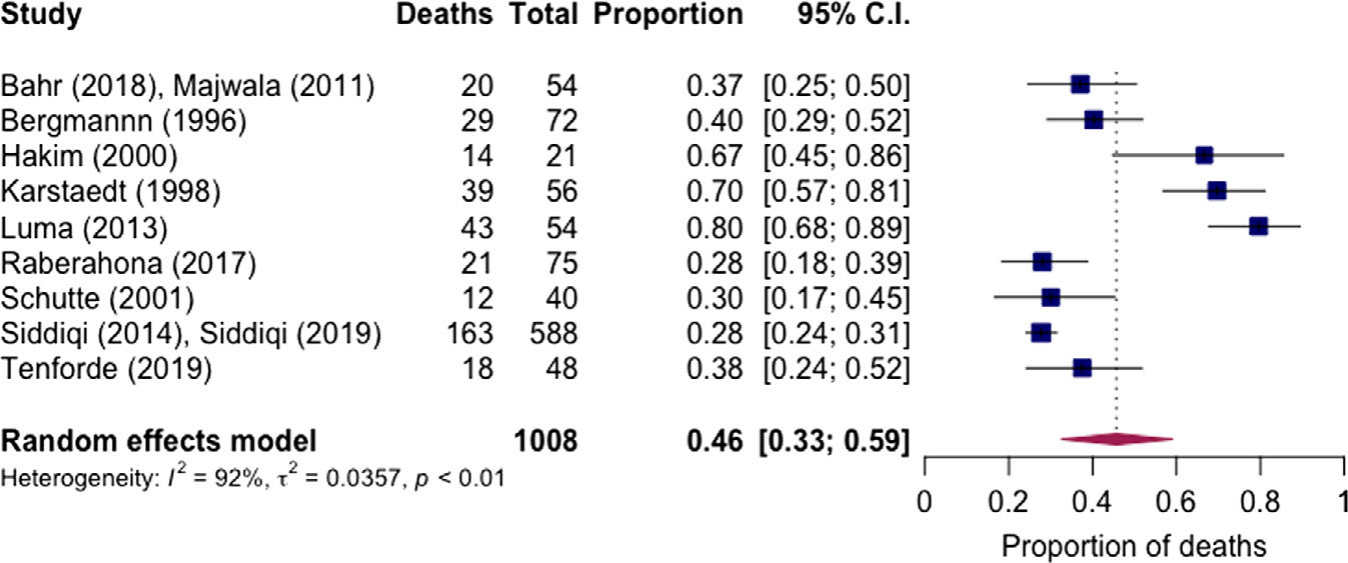
Pooled short‐term mortality of TB meningitis in routine care settings.

Short‐term mortality for 1008 cases was reported in 11 studies (two from a single hospital with combined patient outcomes in Uganda 53, 113 and two from a single hospital with combined outcomes in Zambia 67, 108; two‐week mortality specified in one study 114, in‐hospital mortality in ten studies with length of hospitalization not specified). Pooled mortality was 46% (95% CI: 33% to 59%, I^2^ = 92%, 11 studies) (Figure 5), and was higher in studies with only definite cases (51% (95% CI: 37% to 65%), I^2^ = 78%, six studies) and mostly possible/probable cases (40% (95% CI: 23% to 59%), five studies). Restricting to studies after 2000, pooled short‐term mortality was 42% (95% CI: 25% to 59%, I^2^ = 93%, seven studies). Ten‐week mortality from a national meningitis audit in Botswana of microbiologically confirmed cases was 46% (95% CI: 31% to 61%) 114. Long‐term mortality was reported in two studies, within one year in the study from Botswana and after completion of six months of antituberculous therapy in a Nigerian study 111, both studies with exclusively definite cases. Reported mortality for these studies was 56% (27/48) in Botswana and 78% (31/40) in Nigeria. Of note, the Botswana study relied on national electronic death registry data, which has been previously shown to accurately capture deaths but might slightly under‐estimate mortality 56. Long‐term outcomes in the large study were excluded because of high degree of data missingness (>20% at one year) 108. These investigators reported a one‐year mortality of 59% among patients who could be tracked, or 67% assuming those lost to follow‐up had died.

### 3.5 Pneumococcal meningitis outcomes

Twelve studies with 3935 cases of pneumococcal meningitis were included, a majority HIV‐positive in nine studies, <50% HIV positive in one, and with HIV prevalence not specified in two studies. Nine were classified as routine care studies 114, 127, 128, 129, 130, 131, 132, 133, 134 and two were RCTs 135, 136. One study consisted of two sequential cohorts, a lead‐in observational routine care cohort followed by a prospective cohort receiving a bundled Goal Directed Therapy (GDT) intervention that included parenteral ceftriaxone therapy within one hours of registration, airway support, and fluid resuscitation according to sepsis guidelines, among other supportive measures 137. The clinical trial studies were judged to be of high quality, but routine care studies of relatively low quality (including lack of description of ART status and dose and duration of antibiotic therapy). Eight countries were represented, with four studies (two RCTs, one observational cohort and the combined sequential cohort study) from a single referral hospital in Malawi. Nine studies included a full period of observation after 2000. Ten provided outcomes for microbiologically confirmed cases (CSF culture, PCR, and/or antigen testing) and two studies with *Streptococcus pneumoniae* microbiologically confirmed for a majority of cases (64% to 69%). Treatment varied significantly between studies; four described ceftriaxone as standard antibiotic therapy (one RCT with adjunctive dexamethasone used in a single treatment arm) 114, 135, two included chloramphenicol‐based treatment as the predominant treatment (one combined with intravenous penicillin G) 127, 129, two included aminopenicillins (amoxicillin or ampicillin) with or without gentamycin as the predominant therapy given 130, 132, three studies did not specify therapy 128, 131, 134, and one study included a similar mix of treatment with ceftriaxone and chloramphenicol 133. Several potential studies were excluded on full text review (Appendix S1): Reasons included a minority of cases with *Streptococcus pneumoniae* isolation 34, 138, 139, 140 and high loss to follow‐up / missing outcomes data 141, 142, 143, 144, 145.

**Figure 6:**
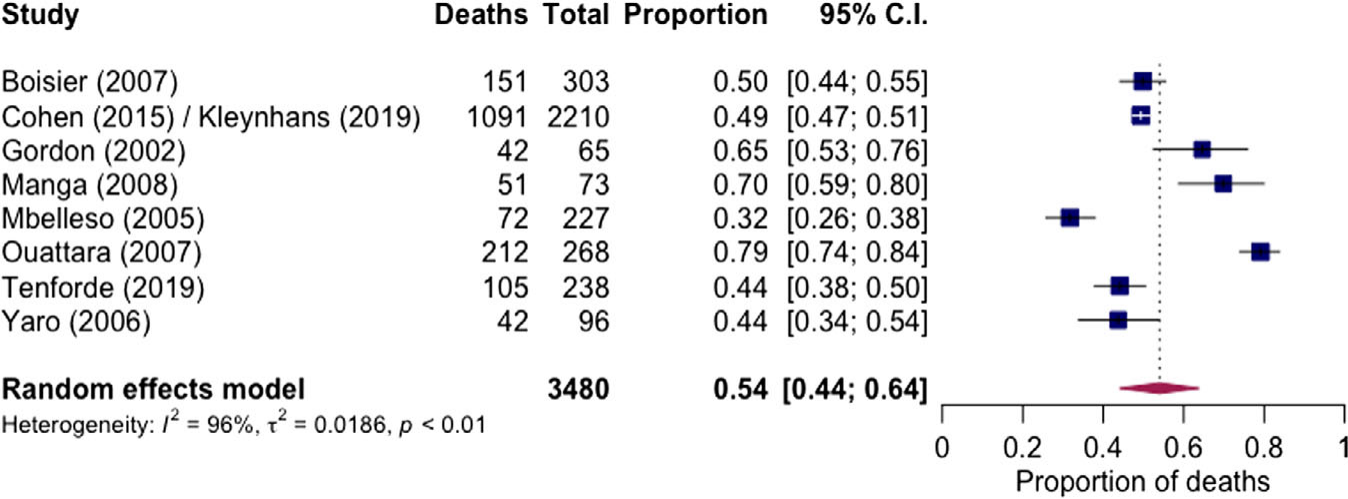
Pooled short‐term mortality of pneumococcal meningitis in routine care settings.

Short‐term mortality was reported in all nine routine care studies with 3480 cases (including 14‐day 114, 30‐day 128, in‐hospital 129, 130, 131, 132, 134 and with length of follow‐up not specified in two surveillance studies but assumed short‐term 127, 133). Over half of these cases (64%, 2210/3480) were from two surveillance studies in Gauteng Province, South Africa 128, 134; the first from 2003 to 2008 reporting mortality up to 30 days and the second with data from 2013 to 2016 with in‐hospital mortality reported, with results pooled together for the meta‐analysis. Pooled mortality was 54% (95% CI: 44% to 64%, I^2^ = 96%, nine studies) (Figure 6). Medium‐ and long‐term mortality was described in two routine care studies, one from Botswana and the other from Malawi 114, 129. Both found generally favourable long‐term survival in those with short‐term survival. The study from Malawi showed a short‐term (in‐hospital up to nine days), medium‐term (10‐week) and long‐term (six‐month) mortality of 65% (42/65), 69% (45/65) and 69% (45/65) respectively, with one‐year mortality of 75% (49/65). The study from Botswana found short‐term (two‐week), medium‐term (10‐weeks), long‐term (six‐month) and one‐year mortality of 44% (105/238), 47% (112/238), 47% (113/238) and 49% (117/238) respectively.

The two RCTs were both conducted at the same referral hospital in Malawi, provided 40‐day outcomes, and reported treatment with ceftriaxone (with or without dexamethasone) 135, 136. We combined data for patients treated with or without adjunctive dexamethasone in one RCT as no difference in mortality was found between arms 136, and patients receiving adjunctive glycerol in one RCT were excluded as glycerol did not improve outcomes and may have been associated with severe adverse events 135. Short‐term (here defined as 40‐day) mortality ranged from 39% (20/51) to 51% (140/272); however, mortality from pneumococcal meningitis was certainly under‐estimated in the smaller trial as 17% of patients with suspected acute bacterial meningitis died before they could be recruited. In the RCT with a 51% (140/272) 40‐day mortality, long‐term mortality was reported at 61% (150/245) in patients not lost to follow‐up.

The study with the combined sequential observational routine care cohort and interventional Goal Directed Therapy cohort was conducted at the same centre as the two RCTs, with ceftriaxone treatment provided as standard treatment 137. At the primary endpoint of 40 days, among patients with known outcomes 49% (28/57) had died in the lead‐in observational cohort and 63% (38/60) in the subsequent clinical trial cohort.

## 4 Discussion

This systematic review and meta‐analysis provides comprehensive estimates of outcomes from common HIV‐associated meningitides in sub‐Saharan Africa and addresses important limitations of previous systematic reviews, including disaggregation of mortality from clinical trial (prone to selection of non‐representative patients as well as more intensive management) and routine care settings, and evaluation of outcomes at different timepoints. Pooled mortality in routine care settings was high, with approximately half of patients diagnosed with cryptococcal, TB and pneumococcal meningitis dying within two weeks or during hospitalization. Our results highlight an overall poor understanding of long‐term outcomes in routine care settings given the lack of published outcomes. Furthermore, most studies reported outcomes for patients treated at referral centres, where staffing, pharmaceutical and laboratory resources are likely better than in lower‐level health facilities. Our findings also suggest significant heterogeneity in mortality overall and between regions: Pooled short‐term mortality for cryptococcal meningitis in routine care settings within Central and West Africa exceeded 50%, compared to 37% in Southern Africa. The large heterogeneity in outcome could be attributable to differences in resources between regions and individual hospital facilities, intensity of care received and time from symptoms onset to presentation to care for patients, among other factors.

Comparing mortality of patients treated under routine care conditions to clinical trial settings, we observed significant differences for cryptococcal meningitis but similar mortality for pneumococcal meningitis. Short‐term mortality for cryptococcal meningitis managed under routine care conditions was twofold higher compared to mortality in clinical trial settings (44% vs. 21%); which remained similar when stratified by treatment regimen (e.g. 41% vs. 23% for amphotericin B without flucytosine). This difference reflects a degree of differences in patients enrolled in clinical trials compared to routine care studies (e.g. exclusion of more severely ill patients, such as those with severe metabolic abnormalities or unable to provide consent, in randomized‐controlled trials). However, this likely also reflects benefits from therapeutic lumbar punctures, standardized intravenous fluid and electrolyte supplementation, and better management of intravenous catheters to prevent thrombophlebitis and catheter‐associated bacteraemia in clinical trials 146. In contrast, short‐term mortality from pneumococcal meningitis differed little between RCTs and routine care settings, perhaps reflecting less intensive management currently recommended for pneumococcal disease and highlighting a need for better management strategies. In routine care settings, more than two‐thirds of patients died during the course of treatment for TB meningitis from two studies, although data were not available to compare these findings with clinical trials. This contrasts with 39% (68/174) nine‐month mortality in HIV‐infected patients with TB meningitis receiving standard treatment in a recent RCT from Vietnam 147. One prospective cohort from South Africa with investigator management reported a 12% (3/34) nine‐month mortality of patients treated for HIV‐associated TB meningitis 42; but this study excluded patients with severe TB and those who did not initiate ART or were judged to have poor drug adherence.

Our findings suggest that the previously estimated 70% one‐year mortality for HIV‐associated cryptococcal meningitis in sub‐Saharan Africa may be conservative in some regions 6, particularly Central and West Africa where pooled short‐term mortality exceeded 50% and long‐term outcomes were not reported. Our findings also suggest that improved availability of more fungicidal – but more toxic – amphotericin B therapy alone may not have a large impact in reducing mortality. Under routine care settings, short‐term mortality for amphotericin‐based and fluconazole‐based regimens was similar at around 40% and long‐term mortality was reported at 65% even with amphotericin B and high‐dose fluconazole in Botswana 56. Recent evidence from a large RCT suggests that, combined with flucytosine (5FC), a shortened one‐week course of amphotericin B is the most effective antifungal regimen to reduce mortality from HIV‐associated cryptococcal meningitis 7, with similar rates of early fungal clearance compared to longer two‐week courses and less drug toxicity 30. Of a subset of participants from Malawi in this trial 105, mortality remained relatively low at 28% (11/40) at one year in the group treated with a one‐week course of amphotericin B combined with flucytosine. Thus, alongside expanding access to amphotericin B formulations, there is an urgent need for flucytosine access in sub‐Saharan Africa as well as future outcomes data evaluating this regimen in usual care settings. Furthermore, standardized intravenous fluid and electrolyte supplementation and therapeutic lumbar puncture schedules are associated with improved outcomes in HIV‐associated cryptococcal meningitis and care checklists and protocols could be operationalized even in many resource‐limited settings to reduce mortality 11, 13.

Our study has a number of important limitations. First, we applied strict diagnostic criteria. This resulted in the exclusion of a number of TB meningitis studies in setting where microbiological tests (e.g. culture or PCR) were not available or a majority of cases were diagnosed based on clinical suspicion without clear diagnostic standards. This may have over‐sampled cases from clinical settings with better diagnostic capability (e.g. TB PCR) or cases of TB meningitis with a higher CSF bacillary burden. We found higher mortality in studies with only microbiologically confirmed cases than in studies with most cases diagnosed using combined laboratory, clinical, and/or imaging features. Second, in comparing outcomes for cryptococcal meningitis between routine care and clinical trial settings, we were unable to determine the proportion of excess mortality in routine care settings attributable to different factors (e.g. lack of therapeutic lumbar punctures vs. poor laboratory monitoring vs. poor completion of therapy). In reality, the high mortality in routine care settings is likely multi‐factorial and our findings highlight the need for better management strategies such as protocolized care, less toxic short‐course therapy, and better preventive strategies including CrAg screening for individuals with advanced HIV/AIDS 21. Third, the classification of some prospective cohort studies as clinical trials vs. routine care studies required some judgment by the author team. Furthermore, patient enrolment in prospective cohorts considered “routine care” studies may have led to better clinical management from local clinicians as well as some selection bias. Finally, duration of follow‐up varied between studies evaluating short‐term mortality, and observational studies of cryptococcal meningitis typically reported in‐hospital rather than two‐week mortality. This may have biased results slightly toward a higher acute mortality in routine care settings if length of stay was longer on average than two weeks; however, the average length of hospitalization reported from most studies was about two weeks.

## 5 Conclusions

In conclusion, we found very high mortality from common HIV‐associated meningitides in sub‐Saharan Africa, with pooled short‐term mortality for pneumococcal meningitis around 50% and long‐term mortality for TB and cryptococcal meningitis well in excess of 50% but with an overall lack of long‐term outcomes data. Importantly, we found a significant degree of heterogeneity in outcomes between different regions of SSA, likely reflecting both relative resource‐availability and other differences in management. Improved management strategies are needed and prevention efforts should be prioritized, including CrAg screening for prevention of cryptococcal meningitis and childhood pneumococcal vaccination to provide some population herd immunity against invasive pneumococcal disease 148.

## Competing interest

The authors report no relevant conflicts of interest.

## Authors’ contributions

MWT, BLG, CF and JNJ designed the research study. MWT, AMG, DSL, NKW and JNJ performed the research. MWT analysed the data and wrote the primary draft of the manuscript. All authors contributed to subsequent drafts and approved the final version of the manuscript.

## Supporting information


**Appendix S1.** Details of included and excluded studies and additional analyses.
**Figure S1.** Pooled short‐term mortality of cryptococcal meningitis in routine care settings, by region.
**Figure S2.** Pooled medium‐term mortality of cryptococcal meningitis in routine care settings, by region.
**Figure S3.** Pooled long‐term mortality of cryptococcal meningitis in routine care settings, by region.
**Figure S4.** Pooled medium‐term mortality of cryptococcal meningitis in clinical trial settings.
**Figure S5.** Pooled long‐term mortality of cryptococcal meningitis in clinical trial settings.
