# Supplementary material for: Mortality from HIV‐associated meningitis in sub‐Saharan Africa: a systematic review and meta‐analysis
Source: J Int AIDS Soc. 2020 Jan 19;23(1):e25416. doi: 10.1002/jia2.25416 (PMC6970088; doi:10.1002/jia2.25416)
Supplement: Supplementary file 1 — Appendix S1. Details of included and excluded studies and additional analyses. Figure S1. Pooled short‐term mortality of cryptococcal meningitis in routine care settings, by region. Figure S2. Pooled medium‐term mortality of cryptococcal meningitis in routine care settings, by region. Figure S3. Pooled long‐term mortality of cryptococcal meningitis in routine care settings, by region. Figure S4. Pooled medium‐term mortality of cryptococcal meningitis in clinical trial settings. Figure S5. Pooled long‐term mortality of cryptococcal meningitis in clinical trial settings. [file JIA2-23-e25416-s001.docx]

**Supplementary Appendix**

**Routine care cryptococcal meningitis studies - details:**

| First author [ref] | Year(s) | Study type | Country | Setting | Patients no. / details | Sex | Age (years) | HIV +ve | CD4 count (cells/µL) | Baseline ART status | Altered mental status | Diagnostic details | Treatment(s) | ART post-dx | Therapeutic LP management | Follow-up duration | Notes |
| --- | --- | --- | --- | --- | --- | --- | --- | --- | --- | --- | --- | --- | --- | --- | --- | --- | --- |
| Adeyemi 2015 | 2011-2012 | retrospective cohort | South Africa | urban district hospital (KwaZulu Natal) | 127 / >13 years of age diagnosed with cryptococcal meningitis | 51% (65/127) male | mean 36 (SD 9.8) | 119/127 (94%) known HIV-infected (others declined testing or died before diagnosis) | median 45 (IQR 21-115) | 43/96 (45%) with known HIV on ART on admission | NS | positive CSF India ink, CrAg, and/or culture | manuscript notes national guidelines included induction therapy with AmB followed by FLU consolidation | NS | 2% (2/127) with baseline opening pressure measured; therapeutic lumbar puncture details NS | 2 weeks | 20% (25/127) previously treated for cryptococcal meningitis; 68% (17/25) of these had defaulted FLU maintenance |
| Agaba 2011 | 2007 | retrospective cohort | Nigeria | tertiary hospital (Jos University Teaching) | 31 / HIV-infected adults (≥18 years) admitted to hospital | NS for cryptococcal meningitis; 31% (109/354) male of overall cohort | NS for cryptococcal meningitis; 35 (SD 9) for overall cohort | 100% (31/31) | NS | NS | NS | positive CSF India ink and/or culture | NS | NS | NS | In-hospital |  |
| Aoussi 2012 | 2005-2008 | retrospective cohort | Ivory Coast | referral hospital (Treichville University) | 80 / consecutive patients admitted with cryptococcal meningitis | 51% (41/80) male | mean 40 (range 26-58) | 100% (80/80) | mean nadir 81 among patients on ART; mean 45 (range 5-130) in ART-naïve | 32% (26/80) on ART | 14% (11/80) with GCS ≤9; also noted 60% (48/80) with "consciousness disorder" | positive CSF India ink, CrAg, and/or culture | most (86%) treated with AmB induction and FLU consolidation; 14% treated with FLU monotherapy at 800 mg/day x7 days then 400 mg/day | NS | 46% (37/80) received ≥1 therapeutic lumbar puncture | In-hospital |  |
| Baldassarre 2014 | 2008-2009 | retrospective cohort | Kenya | referral and district hospitals (Kenyatta and Mbagathi) | 76 / HIV-infected, ≥13 years of age with diagnosis of cryptococcal meningitis | 60% (46/76) male | median 35 (range 20-53) | 100% (76/76) | median 31 (range 1-468) | 25% (19/76) current or prior ART | NS | positive CSF India ink and/or CrAg | majority received AmB + FLU: 51% (39/76); 30% (23/76) AmB alone; 12% (9/76) FLU; 7% (5/76) received no antifungal therapy | 8 started on ART during hospitalization | note therapeutic lumbar punctures performed in some patients | In-hospital | laboratory monitoring uncommon: 38% (29/76) with no CBC and 54% (41/76) without baseline creatinine and electrolytes |
| Bamba 2012 | 2002-2010 | retrospective cohort | Burkina Faso | referral hospital (Bobo Dioulasso University) | 61 / hospital based cohort of patients who underwent lumbar puncture evaluation | 62% (38/61) male [sex ratio 1.7] | median 32.9 (SD 4.3) | 100% (61/61) | median 56 (range 13-387) | NS | 13% (8/61) with confusion | positive CSF India ink | AmB dose NS x2 weeks; followed by FLU 800 mg/day x1 day followed by FLU 400 mg/day x6-8 weeks | NS | NS | In-hospital |  |
| Bergemann 1996 | 1994-1995 | retrospective cohort 1994 / prospective cohort 1995 | South Africa | university hospital (Baragwanath Hospital) | 37 / adults on the medical and surgical wards with abnormal CSF examinations suggestive of meningitis | 57% (21/37) male | NS for cryptococcal meningitis; mean 36 (range 11-80) in overall cohort | 100% (37/37) | NS | not available | NS | positive CSF culture | NS | not available | NS | In-hospital |  |
| Berhe 2012 | 2002-2009 | retrospective cohort | Ethiopia | referral hospital (Tikur Anbesa Hospital) | 77 / HIV-infected, ≥13 years of age admitted with neurological manifestations of HIV/AIDS | NS for cryptococcal meningitis; 51% (176/347) male overall | NS for cryptococcal meningitis; mean 35 (range 14-65) overall | 100% (77/77) | mean 56 | NS for cryptococcal meningitis; 16% (54/347) overall with history of ART use | 36% (28/77) cryptococcal meningitis cases with altered sensorium | Positive CSF India ink | mix of FLU and AmB, doses NS; 58% (45/77) FLU; 42% (32/77) AmB; no difference in mortality found between groups | NS for cryptococcal meningitis; ART started in 135 patients overall who were discharged alive (71%) without timing specified | NS | In-hospital |  |
| Beyene 2017 | 2014-2016 | prospective cohort | Ethiopia | referral hospital clinics (Asella and Adama) | 34 / CrAg screening cohort of ART-naive or ART- defaulter (>3 months) patients >14 years of age with CD4 ≤150/µL; CrAg-positive patients offered lumbar puncture evaluation | 56% (19/34) male | all >14 years | 100% (34/34) | median 31 (IQR 15-50) | 76% (26/34) ART-naive; 24% (8/34) defaulters | 38% (13/34) with altered mentation | 91% (31/34) with positive CSF CrAg; 9% (3/34) of CrAg-positive patients with overt meningitis and treated without CSF evaluation | FLU 1200 mg/day x2 weeks; then FLU 800 mg/day x8 weeks; then FLU 200 mg/day maintenance | ART initiated between 4 and 8 weeks | Lumbar punctures at discretion of local providers | 3 months | from observational CrAg screening cohort study; note small proportion of patients (3/34) treated based on blood CrAg testing and symptoms of meningitis without confirmatory lumbar puncture - included as only small proportion of these presumptive cases; majority of CrAg-positive patients (65%) of CrAg positive patients already had symptoms of meningitis (unlike Wake 2018 Clin Infect Dis where majority of patients asymptomatic) |
| Gaskell 2014 | 2012-2013 | prospective cohort | Malawi | referral hospital (Queen Elizabeth Central) | 47 / age ≥16 years admitted with first episode of cryptococcal meningitis; patients excluded if lost to follow-up (5), if *Cryptococcus neoformans* isolated from blood only (3), or switched to AmB induction by clinical team (3) | 51% (24/47) male | median 35 (IQR 32-40) | 100% (47/47) | median 36 (IQR 17-62) | 45% (21/47) on ART | 24% (11/46) with GCS<14 | positive CSF India ink and/or culture | FLU 1200 mg/day x2 weeks; then FLU 400 mg/day x8 weeks; then FLU 200 mg/day maintenance | patients not already on ART initiated 4 weeks after diagnosis of cryptococcal meningitis | routine practice therapeutic lumbar puncture when symptoms suggested raised intracranial pressure; due to staffing issues and lack of manometers clinicians generally couldn't follow guidelines | 10 weeks | enrolled patients prospectively in study but would consider "routine care cohort;" patients reviewed by investigators on admission and at 4 and 10 weeks with in-hospital management apparently at discretion of regular clinical team with treatment provided according to national guidelines |
| Gbangba-Ngai 2014 | 2006-2009 | retrospective cohort | Central African Republic | 2 hospitals in Bangui | 122 / patients >16 years diagnosed with cryptococcal meningitis by India ink stain and/or culture | male: female ratio 0.7 | mean 35 (range 18-69) | 99% (121/122) | mean 30 (range 2-363) | 14% (17/121) on ART | 45% (54/121) with altered consciousness | positive CSF India ink and/or culture | AmB used in 84% (duration NS); 3 received FLU only; 9 died before diagnosis made; note consolidation with FLU in 15 patients | NS | NS | In-hospital |  |
| Hiesgen 2017 | 2012-2013 | retrospective cohort | South Africa | referral hospital (Kalafong) | 87 / cryptococcal meningitis identified by screening discharge summaries | 55% (48/87) male | median 34 (IQR "13") | 99% (86/87) | median 52 (IQR 40-65) | 24% (21/87) on ART; 21% (18/87) defaulters | 29% (29/100) with confusion on admission (including repeat admissions for 87 patients) | positive CSF India ink, CrAg, and/or culture | standard AmB 1 mg/kg/day and FLU 800 mg/day x14 days; treatment deviation occurred in 37 patients (as AmB and FLU occasionally unavailable) | NS | repeat LPs occurred in 38% (38/100) of admissions (up to 6 therapeutic lumbar punctures) | In-hospital | noted renal dysfunction or electrolyte abnormalities that were not appropriately addressed (e.g. with intravenous fluids or electrolyte supplementation) occurred in 10 patients |
| Jarvis 2010 | 2007-2008 | prospective cohort | South Africa | referral hospital (GF Jooste) | 228 / patients with laboratory-confirmed cryptococcal meningitis | 50% (113/228) male | median 34 (IQR 29-41) ART-naive; median 33 (IQR 28-39) of those on ART at baseline | 100% (228/228) | median 36/µL (IQR 17-82) ART-naive; median 38 (IQR 18-78) on ART | 20% (45/228) on ART | 44% (100/228) with abnormal mental status | positive CSF India ink, CrAg, and/or culture | standard induction therapy AmB 1 mg/kg/day x2 weeks; as part of ongoing studies, 71 patients received additional induction therapies including 61 5FC, 39 interferon-gamma, and 8 FLU, all with AmB) | timing NS | NS | In-hospital | minority of patients were included from a randomized-controlled trial (Jarvis AIDS 2012) of adjunctive interferon-gamma therapy for cryptococcal meningitis; mostly observational cohort data, however, and included |
| Kisenge 2007 | 2005-2006 | prospective cohort | Tanzania | referral hospital (Kilimanjaro Christian Medical Centre) | 40 / HIV-infected aged 13-65 years admitted to inpatient medical ward with headache and/or altered mental status (GCS<14) | 55% (22/40) male | median 41 (range 14-65) | 100% (40/40) | median 39 (93% [37/40] <100) | 18% (7/40) on ART | 73% (29/40) with GCS<14 | positive CSF India ink and/or CrAg | FLU 800 mg/day x2 weeks; then 400 mg/day x4 weeks; then FLU maintenance | ART either continued or initiated; do not specify timing of ART or number of ART-naive patients initiated | NS | In-hospital |  |
| Kouakou 2017 | 2008-2011 | retrospective cohort | Ivory Coast | referral hospital (Treichville University) | 46 / HIV-infected adults (≥18 years) with diagnosed cryptococcal meningitis treated with FLU 800 or 1200 mg/day; excluded patients treated with AmB | 54% (25/46) male | median 40 (IQR 26-58) | 100% (46/46), including 1 patient with dual HIV-1 and HIV-2 | median 43 (IQR 5-103) | 54% (25/46) on ART | 17% (8/46) with GCS ≤12 | positive CSF India ink, CrAg, and/or culture | 63% (29/46) received FLU 1200 mg/day x2 weeks; then 800 mg/day x8 weeks; 37% (17/46) received FLU 800 mg/day x2 weeks; then 400 mg/day x8 weeks; both groups followed by FLU maintenance | mean delay 30 days (SD 8) | 91% (42/46) had therapeutic lumbar puncture | Presumably in-hospital | study excluded patients treated with amphotericin B; slight overlap August - December 2008 with Aoussi 2012 study; unlikely to be significant overlap in number of patients in these small studies so both included (this study also included patients treated only with fluconazole whereas most patients in other study treated with AmB, so likely insignificant overlap) |
| Lessells 2011 | patients admitted to hospital in 2007 | retrospective cohort | South Africa | district hospital (Hlabisa) | 74 / ≥16 years with confirmed cryptococcal meningitis and complete medical records | 47% (35/74) male | median 35 (IQR 30-44) | 64% (47/74) known HIV-infected on admission | median 49 (IQR 16-86) | 18% (13/74) on ART | 34% (25/74) with reduced consciousness | positive CSF India ink and/or CrAg | majority received FLU (dose NS); 35% (26/74) received AmB-based therapy (dose NS); consolidation therapy with FLU (dose NS) | 7 patients initiated ART during admission (timing NS) | 15% (11/74) with documented repeat lumbar puncture | up to 2 years | of 44 patients discharged alive, 26 patients lost to follow-up over 2 years and only 11% (8/74) known to be alive at 2 years; noted only 6 patients received full 14-day course of AmB |
| Luma 2013 | 2004-2009 | retrospective cohort | Cameroon | referral hospital (Douala) | 75 / HIV-infected adults (≥18 years) diagnosed with central nervous system disease | NS for cryptococcal meningitis; 54% (162/300) male in full cohort | NS | 100% (75/75) | median 23 (IQR 10-61) | NS | 0% (0/75) with documented altered sensorium | positive CSF India ink | NS | NS | NS | In-hospital |  |
| Majwala 2013 | 2010 | prospective cohort | Uganda | referral hospital (Mbarara) | 35 / adults (≥18 years) admitted to medical ward and clinically diagnosed with meningitis by admitting medical team | 54% (19/32) male | mean 35 (SD 11) | 100% (34/34) with known status | NS for cryptococcal meningitis; median 78 (IQR 21-177) in full cohort on ART | NS for cryptococcal meningitis cases; 35% (40/116) in full cohort on ART | mean GCS 13 (SD 3) | positive CSF India ink and/or CrAg | NS | NS | NS | 30 days |  |
| Mamuye 2016 | 2013-2014 | prospective cohort | Ethiopia | referral hospital (Tikur Anbesa) | 16 / HIV-infected adults (≥18 years) admitted to internal medicine or medical intensive care unit regardless of CD4 count screened for blood/urine CrAg; those with positive result offered lumbar puncture | NS for cryptococcal meningitis; 72% (13/18) male among all CrAg-positive | NS for cryptococcal meningitis; mean 34.2 (SD 6.5) among 18 CrAg-positive | 100% (16/16) | median 46 (IQR 23-74) | NS for cryptococcal meningitis; 78% (14/18) among all CrAg-positive | NS for cryptococcal meningitis; 39% (7/18) with altered mental status among all CrAg-positive | positive CSF India ink and/or CrAg (1 diagnosed clinically with positive blood CrAg) | 88% (14/16) received FLU 1600 mg/day or 1200 mg/day duration NS; 2 treated with AmB dose and duration NS | NS | NS | NS | part of a hospital-based CrAg screening study - most of patients who were CrAg-positive had symptoms of meningitis already; two patients had cryptococcal antigenemia without central nervous system disease and excluded from mortality estimate and 1/16 CrAg-positive patients diagnosed with cryptococcal meningitis on clinical grounds but did not have a lumbar puncture |
| Marais 2016 | 2009-2011 | prospective cohort | South Africa | referral hospital (GF Jooste) | 19 / HIV-infected ART-naive adults (≥18 years) with TB meningitis; enrolled cryptococcal meningitis patients as control group (evaluated once at enrolment) | 58% (11/19) male | median 34 (IQR 27-39) | 100% (19/19) | median 55 (IQR 23-77) | 53% (10/19) on ART | 11% (2/19) with confusion | positive CSF India ink or CrAg, confirmed by culture | AmB 0.7 mg/kg/day x2 weeks; then FLU 400 mg/day x8 weeks; then FLU 200 mg/day maintenance | NS timing of ART | NS | 9 months | controlled TB meningitis cohort; investigators enrolled patients with cryptococcal meningitis as a control group but did not manage these patients |
| Mbuagbaw 2006 | 2001-2005 | cross-sectional audit | Cameroon | university hospital (Yaounde University Teaching) | 33 / patients diagnosed with cryptococcal meningitis by India ink stain | 64% (21/33) male | mean 40 (range 22-65) | 97% (32/33) | mean 30 (range 1-128); median 8 | NS | 27% (9/33) with altered consciousness | positive CSF India ink | FLU 800 mg/day duration NS; then 400 mg/day duration NS | NS | NS | In-hospital |  |
| McCarthy 2006 | 2002-2004 | prospective surveillance study | South Africa | GERMS-SA surveillance data; Gauteng Province health facilities including 7 academic, 18 regional , 1 military hospital, 3 mine-funded hospitals and private clinics | 2753 / patients at facilities with isolation of *Cryptococcus* by culture or positive CSF India ink or CrAg or positive histopathology specimen | 49% male (numbers not provided) | median 34 (range 1 month - 74) | 50% (1375/2753) with known HIV | median 37/µL (range 0-955) | NS | 31% (853/2753) with altered mental status | positive CSF India ink, CrAg, and/or culture; positive culture of blood with or without positive CSF testing | of those who received therapy, 72% (1770/2460) received FLU (dose NS); 18% (454/2460) AmB; 9% (234/2460) AmB and FLU; < 1% (2/2460) AmB and non-FLU drug); 9% (293/2753) received no anti-fungal therapy while hospitalized and 6% (162/2753) received no anti-fungal therapy at any time | 4 cases known to be receiving ART | NS | In-hospital |  |
| Meda 2014 | 2009-2012 | prospective cohort | Tanzania | referral hospital (Bugando) | 99 from combined cohorts / adult (≥18 years) with first episode of cryptococcal meningitis confirmed by CSF CrAg testing | 56% (55/99) male | median 34 (31-40) | 100% (99/99) | median 46 (IQR 23-86) | 40% (20/50) with information available | 64% (32/50) with altered mental status | positive CSF CrAg | FLU 1200 mg/day x2 weeks; then FLU 400 mg/day x10 weeks; then FLU 200 mg/day maintenance | NS | study included historic cohort of 64 patients and second cohort of 35 after implementation of a hospital policy for scheduled lumbar punctures at days 0, 3, 7, and 14 with daily therapeutic lumbar punctures for intracranial pressure >20 cm H2O scheduled lumbar punctures | 30 days | mortality lower in later cohort that received regular lumbar punctures through hospital-wide policy; however, study investigators did not manage the patients under the new hospital-wide protocol |
| Meiring 2012 | 2005-2007 | prospective surveillance study | South Africa | GERMS-SA surveillance data; 181 clinical microbiology laboratories in public, private, military, and mining sectors; clinical data obtained from 21 enhanced surveillance sites | 4378 adults (≥15 years) with clinical data (4326 with mortality data) / case defined as positive CSF India ink, CrAg (of CSF, serum, or urine) or culture of *Cryptococcus* species for any specimen site; study compared child (<15 years) and adult cases of cryptococcosis | 46% (2003/4373) male | All ≥15 years | 99% (3451/3481) | 63% (1221/1943) with CD4 <50 | 14% (436/3198) on ART | NS | positive CSF India ink, CrAg (from CSF, blood, or urine), and/or positive *Cryptococcus* culture at any site | 45% (1948/4303) FLU, 43% (1861/4303) AmB, 12% (494/4303) no treatment prescribed in hospital; noted 93% (2399/2576) survivors discharged on FLU | NS | NS | In-hospital | surveillance study comparing characteristics of adult and paediatric cryptococcal meningitis cases in South Africa |
| Meiring 2016 | 2012-2013 | prospective surveillance study | South Africa | GERMS-SA surveillance data; observational cohort including 25 hospitals | 642 with clinical outcomes data / included patients with confirmed cryptococcal meningitis by positive CSF India ink, CrAg (CSF or serum), or positive culture at any site | 55% (352/640) male | NS | 100% (642/642) | 78% (351/452) with CD4<100 | 37% (240/642) on ART | 35% (167/483) with recorded GCS <15 | positive CSF India ink, CrAg (CSF or blood), and/or positive culture at any site | state that national guidelines AmB 1 mg/day/day and FLU 800 mg/day x2 weeks; 82% (524/642) received AmB regimen | NS | NS | In-hospital | monitoring details provided: 64% (333/524) received baseline haemoglobin, potassium, and creatinine; 44% (232/524) with serum potassium and creatinine checked bi-weekly, 27% (142/524) with haemoglobin checked weekly according to national guidelines |
| Millogo 2004 | 1999-2001 | retrospective cohort | Burkina Faso | referral hospital (Bobo Dioulasso University) | 36 / hospital based cohort of patients diagnosed with cryptococcal meningitis | 67% (24/36) male | median 34 (range 25-49) | 100% (36/36) | range 50-476 (only obtained CD4 count in 6 patients) | NS | 39% (14/36) with impaired consciousness | positive CSF India ink | FLU 400 mg/day (duration NS) | NS | NS | In-hospital |  |
| Moosa 1997 | 1991-1994 | retrospective cohort | South Africa | referral hospital (King Edward) | 44 HIV-positive (65 overall) / HIV-positive or HIV-negative diagnosed with cryptococcal meningitis by microscopy, culture, or histopathology; CSF CrAg performed on all patients | 39% (17/44) male | mean 31 (SD 8) | 100% (44/44) | performed on few patients | not available | 36% (16/44) with abnormal mental status | positive microscopy, culture, or histopathology evidence of meningitis; CrAg testing performed routinely | note patients treated with AmB (with or without 5FC) or with FLU but treatment breakdown unknown due to poor records | not available | NS | In-hospital | limited to HIV-positive patients to be consistent with other studies of cryptococcal meningitis in which all or almost all HIV-positive |
| Namutebi 2013 | 2001 | prospective cohort | Uganda | referral hospital (Mulago) | 22 / hospitalized adults (≥18 years) with HIV and receiving ART for at least 2 weeks; excluded patients who had interrupted ART for at least a month prior to admission | NS for cryptococcal meningitis patients | NS for cryptococcal meningitis patients | 100% (22/22) | median 37 (IQR 14-71) | 100% (22/22) on ART | NS | NS | NS | 100% (22/22) on ART at enrolment for at least 2 weeks | NS | In-hospital | study of patients on ART admitted to hospital; 2 patients suspected of having cryptococcal meningitis immune-reconstitution inflammatory syndrome |
| Nyazika 2016 | 2013-2014 | prospective cohort | Zimbabwe | referral hospital (Parirenyatwa) | 55 with cryptococcal meningitis / consenting adults with signs and symptoms of meningitis | 60% (33/55) male | median 36 (IQR 30-43) | 100% (55/55) | median 24 (IQR 12-40) | 55% (30/55) on ART | NS | positive CSF India ink, CrAg, and/or culture | NS | NS | NS | In-hospital | cohort evaluating genetic diversity of *Cryptococcus.* Isolates |
| Oumar 2008 | 2001-2002 | prospective cohort | Mali | infectious disease unit of urban hospital (l'hôpital pour Point G) | 17 / patients in infectious disease unit diagnosed with cryptococcal meningitis by India ink | NS | mean 34 (range 18-60) | 82% (14/17) including 1 with dual HIV-1 and HIV-2 infection | mean 49 (range 1-237) | not available | NS | positive CSF India ink | AmB x2 weeks (dose NS); then consolidation with FLU x6-8 weeks (dose NS); then FLU maintenance | NS | NS | In-hospital |  |
| Patel 2018 | 2012-2014 | retrospective cohort | Botswana | referral hospital (Princess Marina) | 236 (first episode) / diagnosed with cryptococcal meningitis | 69% (163/236) male | median 36 (IQR 32-42) | 100% (236/236) | median 39 (IQR 17-83) | 45% (81/181) with documentation on ART at diagnosis | 40% (62/155) GCS <15 with documented status | positive CSF India ink, CrAg, and/or culture | standard therapy during study period AmB 1 mg/kg/day and FLU 800 mg/day x2 weeks; then FLU 400 mg/day consolidation; then 200 mg/day maintenance | NS | of 283 overall episodes in 236 patients, 54% (154/283) without documented therapeutic lumbar puncture | up to 1 year | over half of patients missed at least 1 dose of AmB; 20% missed single dose, 12% 2 doses, and 21% 3 or more doses |
| Rothe 2013 | 2010-2011 | prospective cohort | Malawi | referral hospital (Queen Elizabeth Central) | 60 / hospitalized with first episode of cryptococcal meningitis | 55% (33/60) male | median 32 (IQR 29-39) | 100% (60/60) | median 37 (range 2-234) [only known for 32/60] | 36% (13/36) on ART among those with ART status known | 24% (14/58) with GCS <14 | positive CSF India ink and/or culture | FLU 800 m/day x2 weeks; then 400 mg/day x6 weeks; then 200 mg/day | started at 4 weeks in ART-naive | lumbar puncture performed if patient had evidence of elevated intracranial pressure (not routine) | 12 months |  |
| Schutte 2000 | 1994-1998 | prospective cohort | South Africa | neurology wards at referral hospital (Pretoria Academic) | 24 with cryptococcal meningitis / headache with or without meningism together with "active" CSF and age >13 years | 58% (14/24) male | mean 37 (range 25-62) | 92% (22/24) | mean 86 for cryptococcal meningitis cases (16/24 with known CD4) | not available | NS | positive CSF culture | NS | not available | NS | In-hospital |  |
| Scriven 2016 | 2012-2014 | prospective cohort | South Africa | three hospitals in Cape Town (GF Jooste, Khayelitsha, and Mitchell's Plain) | 60 / consecutive adults (≥18 years) with first episode of HIV-associated cryptococcal meningitis enrolled within 48 hours of starting antifungal therapy | 55% (33/60) male | median 36 (IQR 30-43) | 100% (60/60) | median 34 (IQR 13-76) | 28% (17/60) on ART | 18% (11/60) with altered consciousness | NS (appears all CSF culture-positive) | AmB 1 mg/kg/day and FLU 800 mg/day x2 weeks; then FLU 400 mg/day x10 weeks; then FLU 200 mg/day maintenance | started at 4 weeks in patients not on baseline ART | noted lumbar punctures performed at the discretion of the attending clinicians | 12 weeks |  |
| Siddiqi 2014 | 2010-2012 | prospective cohort | Zambia | referral hospital (University Teaching Hospital Lusaka) | 64 with cryptococcal meningitis / consecutive HIV-infected adults who presented to emergency department with symptoms suggestive of CNS infection requiring a lumbar puncture | NS for cryptococcal meningitis; overall 51% (169/331) male | median 37 (IQR 32-42) men and 34 (IQR 30-41) female overall | 100% (331/331) overall | median 81 (IQR 30-156) men and 99 (IQR 43-217) in women overall | 35% (117/331) overall | NS | Positive CSF India ink and/or CrAg | standard induction therapy with AmB (dose and duration NS) | NS | NS | In-hospital |  |
| Siika 2008 | 2005-2006 | prospective cohort | Kenya | referral hospital (Moi) | 68 with cryptococcal meningitis / patients enrolled in Academic Model for Prevention and Treatment of HIV/AIDS (AMPATH) ambulatory HIV clinics who were admitted to hospital | NS for cryptococcal meningitis; overall 38% of 495 patients male | median 38 (range 19-74) overall | 100% (62/62) | NS for cryptococcal meningitis; overall 53% with CD4<100 cells before admission | NS for cryptococcal meningitis; overall 76% (375/495) had initiated ART before hospitalization | NS | NS | NS | 19% (92) not already on ART initiated ART during hospitalization | NS | In-hospital |  |
| Sogbanmu 2014 | 2011-2012 | retrospective cohort | South Africa | general hospital (Mthatha) | 57 / adults (>18 years) with first episode of cryptococcal meningitis diagnosed by lumbar puncture | 30% (17/57) male | mean 36 (range 21-60) | 100% (57/57) | median 77 (range 51-100) | 39% (22/57) on ART | 53% (30/57) presented with confusion | positive CSF India ink, CrAg, and/or culture | 89% (51/57) treated with AmB B 1 mg/kg/day x2 weeks; 11% (6/57) FLU 800 mg/day IV because AmB stockout; consolidation FLU 400 mg/day x8 weeks; then 200 mg/day | NS | 18% (10/57) had repeat LPs performed 1 week apart | In-hospital mortality (first 10 days) | 53% (27/50) of patients who received AmB had renal function monitoring during therapy |
| Soumare 2005 | 2001-2003 | cross-sectional audit | Senegal | referral hospital (Fann Teaching) | 37 cases cryptococcal meningitis / patients admitted with ≥1 one: syndrome consistent with meningitis/encephalitis, abnormal CSF, presence of infectious agent in CSF, or abnormal imaging on CT head | NS for cryptococcal meningitis (M/F sex ratio 1.38 overall) | mean 33 (range 2-85) overall | 78% (29/37) mentioned as HIV-infected among cryptococcal meningitis cases although not all likely tested | NS | NS | NS for cryptococcal meningitis cases | NS | NS | NS | NS | In-hospital |  |
| Steele 2010 | 2005-2006 | prospective cohort | Botswana | referral hospital (Princess Marina) | 96 / HIV-infected adults (≥18 years) with confirmed cryptococcal meningitis | 60% (58/96) male | mean 39 (range 26-57) of *C. gattii* infected and 35 (range 21-54) C. neoformans infected | 100% (96/96) | median 45 (IQR 24-67) *C. gattii* infected and 36 (IQR 16-85) *C. neoformans* infected | 30% (29/96) on ART | 16% (15/96) with abnormal GCS | positive CSF CrAg, and/or culture | AmB 1 mg/kg/day x2 weeks; 64% (61/96) received full 2-week course; consolidation FLU 400 mg/day x8 weeks | NS | NS | In-hospital | Study comparing clinical features between *Cryptococcus neoformans* and Cryptococcus gattii infections |
| Trachtenberg 2007 | 2002 | prospective cohort | Uganda | referral hospital (Mulago) | 23 with cryptococcal meningitis / consenting adults (≥18 years) admitted within 24 hours with presumed CNS infection | 51% (51/100) male overall | NS for cryptococcal meningitis; mean 33 (range 18-53) overall | NS for cryptococcal meningitis | NS | 4% (4/100) patients on ART at baseline overall | NS for cryptococcal meningitis | positive CSF India ink and/or CrAg | NS | NS | NS | In-hospital |  |
| Traore 2015 | 2002-2011 | cross-sectional audit | Guinea | neurology unit of referral hospital (l’hôpital national Donka) | 50 with cryptococcal meningitis / patients admitted to specialty unit of hospital with lymphocytic meningitis and HIV | NS for cryptococcal meningitis; 52% (44/85) male overall | NS for cryptococcal meningitis patients; mean 32 (range 16-60) overall | 100% (50/50); including some dual-infected with HIV-1 and HIV-2 | NS for cryptococcal meningitis; overall mean 140 in 13/85 patients with CD4 tested | NS | 4% (2/50) reported with coma | positive CSF India ink and culture | NS | NS | NS | In-hospital | note that all patients had lymphocytic meningitis |
| von Pressentin 2012 | 2009-2010 | cross-sectional audit | South Africa | 1 referral, 3 district, and 1 TB hospital | 25 / HIV-positive ≥13 years diagnosed with cryptococcal meningitis | 52% (13/25) male | mean 40 for males, 33 for females (overall 37) | 100% (25/25) | mean 131 for males, 82 for females (overall 108) | 20% (5/25) on ART | NS | NS | 84% (21/25) received AmB; 3 did not because died before could receive and 1 because of renal dysfunction; consolidation with high-dose FLU x8 weeks | 3 patients initiated ART within 4 weeks of hospitalization (3/5 eligible) | NS | up to 8 weeks post-discharge | less than half met minimum standard of renal monitoring: U and E, Mg while on AmB |

List of abbreviations: 5FC = flucytosine; AmB = amphotericin B; ART = antiretroviral therapy; CrAg = cryptococcal antigen; CSF = cerebrospinal fluid; CT = computed tomography; FLU = fluconazole; GCS = Glasgow coma scale; H2O = water; IQR = interquartile range; NS = not specified; SD = standard deviation

**Routine care cryptococcal meningitis studies - outcomes:**

| First author | Country | Treatment drugs | Dose and duration | 2-wk mortality | In-hospital mortality | Mean/median length-of-stay (if in-hospital mortality) | 10-wk mortality | 6-mo mortality | Details of loss to follow-up | Notes |
| --- | --- | --- | --- | --- | --- | --- | --- | --- | --- | --- |
| Adeyemi 2015 | South Africa | AmB | guidelines AmB 1 mg/kg/day x2 weeks; consolidation fluconazole 400 mg/day x8 weeks then 200 mg/day maintenance | 71/127 |  |  |  |  | NS |  |
| Agaba 2011 | Nigeria | NS | NS |  | 16/31 | median 18 days (including majority without cryptococcal meningitis) |  |  | NS | limited documentation of treatment and management details |
| Aoussi 2012 | Ivory Coast | AmB | 86% (69/80) treated with AmB; 14% (11/80) with fluconazole monotherapy |  | 33/80 | mean 12 days (range 5-30) |  |  | NS |  |
| Baldassarre 2014 | Kenya | AmB+FLU | majority AmB+FLU: 51% (39/76); 30% (23/76) AmB alone: 12% (9/76) FLU; 7% (5/76) no antifungal therapy |  | 30/76 | median 10 days (range 2-73) |  |  | NS |  |
| Bamba 2012 | Burkina Faso | AmB | AmB dose NS x2 weeks; then FLU 800 mg/day x1 day then 400 mg/day x6-8 weeks |  | 17/61 | mean 27 (range 13-132) |  |  | NS |  |
| Bergemann 1996 | South Africa | NS | NS |  | 16/37 | NS (note 2-week mortality was 10/37) |  |  | NS |  |
| Berhe 2012 | Ethiopia | FLU | 58% (45/77) with FLU; 42% (32/77) with AmB (no difference in mortality between groups) |  | 37/77 | NS for cryptococcal meningitis; mean 22 days (range 1-120) for all patients; for deceased patients overall mean stay 26 days (range 7-120) |  |  | NS |  |
| Beyene 2017 | Ethiopia | FLU | FLU 1200 mg/day x2 weeks; then FLU 800 mg/day x8 weeks; then FLU 200 mg/day maintenance | NS | NS |  | 23/34 |  | 3/34 discontinue treatment against medical advice and presumed dead | mortality provided within 3 months |
| Gaskell 2014 | Malawi | FLU | FLU 1200 mg/day x2 weeks; then FLU 400 mg/day x8 weeks; then FLU 200 mg/day maintenance | 16/47 |  |  | 26/47 |  | authors initially excluded 5 patients lost to follow-up during study |  |
| Gbangba-Ngai 2014 | Central African Republic | AmB | AmB dose and duration NS in 84% of patients; note 15 patients started FLU consolidation |  | 81/122 | mean 31 (range 1-83) |  |  | NS |  |
| Hiesgen 2017 | South Africa | AmB+FLU | standard AmB 1 mg/kg/day and FLU 800 mg/day x2 weeks; treatment deviation occurred in 37 patients (when AmB and FLU unavailable) |  | 25/87 | NS |  |  | NS | mortality provided for first admission alone (2 additional deaths occurred during repeat admissions) |
| Jarvis 2010 | South Africa | AmB | standard induction therapy AmB 1 mg/kg/day x2 weeks; then FLU 400 mg/day x8 weeks; then maintenance FLU; as part of ongoing studies, 71 patients received additional induction therapies including 61 5FC, 39 interferon-gamma, and 8 FLU, all with AmB) |  | 70/228 | median 15 days (IQR 13-20) overall; for those who died median 10 days in ART-experienced and 13 days in ART-naive |  |  | NS | note majority of patients in observational setting; some from phase II RCT |
| Kisenge 2007 | Tanzania | FLU | FLU 800 mg/day x2 weeks; then 400 mg/day x4 weeks; then FLU maintenance |  | 20/40 | NS |  |  | NS |  |
| Kouakou 2017 | Ivory Coast | FLU | 63% (29/46) received FLU 1200 mg/day x2 weeks; then 800 mg/day x8 weeks; 37% (17/46) received FLU 800 mg/day x2 weeks; then 400 mg/day x8 weeks; both groups followed by FLU maintenance |  | 23/46 | NS; 13/46 died <7 days |  |  | NS |  |
| Lessells 2011 | South Africa | FLU | majority received FLU (dose NS); 35% (26/74) received AmB-based therapy; consolidation therapy with FLU (dose NS) |  | 30/74 | NS; note all patients who died during admission died within 30 days |  |  | 0 lost to follow-up on discharge; high loss to follow-up over 2 years following diagnosis | Of 44 patients discharged alive, 26 patients lost to follow-up over 2 years; only 11% (8/74) known to be alive at 2 years |
| Luma 2013 | Cameroon | NS | NS |  | 39/75 | NS |  |  | NS |  |
| Majwala 2013 | Uganda | NS | NS |  | 9/35 | NS for cryptococcal meningitis; mean length of stay non-bacterial meningitis 7 days (SD 7) |  |  | In-hospital loss to follow-up NS | 30-day mortality also included at 52% (14/27) excluding 8 patients with unknown mortality |
| Mamuye 2016 | Ethiopia | FLU | 88% (14/16) received FLU 1600 mg/day or 1200 mg/day duration NS; 2 treated with AmB dose and duration NS |  | 6/16 | NS |  |  | NS |  |
| Marais 2016 | South Africa | AmB | AmB 0.7 mg/kg/day x2 weeks; then FLU 400 mg/day x8 weeks; then FLU 200 mg/day maintenance |  | 3/19 | NS |  | 7/19 | no loss to follow-up | note do not have 6-month mortality data; 7/19 patients with cryptococcal meningitis died within 9 months; also note that patients consented to inclusion in study and low proportion with altered mental status compared to most studies |
| Mbuagbaw 2006 | Cameroon | FLU | FLU 800 mg/day duration NS; then 400 mg/day duration NS |  | 14/33 | noted up to 21 days |  |  | NS |  |
| McCarthy 2006 | South Africa | FLU | of those who received therapy, 72% (1770/2460) received FLU (dose NS); 18% (454/2460) AmB; 9% (234/2460) AmB and FLU; < 1% (2/2460) received Am B and a non-FLU drug); 9% (293/2753) received no anti-fungal therapy while hospitalized and 6% (162/2753) received no anti-fungal therapy at any time |  | 749/2753 | mean 10 days (range, 0–373 days) for hospital survivors; mean 5 days (range, 0–195 days) for those who died in hospital |  |  | NS |  |
| Meda 2014 | Tanzania | FLU | FLU 1200 mg/day x2 weeks; then FLU 400 mg/day x10 weeks; then FLU 200 mg/day maintenance | 55/99 |  |  |  |  | NS | combined two cohorts; mortality lower with hospital-wide policy of scheduled lumbar punctures for patients with cryptococcal meningitis; 2-week mortality (55/99) obtained through inspection of Kaplan-Meier curves in manuscript |
| Meiring 2012 | South Africa | (mix FLU and AmB) | 45% (1948/4303) FLU, 43% (1861/4303) AmB, 12% (494/4303) no treatment prescribed in hospital; noted 93% (2399/2576) survivors discharged on FLU |  | 1440/4326 | 33% (1428/4314) <7 days; 32% (1362/4314) 7-13; 20% (883/4314) 14-20; 15% (641/4314) ≥21 |  |  | NS |  |
| Meiring 2016 | South Africa | AmB + FLU | AmB 1 mg/day/day and FLU 800 mg/day x2 weeks; 82% (524/642) received AmB regimen |  | 187/642 | NS |  |  | NS | case-fatality 25% (134/524) of these treated with AmB |
| Millogo 2003 | Burkina Faso | FLU | FLU 400 mg/day (duration NS) |  | 15/36 | mean 16 (range 1-29) |  |  | NS |  |
| Moosa 1997 | South Africa | FLU | note patients treated with AmB (with or without flucytosine) or with FLU but treatment breakdown unknown due to poor records ; assume most treated with FLU during time period |  | 28/44 | NS; 2-week mortality also provided of 34% (15/44) |  |  | NS |  |
| Namutebi 2013 | Uganda | NS | NS |  | 8/22 | NS for cryptococcal meningitis; overall median 8 days (IQR 6-13) |  |  | NS |  |
| Nyazika 2016 | Zimbabwe | NS | NS |  | 30/54 | median 18 days (IQR 10-22) |  |  | 1 lost to follow-up (authors did not include this patient in outcomes) [used total of 54] |  |
| Oumar 2008 | Mali | AmB | AmB x2 weeks (dose NS); then consolidation with FLU x6-8 weeks (dose NS); then maintenance |  | 11/17 | length of hospitalization NS; noted 7/17 died within 3 treatment weeks; 4 patients left against medical advice and assumed dead for this analysis; additional 3/17 died after 3rd week of treatment and not included here for short-term mortality |  |  | 4 patients left against medical advice; assume dead here | unclear length of follow-up but provide that 7/17 died within 3 weeks with additional 4/17 refusing care so assume 11/17 for acute mortality unclear when additional 3/17 patients died among those who died after 3 weeks |
| Patel 2018 | Botswana | AmB + FLU | standard AmB 1 mg/kg/day and FLU 800 mg/day x2 weeks; then 400 mg/day x8 weeks; then 200 mg/day maintenance | 60/233 |  |  | 112/224 | 142/219 (1-year mortality) | mortality available for 233 patients (99%) at 2 weeks, 224 patients (95%) at 10 weeks, and 219 patients (93%) at 1 year | 1-year mortality estimate included rather than 6-months; missing outcomes for 7% up to 1 year so likely under-estimate of true mortality of those without follow-up data |
| Rothe 2013 | Malawi | FLU | FLU 800 mg/day x2 weeks; then 400 mg/day x6 weeks; then 200 mg/day |  | 26/60 | 4-week mortality (did not provide 2-week mortality) | 33/60 | 43/56 (1-year mortality excluding patients lost to follow-up between 10 weeks and 1 year) | 6-month mortality form Kaplan-Meier curve (although 4 lost to follow-up between 10 weeks and 1 year); reported mortality 77% (43/56) at 1 year in those with known vital status |  |
| Schutte 2000 | South Africa | NS | NS |  | 9/24 | NS |  |  | NS |  |
| Scriven 2016 | South Africa | AmB + FLU | AmB 1 mg/kg/day and FLU 800 mg/day x2 weeks; then FLU 400 mg/day x10 weeks; then FLU 200 mg/day maintenance | 14/60 |  |  | 22/57 |  | 12-week rather than 10-week mortality; 3 patients lost to follow-up between 2 and 12 weeks |  |
| Siddiqi 2014 | Zambia | AmB | standard AmB therapy (dose and duration NS) |  | 25/64 | NS |  |  | NS |  |
| Siika 2008 | Kenya | NS | NS |  | 24/68 | median length of hospital stay 8 days overall (range 1-44) in survivors and 6 days (range 1-30) in those who died; NS for cryptococcal meningitis |  |  | NS |  |
| Sogbanmu 2014 | South Africa | AmB | 89% (51/57) AmB B 1 mg/kg/day x2 weeks; 11% (6/57) FLU 800 mg/day IV because AmB stockout; consolidation FLU 400 mg/day x8 weeks; then 200 mg/day |  | 17/57 | death within 10 days; for survivors mean 21 days (range 16-28) |  |  | NS |  |
| Soumare 2005 | Senegal | NS | NS |  | 22/37 | median length of hospital stay 8 days overall (range 0-194 days); NS for cryptococcal meningitis |  |  | NS |  |
| Steele 2010 | Botswana | AmB | AmB 1 mg/kg/day x2 weeks; 64% (61/96) received full 2-week course; consolidation with FLU 400 mg/day x8 weeks |  | 18/96 | NS |  |  | NS |  |
| Trachtenberg 2007 | Uganda | NS | NS |  | 11/23 | NS |  |  | NS | limited outcomes to confirmed cases |
| Traore 2015 | Guinea | NS | NS |  | 38/50 | mean length of hospital stay 60.5 days overall (NS for cryptococcal meningitis |  |  | NS |  |
| von Pressentin 2012 | South Africa | AmB | 84% (21/25) received AmB; 3 did not because died before could receive and 1 because of renal dysfunction; consolidation with high-dose FLU x8 weeks |  | 12/25 | NS; noted that 1 additional patient died soon after discharge |  |  | NS | patients followed up to 8 weeks post-discharge; 12 died in hospital and noted that 1 died soon after discharge; otherwise unclear completeness of data through 8 weeks post-discharge |

List of abbreviations: 5FC = flucytosine; AmB = amphotericin B; ART = antiretroviral therapy; CrAg = cryptococcal antigen; CSF = cerebrospinal fluid; CT = computed tomography; FLU = fluconazole; GCS = Glasgow coma scale; H2O = water; IQR = interquartile range; NS = not specified; RCT = randomized-controlled trial; SD = standard deviation

**Clinical trial cryptococcal meningitis studies - details:**

| First author [ref] | Year(s) | Study type | Country | Setting | Patients no. / details | Sex | Age (years) | HIV +ve | CD4 count (cells/µL) | Baseline ART status | Altered mental status | Diagnostic details | Treatment(s) | ART post-dx | Therapeutic management | Follow-up duration | Notes |
| --- | --- | --- | --- | --- | --- | --- | --- | --- | --- | --- | --- | --- | --- | --- | --- | --- | --- |
| Beardsley 2016 | 2013-2014 | RCT | Vietnam, Thailand, Indonesia, Laos, Uganda, and Malawi | 13 hospitals | 226 including only those who did not receive dexamethasone; 55% (124/226) recruited from African centres; / adults (≥18 years) with HIV and microbiologically-confirmed cryptococcal meningitis; exclusion criteria included pregnant, renal failure, gastrointestinal bleeding, >7 days anticryptococcal therapy, on glucocorticoids or requiring for co-existing conditions | 58% (132/226) male including African and Asian patients | median 35 (IQR 30-40) out of 226 Asian and African patients | 100% (226/226) | median 20 (IQR 7-52) out of 226 Asian and African patients | 41% (93/226) on ART | 22% (50/226) with GCS<15 | positive CSF India ink, CrAg, and/or culture, or positive *Cryptococcus* blood culture with clinical meningitis syndrome | AmB 1 mg/kg/day and FLU 800 mg/day x2 weeks; then FLU 800 mg/day x8 weeks; then FLU 200 mg/day maintenance; given with placebo | initial protocol recommended ART initiation 2-4 weeks after start of treatment, later changed to 5 weeks | lumbar puncture on days 1, 3, 7, and 14; more frequently as needed | 6 months | RCT evaluating adjunctive dexamethasone for management of HIV-associated cryptococcal meningitis; trial stopped at interim analysis because dexamethasone associated with worse outcomes on key measures; have included only outcomes of placebo group here as steroids not recommended for treatment of cryptococcal meningitis based on findings from this trial |
| Bicanic 2007 | 2005 | controlled prospective cohort | South Africa | referral hospital (GF Jooste) | 54 / HIV-infected adults (≥21 years) with cryptococcal meningitis diagnosed by CSF India ink or CrAg and confirmed by culture | 26% (14/54) male | median 34 (IQR 29-39) | 100% (54/54) | median 49 (IQR 21-71) | 33% (18/54) on ART | 24% (13/54) with GCS <15 | positive CSF India ink or CrAg confirmed by culture | 91% (49/54) treated with AmB 1 mg/kg/day x 1 week; 9% (5/54) treated with FLU 400 mg/day because of AmB unavailability (2), refusing admission (1), renal toxicity (1), GCS < 10 (1); consolidation FLU 400 mg/day x8 weeks; then 200 mg/day maintenance | In ART-naive, started no sooner than 4 weeks after initiation of antifungal therapy | repeat lumbar punctures on days 3, 7, and 14, or in patients with increased intracranial pressure (>25 cm H2O) | at least 1 year |  |
| Bicanic 2008 | 2005-2006 | RCT | South Africa | referral hospital (GF Jooste) | 64 / HIV-infected adults (≥18 years) diagnosed with first episode of cryptococcal meningitis; exclusion criteria included alanine aminotransferase level >5x upper limit of normal, absolute neutrophil count <500 x10^6 cells/L, platelet count <50,000 x 10^6 platelets/L, pregnant or lactating, previous serious reaction to AmB or 5FC, already receiving antiretroviral therapy | 38% (24/64) male | median 33 (IQR 28-38) | 100% (64/64) | median 38 (IQR 12-69) | 0% (0/64) | 13% (8/64) | positive CSF India ink with culture confirmation | 2 study arms both of which received AmB and 5FC 100 mg/kg/day for 2 weeks; randomized to AmB dose of 0.7 mg/kg/day or 1 mg/kg/day; then consolidation FLU 400 mg/day x8 weeks; then FLU maintenance | ART started 4 weeks from start of antifungal therapy | scheduled lumbar punctures days 3, 7, and 15, and as needed for raised intracranial pressure or headache or other symptoms concerning for raised intracranial pressure | up to 1 year | RCT comparing two different dosing regimens of AmB for 2 weeks both with 5FC; Baseline hematologic, electrolyte, liver and renal function testing; subsequent alternate-day renal function testing and twice-weekly hematologic and liver function testing |
| Chang 2013 | 2009-2011 | controlled prospective cohort | South Africa | recruited in Durban, South Africa | 128 / HIV-infected ART-naïve adults (≥18 years) with first episode of cryptococcal meningitis | 57% (60/106) males limited to those who commenced ART | median 33.5 (IQR 28.0-40.0) in 106 patients who commenced ART | 100% (128/128) | median 35/µL (IQR 11-77) in those who commenced ART | 0% (0/128) | median GCS 15 (IQR 14-15) limited to those who commenced ART | positive CSF India ink and/or CrAg | AmB 1 mg/kg/day x2 weeks; then FLU 400 mg/day x8-12 weeks; then maintenance | ART initiated at median 18 days (IQR 15-22); commenced based on clinical judgment | lumbar puncture performed by local study team as clinically indicated per national guidelines with input from study physician | 24 weeks | considered a "controlled" cohort; patients were co-managed by local team but with regular management input from a member of the study team |
| Jackson 2012 | 2009-2010 | RCT | Malawi | referral hospital (Kamuzu Central) | 40 (3 additional excluded for false positive India ink); ART-naive, HIV-infected adults with first episode of cryptococcal meningitis / exclusion criteria included pregnant or lactating, alanine aminotransferase >1200 IU/L, platelets <50,000 x 10^3/mL, neutrophils <500 x 10^3/mL, any contraindication to study drug, or creatinine >2.5 mg/dL | 65% (26/40) male | median 35 (range 19-52) | 100% (40/40) | median 41 (range 2-258) | 0% (0/40) | 15% (6/40) with GCS < 15 | Positive CSF India ink confirmed by CrAg or culture | Two arms: 1) AmB 1 mg/kg/day x1 week + FLU 1200 mg/day and 5FC 100 mg/kg/day x2 weeks; 2) AmB 1 mg/kg/day x1 week and FLU 1200 mg/day mg/kg/day x2 weeks; consolidation FLU 800 mg/day x2 weeks; then 400 mg/day x8 weeks; then maintenance | ART started at 4 weeks | Scheduled on days 1, 3, 7, and 14; additional therapeutic lumbar punctures as needed for demonstrated or suspected raised intracranial pressure | 10 weeks | Blood count with differential, alanine aminotransferase, alanine aminotransferase, potassium, and creatinine at baseline then three times weekly for first 2 weeks; aspartate aminotransferase and alanine aminotransferase repeated at weeks 4, 6, and 10 |
| Jarvis 2012 | 2007-2010 | RCT | South Africa | referral hospital (GF Jooste) | 31 patients who did not receive adjunctive interferon gamma / Inclusion criteria included ART-naive HIV-infected adult (≥21 years) with positive CSF India ink or CrAg test; exclusion criteria included pregnant or breastfeeding, previous cryptococcal meningitis, alanine aminotransferase > 200 IU/mL, neutrophil count < 500 x 106 cells/L, platelets < 50 x 106 cells/L, previous serious reaction to study drugs or contraindication to study drugs | 52% (16/31) male | median 33 (IQR 29-39) | 100% (31/31) | median 36 (IQR 18-63) | 0% (0/31) | 29% (9/31) with GCS <15 | positive CSF India ink or CrAg | AmB 1 mg/kg/day and 5FC 100 mg/kg/day x2 weeks; then FLU 400 mg/day x8 weeks; then maintenance | protocol for ART initiation between 2 and 4 weeks after initiation of antifungal therapy | scheduled days 1, 3, 7, and 14 and as clinically indicated | 10 weeks; followed up for 1 year but only for C-IRIS monitoring (mortality data at 1 year not reported) | RCT comparing AmB and 5FC for 2 weeks with or without adjunctive interferon gamma; excluding interferon gamma treatment groups as not currently used in care anywhere; alternate-day renal function and electrolyte testing and twice-weekly blood counts and LFTs during first 2 weeks |
| Jarvis 2018 | 2014-2016 | RCT | Botswana and Tanzania | 2 referral hospitals (Princess Marina and Bugando Medical Centre and Sekou Toure) | 21 in control arm that received standard treatment course for cryptococcal meningitis (2 weeks AmB + FLU); enrolled sequential HIV-infected adults ≥18 years with first episode of cryptococcal meningitis, excluded pregnant or lactating patients, patients with a previous serious reaction to study drugs, or patients on antifungal treatment >48 hours | 57% (12/21) | median39 (IQR 34-46) | 100% (21/21) | median 24 (IQR 5-69) | 38% (8/21) | 29% (6/21) with GCS <15 | positive CSF India ink and/or CrAg | L-AmB 3 mg/kg/day and FLU 1200 mg/day x2 weeks; then FLU 800 mg/day x8 weeks; then FLU 200 mg/day maintenance | ART started after 4-6 weeks in ART-naïve | scheduled day 3, 7, and 14; patients with CSF opening pressure >30 cm H2O or symptoms of raised intracranial pressure received daily lumbar punctures | 10 weeks | study comparing short-course liposomal amphotericin regimens with FLU (1, 2, or 3 doses) with traditional 2-week liposomal AmB and FLU regimen; only included 2-week induction regimen |
| Kambugu 2008 | 2001-2002 and 2006 | 2 controlled prospective cohorts | Uganda | referral hospital (Mulago) | 136 from pooled cohorts; HIV-infected ART-naive adults (≥18 years) with confirmed cryptococcal meningitis / exclusion criteria included haemoglobin <5.0 g/dL, creatinine >3.0 mg/dL, or comatose and therefore unable to provide consent | NS | NS | 99% (135/136) [1 negative on repeat testing] | 2001-2002: median 34.5 (IQR 29 - 38); 2006: median 36 (IQR 31 - 42) | 0% (0/136) | 38% presented with altered mental status | first cohort positive CSF culture; second cohort at least 2/3 of: CSF India ink, CrAg, and culture | AmB 0.7 mg/kg/day x2 weeks; then FLU 400 mg/day x8 weeks; then maintenance | first cohort: ART not available; Second cohort: Evaluated in clinic within 1 week of discharge, then started ART 1-2 weeks thereafter | daily lumbar punctures recommended for elevated intracranial pressure; however, most patients refused therapeutic lumbar punctures (therapeutic lumbar punctures only performed in 7%, 10/136) | up to 6 months (second cohort from 2006) | mortality in first cohort (pre-ART era) significantly higher than second cohort; second cohort has 6-month mortality estimates which have not been included as missing outcomes data for 2/3rds of patients in combined cohort |
| Katwere 2009 | 2004-2005 | controlled prospective cohort | Uganda | urban clinic | 50 (38 primary, 12 relapse or IRIS) / HIV-infected adults (≥18 years) presenting to referral hospital clinic with main complaint of headache | 41% (20/49) male | NS for cryptococcal meningitis; median 35 (IQR 30-41) for 180 patients overall | 100% (50/50) | 67% (31/47) <50; 32% (15/47) 50-199; 2% (1/47) ≥200 | NS for cryptococcal meningitis; 29% (53/180) for 180 patients overall | NS | positive CSF India ink, CrAg, and/or culture | 72% (36/50) managed out of hospital with oral FLU (dose NS); 28% (14/50) managed in hospital with AmB (dose NS); no details on consolidation therapy or maintenance | 34% (17/50) patients diagnosed with CM received ART; timing not specified | NS | 4 months | patients followed for 4 months; outcomes NS for 2-weeks or 10-weeks; likely selection bias for patients managed out of hospital with FLU who had lower mortality vs. managed in-hospital with AmB which was associated with higher mortality |
| Lightowler 2010 | 2007 | controlled prospective cohort | South Africa | regional hospital (Ngwelezane) | 186 / included all consenting patients diagnosed with cryptococcal meningitis by culture in 2007 | 50% (92/186) male | median 32 (IQR 27-38) | 74% (138/186) known HIV-positive, others unknown | median 46 (IQR 17-100) | 15% (27/186) on ART | 86% (147/186) with GCS = 15 | positive CSF culture | standard AmB 0.7 mg/kg/day x14 days; 80% (149/186) received AmB including one with FLU; 15% (28/186) received FLU 400 mg/day monotherapy primary due to renal dysfunction (25/28); 5% (9/186) received no antifungal therapy | ART started only after 14 days of treatment, usually in an outpatient clinic after discharge | when possible, lumbar puncture repeated at days 7 and 14; additional lumbar punctures for severe headache (manometers usually unavailable) | 30 days | baseline full blood counts, electrolytes/renal tests and liver function tests; repeat electrolytes/renal function every 48 hours; cannulation sites rotated every 72 hours when able to avoid thrombophlebitis |
| Longley 2008 | 2005-2007 | controlled prospective cohort | Uganda | referral hospital (Mbarara) | 60 / HIV-infected adults (≥18 years) with first episode of cryptococcal meningitis; exclusion criteria included alanine aminotransferase >200 U/L, pregnancy, prior serious reaction to fluconazole, prior fluconazole in the previous month, already on ART | 57% (34/60) male | median 34 (IQR 29-39) | 100% (60/60) | median 12 (IQR 4-32) | 0% (0/60) | 47% (28/60) with GCS<15 | positive CSF India ink confirmed by culture | 30 patients received FLU 800 mg/day x2 weeks in first cohort; then 30 received FLU 1200 mg/day x2 weeks in second sequential cohort; both then FLU 400 mg/day x8 weeks; then maintenance | according to Uganda guidelines, started 1-8 weeks after starting antifungal therapy; median time to ART 5 weeks | scheduled at days 3, 7, and 14; additional lumbar punctures for raised opening pressure or headache or other symptoms suggestive of raised intracranial pressure | up to 6 months (only 2 and 10 week mortality reported) | no difference in mortality between group receiving FLU 800 mg/day and 120 mg/day induction; baseline complete blood counts, electrolyte, urea, creatinine, and alanine aminotransferase; repeated on days 7 and 14 |
| Loyse 2012 | 2006-2008 | RCT | South Africa | 2 hospitals (GF Jooste and Edendale) | 79 (1 additional excluded for prior cryptococcal meningitis) / Inclusion criteria included: HIV infected, ART-naive, ≥ 18 years of age, positive CSF India ink confirmed by culture; exclusion criteria included pregnant or breastfeeding, previous cryptococcal meningitis, alanine aminotransferase> 200 IU/mL, neutrophil count < 500 x 106 cells/L, platelets < 50 x 106 cells/L, previous serious reaction to study drugs or contraindication to study drugs, if >3 doses of AmB received | 49% (39/79) male | median 34 (IQR 29-39) | 100% (79/79) | median 24 (IQR 9-43) | 0% (0/79) | 15% (12/79) with GCS <15 | positive CSF India ink confirmed by pathogen | randomized to: 1) AmB 0.7-1 mg/kg/day and 5FC 100 mg/kg/day x2 weeks; 2)  AmB 0.7-1 mg/kg/day and FLU 800 mg/day x2 weeks; 3) AmB 0.7-1 mg/kg/day and FLU 1200 mg/day x2 weeks; or 4) AmB 0.7-1 mg/kg/day and voriconazole 300 mg twice daily x2 weeks; all then FLU 400 mg/day up to 8 weeks; then maintenance | protocol for ART initiation > 2 weeks after starting antifungal therapy | scheduled days 3, 7, and 14 and as clinically indicated | 10 weeks; followed up for 6 months but no 6 month mortality data | alternate-day renal function, twice-weekly blood counts and LFTs; have combined three AmB and azole drug groups here |
| Makadzange 2010 | 2006-2008 | RCT | Zimbabwe | referral hospital (Parirenyatwa Central) | 26 (excluding 28 in early ART arm); HIV-positive ART-naive adults (≥18 years) first episode of cryptococcal meningitis / exclusion criteria receiving medications that interfere with FLU metabolism, pregnant or lactating, history of hepatic or renal impairment; not residing within 50 kg radius of Harare | 54% (14/26) male | mean 37.5 (SD 6.9) | 100% (26/26) | median 51.5 (IQR 25-69.5) | 0% (0/26) | 27% (7/26) with confusion or poor cognitive function | positive CSF India ink and/or CrAg | FLU 800 mg/day x10 weeks; then maintenance | including group randomized to deferred ART until 10 weeks after start of antifungal therapy | treated by primary medicine service; notes worsening headaches and mental status may trigger repeated lumbar puncture | up to 3 years | RCT on timing of ART for ART-naïve participants enrolled with incident cryptococcal meningitis; randomized to start ART after 3 days or 10 weeks; very high mortality in early ART arm (excluded from this analysis as early ART not recommended in guidelines) |
| Molloy 2018 | 2013-2016 | RCT | Malawi, Zambia, Tanzania, and Cameroon | 9 hospitals in 4 countries | 721 (43 subsequent withdrawn from study) for 678 included / Inclusion criteria included ≥18 years, positive CSF India ink or cryptococcal antigen; exclusion criteria included pregnant or breastfeeding; prior cryptococcal meningitis; >1 dose amphotericin B or >1 treatment dose (1200 mg) or >7 low doses (200 mg) of fluconazole in the past two weeks; previous adverse reaction to study drugs; taking contraindicated concomitant drugs; alanine aminotransferase >5 times upper limit of normal or neutrophil count <500 x 106/L included as late exclusion criteria; patients with elevated creatinine >220 µmol/L the day after enrolment despite hydration withdrawn from the study | 58% (390/678) male | median 36 (IQR 32-43) in oral regimen arm, 38.5 (IQR 32-44) in 1-week AmB arm, 37 (IQR 32-43) in 2-week AmB arm | 100% (678/678) | median 25 (IQR 10-63) in oral regimen arm, 26.5 (IQR 12-63) in 1-week AmB arm, 26 (IQR 10-64) in 2-week amphotericin B arm | 56% (381/678) with baseline ART exposure | 163/678 (24%) GCS <15 | positive CSF India ink and/or CrAg | randomized 2:1:1:1:1 to FLU 1200 mg/day and 5FC 100 mg/kg/day x2 weeks; AmB 1 mg/kg/day x1 week and FLU 1200 mg/day x2 weeks; AmB 1 mg/kg/day and FLU 1200 mg/day x2 weeks; AmB 1 mg/kg/day and 5FC x1 week then FLU 1200 mg/day days 8-14; or AmB 1 mg/kg/day and 5FC x2 weeks; all regimens followed by FLU 800 mg/day until ART started at 4 weeks, then 400 mg/day until 10 weeks, then 200 mg/day maintenance | started at 4 weeks in patients who were ART-naive or defaulted | lumbar punctures scheduled at days seven and 14, with additional daily therapeutic lumbar punctures for patients with high CSF pressure until pressure controlled | 10 weeks |  |
| Muzoora 2012 | 2008-2009 | controlled prospective cohort | Uganda | referral hospital (Mbarara) | 30 / ART naive adults (≥18 years) with first episode of cryptococcal meningitis by CSF India ink confirmed by culture; excluded for alanine aminotransferase >5 times upper limit of normal, pregnant, prior serious reaction to fluconazole, on fluconazole in past month, creatinine >2.5 mg/dL, or already on ART | 70% (21/30) male | median 35 (IQR 33-40) | 100% (30/30) | median 21 (IQR 12-62) | 0% (0/30) | 57% (17/30) with abnormal mental status | positive CSF India ink with culture confirmation | AmB 1 mg/kg/day for 5 days with FLU 1200 mg/day for 2 weeks then 800 mg/day until ART started; then FLU 400 mg/day for 8 weeks, then 200 mg/day consolidation | started at median 33 days (IQR 28-37) | follow-up lumbar puncture on days 3, 7, and 14; patients with high CSF opening pressure and/or headache or other symptoms attributable to raised pressure received additional lumbar punctures | 10 weeks |  |
| Nussbaum 2010 | 2008 | RCT | Malawi | referral hospital (Kamuzu Central) | 40 included in mortality assessment (originally 43 but 3 excluded for breastfeeding, negative confirmatory CSF culture, false diagnosis due to sampling error) / Inclusion criteria included HIV-infected adult, ART-naive, first episode of cryptococcal meningitis; exclusion criteria included pregnant or breastfeeding, previous cryptococcal meningitis, alanine aminotransferase >200 IU/mL, neutrophil count <500x106 /L, platelets <50x106 /L, contraindication to any study medication | 66% (27/41) male | median 36 (range 23-73) | 100% (41/41) | median 21 (range 1-101) | 0% (0/41) | 39% (16/41) with GCS <15 | positive CSF India ink or CrAg, confirmed by culture | randomized to FLU 1200 mg/day and 5FC 100 mg/kg/day x2 weeks or FLU 1200 mg/day x2 weeks; both followed by FLU 800 mg/day after 2 weeks, then 400 mg/day after starting ART at 4 weeks, then 200 mg/day maintenance after 10 weeks of 400mg/day | ART initiated at 4 weeks | lumbar punctures scheduled days 1, 3, 7, and 14 and as clinically indicated | 10 weeks |  |
| Ochieng 2009 | 2007 | controlled prospective cohort | Kenya | referral hospital (Kenyatta) | 70 / HIV-infected adults (≥18 years) with cryptococcal meningitis; exclusion criteria included patient unlikely to survive at least 3 days of the study, contraindication to AmB, patient on nephrotoxin that might exacerbate amphotericin B-related renal toxicity, or uncorrected hypokalaemia at initiation of amphotericin B therapy | 56% (39/70) male | mean 37 (SD 11) | 100% (70/70) | mean 56 (67% [47/70] <50) | NS | NS | positive CSF India ink or CrAg | AmB 0.7 mg/kg/day x2 weeks intended; only 54% (38/70) completed full 2-week course | NS | NS | 2 weeks | controlled cohort, e.g. potassium measured daily first 6 days then alternate days; creatinine and magnesium measured at baseline then days 3, 6, 10, and 14, patients received potassium supplementation and pre-hydration before AmB doses; note possible risk of selection bias - note excluded patients thought would die within 3 days; recruited 84 ppts, 70 enrolled; author assessment should include (similar to exclusion in most RCTs) |
| Rhein 2019 | 2015-2017 | RCT | Uganda | 2 referral hospitals (Mulago and Mbarara) | 231 patients from ASTRO-CM trial who received placebo treatment (not adjunctive sertraline) / HIV-infected adults ≥18 years with diagnosis of cryptococcal meningitis; excluded if already received 3 doses of AmB, had jaundice or known cirrhosis, or pregnant or breastfeeding | 62% (144/231) male | mean 35 (IQR 30-41) in placebo arm | 100% (231/231) | median 13 (IQR 6-40) in placebo arm | 50% (115/231) ART-experienced | 46% (105/231) with GCS <15 | positive CSF CrAg confirmed by culture | standard AmB 0.7-1 mg/kg/day x7-14 days and FLU 800-1200 mg/day; followed by consolidation and maintenance FLU | started around 4 weeks | scheduled at days 1, 3, 7, 10, and 14; as needed for elevated intracranial pressure | 18 weeks | only included placebo arm that did not receive adjunctive experimental treatment with sertraline; ASTRO-CM-specific results obtained from Rhein CROI abstract 2018 as results by group not published yet, later published in Lancet Infect Dis |

List of abbreviations: 5FC = flucytosine; AmB = amphotericin B; ART = antiretroviral therapy; C-IRIS = cryptococcal immune-reconstitution inflammatory syndrome; CrAg = cryptococcal antigen; CSF = cerebrospinal fluid; FLU = fluconazole; GCS = Glasgow coma scale; H2O = water; IQR = interquartile range; LFT = liver function test; NS = not specified; RCT = randomized-controlled trial

**Clinical trial cryptococcal meningitis studies - outcomes:**

| First author | Country | Treatment drugs | Dose and duration | 2-wk mortality | 10-wk mortality | 6-mo mortality | Details of loss to follow-up | Notes |
| --- | --- | --- | --- | --- | --- | --- | --- | --- |
| Beardsley 2016 | Uganda and Malawi | AmB + FLU | AmB 1 mg/kg/day and FLU 800 mg/day x2 weeks; then FLU 800 mg/day x8 weeks; then FLU 200 mg/day maintenance; given with placebo | 23/124 | 51/124 | 62/124 | 0 lost to follow-up | included only outcomes for patients from African health centres |
| Bicanic 2007 | South Africa | AmB (1week) | 91% (49/54) patients treated with AmB 1 mg/kg/day x 1 week; 9% (5/54) treated with FLU 400 mg/day because of AmB unavailability (2), refusing admission (1), renal toxicity (1), GCS < 10 (1); consolidation FLU 400 mg/day x8 weeks; then 200 mg/day maintenance | 9/54 | 19/52 | 30/52 | 2 lost to follow-up between 2 weeks and 10 weeks; 1 lost to follow-up at 1 year (deaths in 33/51 at 1 year with known outcomes) | 1-year mortality outcomes presented in paper - from K-M curve 3 deaths between 6 months and 1 year; 1 lost to follow-up at 1 year assumed between 6 months and 1 year |
| Bicanic 2008 | South Africa | AmB + 5FC | two study arms both of which received AmB and 5FC 100 mg/kg/day for 2 weeks; randomized to AmB dose of 0.7 mg/kg/day or 1 mg/kg/day; then consolidation FLU 400 mg/day x8 weeks; then FLU maintenance | 4/64 | 15/63 | 20/61 (3 lost to follow-up between 2 weeks and 6 months - spreadsheet from T. Bicanic) | 1 patient lost to follow-up within 10 weeks, 3 lost to follow-up within 1 year | 24/61 dead at 1 year (3 lost to follow-up; report 40% mortality in those with known outcomes) |
| Chang 2013 | South Africa | AmB | AmB 1 mg/kg/day x2 weeks; then FLU 400 mg/day x8-12 weeks; then maintenance | 19/128 |  | 40/125 | 19 did before ART could be initiated and 21 died after starting ART by the end of 6-month follow-up; 3 lost to follow-up | 2-week mortality died during antifungal induction therapy before commencement of ART; could not determine 10-week mortality from Kaplan-Meier curves |
| Jackson 2012 | Malawi | AmB (1week) + 5FC + FLU | AmB 1 mg/kg/day x1 week + FLU 1200 mg/day and 5FC 100 mg/kg/day x2 weeks | 2/20 | 6/19 |  | 1 lost to follow-up between 2-10 weeks |  |
| Jackson 2012  (same study as above) | Malawi | AmB (1week) + FLU | AmB 1 mg/kg/day x1 week and FLU 1200 mg/day x2 weeks | 4/20 | 7/20 |  | 0 lost to follow-up |  |
| Jarvis 2012 | South Africa | AmB + 5FC | AmB 1 mg/kg/day and 5FC 100 mg/kg/day x2 weeks; then FLU 400 mg/day x8 weeks; then maintenance | 6/31 | 10/31 |  | 0 lost to follow-up |  |
| Jarvis 2018 | Botswana and Tanzania | AmB + FLU | Liposomal AmB 3 mg/kg/day and FLU 1200 mg/day x14 days; then 800 mg/day x8 weeks; then 200 mg/day maintenance | 2/21 | 6/20 |  | 1 patient lost to follow-up following hospital discharge | note: 9/134 screened died prior to enrolment |
| Kambugu 2008 | Uganda | AmB | AmB 0.7 mg/kg/day x2 weeks; then FLU 400 mg/day x8 weeks; then maintenance | 55/135 (note: includes 47 patients known to have died and authors also include 8 as "presumed dead" who left in earlier cohort against medical advice; acute mortality therefore estimated as 47+8=55; in second cohort, 1 patient left against medical advice not presumed dead by authors but treat as loss to follow-up so total 14-day outcomes in 135/136 patients) |  |  | first cohort 8/92 patients left hospital against medical advice (likely died); second cohort 1/44 left hospital against medical advice; second cohort of 35 patients followed after hospitalization, 8 died before starting ART, 2 lost to follow-up prior to starting, 1 HIV-negative on repeat testing; 6 died after ART initiated, and 18 known to be alive at 6 months (excluding as subset of patients from cohorts) | combined mortality in the two cohorts; note higher mortality in the earlier cohort |
| Katwere 2009 | Uganda | FLU/AmB (predominately FLU) | 72% (36/50) managed out of hospital with oral FLU (dose NS); 28% (14/50) managed in hospital with AmB (dose NS); no details on consolidation therapy or maintenance |  | 20/50 |  | NS for cryptococcal meningitis; overall 2/180 participants lost to follow-up over observation period | patients followed for 4 months; outcomes NS for 2-weeks or 10-weeks and 4-mo included here in analysis |
| Lightowler 2010 | South Africa | AmB | standard AmB 0.7 mg/kg/day x14 days; 80% (149/186) received AmB including one with FLU; 15% (28/186) received FLU 400 mg/day monotherapy primary due to renal dysfunction (25/28); 5% (9/186) received no antifungal therapy | 52/186 |  |  | In-hospital loss to follow-up NS | note that 32% (60/186) patients died within 4 weeks |
| Longley 2008 | Uganda | FLU | 30 patients received FLU 800 mg/day x2 weeks in first cohort; then 30 received FLU 1200 mg/day x2 weeks in second sequential cohort; both then FLU 400 mg/day x8 weeks; then maintenance | 17/57 | 31/57 |  | 3 participants lost to follow-up within the first 2 weeks | no difference in mortality between groups receiving FLU 800 mg/day or 1200 mg/day |
| Loyse 2012 | South Africa | AmB + 5FC | AmB 0.7-1 mg/kg/day and 5FC 100 mg/kg/day x2 weeks | 1/20 | 6/20 |  | 1 refused all interventions on day 4; considered lost to follow-up by investigators |  |
| Loyse 2012  (same study as above) | South Africa | AmB + FLU (azole) | combined: AmB 0.7-1 mg/kg/day and FLU 800 mg/day x2 weeks; AmB 0.7-1 mg/kg/day and FLU 1200 mg/day x2 weeks; or AmB 0.7-1 mg/kg/day and voriconazole 300 mg twice daily x2 weeks | 8/58 | 16/55 |  | 3 lost to follow-up between weeks 2 and 10 | combined the mortality outcomes for all azole class treatment groups (no difference in mortality between groups) |
| Makadzange 2010 | Zimbabwe | FLU | FLU 800 mg/day x10 weeks; then maintenance | 7/26 |  | 12/24 | 2 lost to follow-up between 2 weeks and 6 months | excluded group randomized to early ART as associated with very high mortality and not recommended in guidelines |
| Molloy 2018 | Malawi, Zambia, Tanzania, and Cameroon | FLU + 5FC | FLU 1200 mg/day and 5FC 100 mg/kg/day x2 weeks | 41/225 | 79/225 |  | of 678 total, 1 lost to follow-up within 2 weeks and 3 between 2 weeks and 10 weeks; unable to determine which group; only 0.6% of patients and unlikely to impact findings so assumed alive here |  |
| Molloy 2018  (same study as above) | Malawi, Zambia, Tanzania, and Cameroon | AmB (1 week) + FLU | AmB 1 mg/kg/day x1 week and FLU 1200 mg/day x2 weeks | 36/111 | 54/111 |  | see above |  |
| Molloy 2018  (same study as above) | Malawi, Zambia, Tanzania, and Cameroon | AmB + FLU | AmB 1 mg/kg/day and FLU 1200 mg/day x2 weeks | 25/114 | 47/114 |  | see above |  |
| Molloy 2018  (same study as above) | Malawi, Zambia, Tanzania, and Cameroon | AmB (1 week) + 5FC then FLU (1 wk) | AmB 1 mg/kg/day and 5FC x1 week then FLU 1200 mg/day days 8-14 | 13/113 | 27/113 |  | see above |  |
| Molloy 2018  (same study as above) | Malawi, Zambia, Tanzania, and Cameroon | AmB + 5FC | AmB 1 mg/kg/day and 5FC x2 weeks | 24/115 | 44/115 |  | see above |  |
| Muzoora 2012 | Uganda | AmB (5-day) + FLU | AmB 1 mg/kg/day for 5 days with FLU 1200 mg/day for 2 weeks then 800 mg/day until ART started | 7/30 | 8/29 |  | 1 patient lost to follow-up between 2 and 10 weeks |  |
| Nussbaum 2010 | Malawi | FLU + 5FC | FLU 1200 mg/day and 5FC 100 mg/kg/day x2 weeks | 2/21 | 9/21 |  | 0 lost to follow-up |  |
| Nussbaum 2010  (same study as above) | Malawi | FLU | FLU 1200 mg/day x2 weeks | 7/19 | 11/19 |  | 1 patient lost to follow-up at 1 day when family decided to take him home; censored from mortality analysis by investigators |  |
| Ochieng 2009 | Kenya | AmB | AmB 0.7 mg/kg/day x2 weeks | 21/70 |  |  | NS |  |
| Rhein 2019 | Uganda | AmB + FLU | standard AmB 0.7-1 mg/kg/day x7-14 days and FLU 800-1200 mg/day; followed by consolidation and maintenance FLU | 65/229 (personal communication Joshua Rhein) | 94/229 (personal communication Joshua Rhein) | 106/228 (18 weeks) | 3 lost to follow-up; personal communication with Joshua Rhein 1 at week 14, others within 2 weeks | CROI abstract reported 46% mortality for placebo arm of ASTRO-CM trial, study later published in Lancet Infect Dis |

List of abbreviations: 5FC = flucytosine; AmB = amphotericin B; ART = antiretroviral therapy; C-IRIS = cryptococcal immune-reconstitution inflammatory syndrome; CrAg = cryptococcal antigen; CSF = cerebrospinal fluid; FLU = fluconazole; GCS = Glasgow coma scale; H2O = water; IQR = interquartile range; LFT = liver function test; NS = not specified; RCT = randomized-controlled trial

**TB meningitis studies - details (all considered routine care studies):**

| First author [ref] | Year(s) | Study type | Country | Setting | Patients no. / details | Sex | Age (years) | HIV +ve | CD4 count (cells/µL) | Baseline ART status | Altered mental status | Diagnostic details | Treatment(s) | ART post-dx | Follow-up duration | Notes |
| --- | --- | --- | --- | --- | --- | --- | --- | --- | --- | --- | --- | --- | --- | --- | --- | --- |
| Bahr 2018 | 2015-2016 | validation study | Uganda | referral hospital (Mbarara) | 22 definite / HIV-infected adult (≥18 years) with suspected meningitis | 59% (13/22) male | median 32 (IQR 30-34) | 100% (22/22) | median 72 (IQR 43-124) | 59% (13/22) on ART; plus 1/22 prior ART | 77% (17/22) with GCS <15 | positive CSF XPert Ultra, Xpert, and/or MGIT culture | NS | NS | In-hospital; indicated some post-discharge follow up but no details provided | Xpert Ultra validation study |
| Bergemann 1996 | 1994-1995 | retrospective cohort 1994 / prospective cohort 1995 | South Africa | university hospital (Baragwanath) | 72 (15 definite) / adults on the medical and surgical wards with abnormal CSF examinations suggestive of meningitis | 54% (39/72) male | NS | 65% (39/60) with known HIV status | NS | not available during study period | NS | MTB cultured from CSF, TB proven at another site in patient with aseptic meningitis, or empirically on basis of neurological symptoms, typical alterations of CSF, exclusion of other etiologies, and positive response to anti-tuberculous therapy | NS | not available | In-hospital | for TBM, only 39% (28/72) with culture-proven disease, including 21% (15/72) CSF and 18% (13/72) other sites |
| Hakim 2000 | 1994 | prospective cohort | Zimbabwe | 2 referral hospitals (Harare Central and Parirenyatwa) | 21 definite TBM with HIV infection (24 overall including 3 seronegative patients for whom clinical outcomes not provided) / included consecutive patients admitted by medical firms with clinical suspicion of meningitis | 81% (17/21) male | median 30 (range 23-48) | 100% (21/21) | median 131 (range 0-750); CD4 available for 14/21 | not available during study period | 76% (13/17) of confirmed TBM with GCS<15 of those with recorded GCS | positive CSF Ziehl-Neelsen stain and/or Lowenstein-Jensen culture | NS | not available | In-hospital | excluded outcomes in 3 patients who were not HIV-infected |
| Karstaedt 1998 | 1994-1997 | retrospective cohort | South Africa | university hospital (Chris Hani Baragwanath) | 56 definite among adults aged ≥18 years / Adults (≥18 years) with microbiologically confirmed TBM | slight majority male | all ≥18 years; 11% 6/56) ≥60 years of age | 78% (39/50) among aged 18-59 | median 128 (range 36-464); CD4 available data for 21/39 | Not available | 75% (42/56) with altered mentation | positive CSF AFB smear and/or Lowenstein-Jensen culture; all 56 culture positive, 3also positive on smear | standard ATT for most; 11% (6/56) received corticosteroids; 18% (10/56) received no therapy because they died (7) or  discharged (3) before diagnosis | not available | In-hospital |  |
| Luma 2013 | 2004-2009 | retrospective cohort | Cameroon | referral hospital (Douala General) | 54 (only 1 definite) / HIV-infected adults (>18 years) admitted to internal medicine service and treated for TBM | 56% (30/54) male | mean 40 (SD 13) | 100% (54/54) | median 16 (IQR 10-34) | NS; note few patients on ART during study period | NS | decision to treat based on combination of clinical, radiological and biochemical argument or persistent or deteriorating clinical state during treatment for bacterial meningitis | standard ATT | not generally available | In-hospital | only 1/54 patients microbiologically diagnosed with TBM; many with suggestive clinical features, e.g. 44% 24/54 suspicion of TB on chest X-ray, majority had abnormal CT head imaging findings |
| Majwala 2013 | 2010 | prospective cohort | Uganda | referral hospital (Mbarara) | 32 definite / adults (≥18 years) admitted to medical ward and clinically diagnosed with meningitis | 56% (18/32) male | mean 40 (SD 16) | 81% (25/31) with known status | NS for TBM cases | NS for TBM cases | mean GCS 13 (SD 2) | positive CSF PCR | NS | NS | 30 days |  |
| Ogun 2005 | 1992-2002 | retrospective cohort | Nigeria | referral hospital (Olabisi Onabanjo) | 40 (none appear definite but most with evidence of TB elsewhere) / HIV-infected patients evaluated for neurological manifestations of HIV/AIDS | NS for TBM; 56% (86/154) males in overall cohort | NS for TBM; mean 32 (SD 3.6) in overall cohort | 100% (40/40) | Not tested | not generally available during study | NS | diagnostic criteria included: features of subacute / chronic meningitis with multiple cranial nerve lesions; with or without laboratory / radiological evidence of TB and/or positive CSF culture for AFB and / or a xanthochromatic CSF; and / or significant mantoux reaction or anergy; and / or elevated ESR | standard ATT with prednisolone for the first 6 weeks | not generally available | followed through end of therapy | most patients diagnosed with TB meningitis had supporting evidence; 32/40 had prior pulmonary disease, 5/40 abdominal TB; CSF xanthochromatic in 37/40; all had Mantoux skin reaction >15 mm |
| Raberahona 2017 | 2007-2014 | retrospective cohort | Madagascar | referral hospital (Joseph Raseta Befelatanana) | 75 (8 definite , 44 probable, and 23 possible TBM) / adult patients (≥16 years) with diagnosis of TBM admitted to hospital over study period | 56% (42/75) male | mean 35.4 (SD 12.7) | 4% (3/75) HIV infected (all screened) | NS | NS | 73% (55/75) with altered mental status | used expert case definition (Marais *et al.* Lancet Infect Dis 2011) | 70/75 received rifampicin, isoniazid, ethambutol, and pyrazinamide; 5/75 patients who had prior TB history received rifampicin, isoniazid, ethambutol, pyrazinamide, with additional streptomycin | NS | In-hospital | among 75 patients, only 13 had a microbiological examination for TB (8 of which positive for AFB, 3 of which positive on culture); used established case definition from Marais *et al.* from Lancet Infect Dis. |
| Schutte 2001 | 1994-1998 | prospective cohort | South Africa | referral hospital (Pretoria Academic) | 40 (several noted to be confirmed on post-mortem evaluation) / consecutive adult patients admitted to hospital with diagnosis of definite or probable TB meningitis | NS | NS | 54% (20/37) who had testing; 3 died before tested | mean 180 (range 7-473) | not available | mean GCS 13 (range 3-15) | included definite cases with CSF TB PCR or culture positive or stain or post-mortem evidence; patients with TB at another site with characteristic clinical and CSF findings or known history of previous TB who improved on TB therapy coupled with characteristic clinical and CSF findings or showed improvement on TB therapy with characteristic clinical and CSF findings classified as highly probable TB meningitis | NS | not available | NS (appear in-hospital) | TB confirmed on several post-mortem evaluations for patients who died |
| Siddiqi 2014 | 2010-2012 | prospective cohort | Zambia | referral hospital (University Teaching Hospital in Lusaka) | 48 definite / consecutive HIV-infected adults who presented to emergency department with symptoms suggestive of CNS infection requiring a lumbar puncture | NS for TBM | NS for TBM | 100% (48/48) | NS for TBM cases | NS for TBM cases | NS | positive CSF MTB PCR | NS | NS | In-hospital |  |
| Siddiqi 2019 | 2014-2017 | Prospective cohort | Zambia | referral hospital (University Teaching Hospital in Lusaka) | 550 patients with definite (19% [107] CSF culture-confirmed) or probable TBM (probable TBM according to uniform case definition from Marais et al.) | 53% (284/540) male among those with known HIV status | Median 35 (IQR 30-41) for HIV-positive and 25 (IQR 24-27) HIV-negative | 86% (474/550) known HIV-infected | median 104 (IQR 45-167) HIV-positive culture-positive TBM, 129 (IQR 42-346) HIV-positive culture-negative TBM | 56% (264/475) on ART overall for culture-positive and culture-negative cases | 33% (32/97) GCS ≤10 in HIV-positive culture-positive cases, 25% (2/8) in HIV-negative culture-positive cases | Included definite cases that were CSF MGIT culture positive and probable cases according to a published uniform cases definition | NS | NS | In-hospital | Investigators reported 1-year mortality but 1-year outcomes unknown for >20% of participants so excluded here |
| Tenforde 2019 | 2004-2015 | retrospective cohort | Botswana | national audit including hospitals throughout Botswana | 48 definite / included cases with positive CSF TB culture or AFB smear | 52% (24/46) male | median 34 (IQR 24-45) | 76% (22/29) with HIV status details | median 105 (IQR 34-162) | unknown | NS | positive CSF AFB smear and/or culture | NS | NS | up to 1 year |  |

List of abbreviations: AFB = acid-fast bacillus; ART = antiretroviral therapy; ATT = anti-tuberculous therapy; CSF = cerebrospinal fluid; CT = computed tomography; ESR = erythrocyte sedimentation rate; GCS = Glasgow coma scale; IQR = interquartile range; MGIT = Mycobacteria growth indicator tube; MTB = *Mycobacterium tuberculosis*; NS = not specified; PCR = polymerase chain reaction; RCT = randomized-controlled trial; SD = standard deviation; TBM = tuberculous meningitis

**TB meningitis studies - outcomes (all considered routine care studies):**

| First author | Country | Treatment | Number overall TBM diagnoses and details | Microbiologically confirmed? | 2-wk mortality | In-hospital mortality | Mean/median length-of-stay (if in-hospital mortality) | 10-wk mortality | 6-mo mortality | Details of loss to follow-up | Notes |
| --- | --- | --- | --- | --- | --- | --- | --- | --- | --- | --- | --- |
| Bahr 2018 | Uganda | NS | 22 with microbiologically confirmed TB meningitis | yes |  | 11/22 | NS |  |  | note 2 additional patients indicated to have died after discharge (unknown time) and 1 lost to follow-up after discharge | Total known mortality 13/22 with additional patient lost to follow-up after discharge |
| Bergemann 1996 | South Africa | NS | 72 diagnoses made overall but only 39% (28/72) with culture-confirmed disease including only 21% (15/72) cultured from CSF | mix |  | 29/72 | NS (note 2-week mortality of 20/72); mean length of stay non-bacterial meningitis 7 days (SD 7) |  |  | NS | minority had culture-confirmed disease; outcomes not disaggregated for confirmed versus unconfirmed. |
| Hakim 2000 | Zimbabwe | NS | 21 microbiologically confirmed cases | yes |  | 14/21 | NS |  |  | NS |  |
| Karstaedt 1998 | South Africa | Standard ATT | 56 microbiologically confirmed cases | yes |  | 39/56 | NS |  |  | NS |  |
| Luma 2013 | Cameroon | Standard ATT | 54 diagnosed with TBM (only 1 positive CSF AFB smear), others diagnosed based on CSF, clinical, and radiological features or failure to improve on anti-bacterial therapy | no |  | 43/54 | NS |  |  | NS | only 1 case microbiologically confirmed by AFB smear |
| Majwala 2013 | Uganda | NS | 32 microbiologically confirmed TB meningitis cases | yes |  | 9/32 | NS |  |  | NS | 30-day mortality for patients with TBM 47% (14/30) with 2 patients lost to follow-up after hospital discharge |
| Ogun 2005 | Nigeria | standard ATT with steroids first 6 weeks | 40 | mix |  |  |  |  | 31/40 | remaining 9 patients known to be alive | appears most cases without CSF confirmation; however, most had history of pulmonary or extra-pulmonary TB supporting diagnosis |
| Raberahona 2017 | Madagascar | standard ATT | 75 including mix of definite probable and possible | mix |  | 21/75 | NS |  |  | NS |  |
| Schutte 2001 | South Africa | NS | 40 | mix (16 with positive CSF culture or PCR for MTB, 8 had post-mortem findings consistent with TBM including caseating granulomas with meningeal exudates) |  | 12/40 | NS |  |  | NS |  |
| Siddiqi 2014 | Zambia | NS | 48 all microbiologically confirmed | yes |  | 22/48 | NS |  |  | NS | all microbiologically confirmed |
| Siddiqi 2019 | Zambia | NS | 550 cases with mix of culture-confirmed and culture-negative | only 19% (107/550) cases culture-confirmed |  | 141/540 (excluding 10 without known HIV-status in whom mortality not reported) | NS |  |  | See note | 1-year mortality reported in study as 59% among those who could be tracked but outcomes missing in 21% (113/550) patients; in sensitivity analysis, assuming those lost to follow-up had died 1-year mortality would be 67%. |
| Tenforde 2019 | Botswana | NS | 48 all microbiologically confirmed | yes | 18/48 |  |  | 22/48 | 27/48 | NS; a proportion of deaths may not have been captured in national death registry | 1-year rather than 6-month mortality provided |

List of abbreviations: AFB = acid-fast bacillus; ART = antiretroviral therapy; ATT = anti-tuberculous therapy; CSF = cerebrospinal fluid; CT = computed tomography; ESR = erythrocyte sedimentation rate; GCS = Glasgow coma scale; IQR = interquartile range; MGIT = Mycobacteria growth indicator tube; MTB = *Mycobacterium tuberculosis*; NS = not specified; PCR = polymerase chain reaction; RCT = randomized-controlled trial; SD = standard deviation; TBM = tuberculous meningitis

**Pneumococcal meningitis studies - details (considered routine care studies, except clinical trials as indicated by *):**

| First author [ref] | Year(s) | Study type | Country | Setting | Patients no. / details | Sex | Age (years) | HIV +ve | CD4 count (cells/µL) | Baseline ART status | Altered mental status | Diagnostic details | Treatment(s) | ART post-dx | Follow-up duration | Notes |
| --- | --- | --- | --- | --- | --- | --- | --- | --- | --- | --- | --- | --- | --- | --- | --- | --- |
| Ajdukiewicz 2011 * | 2006-2008 | RCT | Malawi | referral hospital (Queen Elizabeth) | 52 with microbiologically confirmed *Streptococcus pneumoniae*  (of 128 in group not randomized to adjunctive glycerol) / age ≥16 years with clinical suspicion of meningitis plus CSF evidence of bacterial meningitis (>100 WBCs/µL with predominant neutrophils or Gram-stain showing bacteria); exclusion criteria: age <16 years, cryptococcal meningitis, <100 WBCs/µL, pregnancy, heart failure, known type-2 diabetes, blood glucose >12 mmol/L, lymphocytic meningitis | 61/128 (48%) male overall with suspected bacterial meningitis in placebo group | median 32 (IQR 27-38) | 104/124 (84%) with HIV status | NS | 16/128 (13%) | 135/265 (51%) with GCS <14 among all participants | culture (CSF and/or blood) | ceftriaxone 2 grams twice daily for at least 10 days (have excluded patients randomized to also receive glycerol, which was associated with higher mortality prompting study termination at interim analysis) | patients found to be HIV-positive started on cotrimoxazole and referred to antiretroviral therapy clinic at discharge | 40 days | excluded patients assigned to glycerol group as associated with worse outcomes and not used for bacterial meningitis treatment based on results from this study; limited inclusion to participants with diagnostic pneumococcal meningitis; mortality in placebo group including patients without pneumococcal meningitis higher (61/125 [49%] by 40 days); note 63/380 (17%) patients meeting inclusion criteria died before recruitment, producing some selection bias and under-estimate of meningitis mortality (have included for descriptive analysis as one of 2 controlled trials for pneumococcal disease) |
| Boisier 2007 | 2003-2006 | cross-sectional audit | Niger | data from national reporting system | 303 microbiologically confirmed / cases of meningitis reported to national database and stored CSF aliquots sent to central reference laboratory for PCR testing | NS | mean age 12 years | NS | NS | NS | NS | positive CSF PCR | for those with known treatment, 65% (161/249) oily chloramphenicol, 19% (48/249) ceftriaxone, 16% (40/249) ampicillin or amoxicillin | NS | NS (likely acute) | do not give exact mortality for adults; present figure for 0-1, 2-4, 5-9, 10-14, and ≥15-year olds and state mortality did not differ between age strata with range 47% - 54%; from bar graph, mortality for ≥15-year olds ~50% |
| Cohen 2015 | 2003-2008 | prospective surveillance cohort | South Africa | GERMS-SA surveillance data | 1166 microbiologically confirmed (634 HIV-associated) / ≥15 years with Streptococcus pneumoniae cultured from sterile site | NS for meningitis; 46% (1806/3952) male overall | majority with meningitis 25-44 (66% [770/1166]) | 90% (634/707) among those with known HIV status | NS | NS | NS | positive CSF culture or positive culture from other sterile site (e.g. blood) with meningitis diagnosis reflected in medical records | NS | NS | defined as in-hospital or within 30 days of invasive pneumococcal disease diagnosis |  |
| Gordon 2002 | 1997-1999 | prospective cohort | Malawi | referral hospital (Queen Elizabeth) | 65 microbiologically confirmed / adults admitted with confirmed invasive pneumococcal disease | 45% (29/65) male | mean 32 | NS for meningitis; 95% (158/167) with invasive pneumococcal disease who consented to testing | NS | 0% (0/65) | 58% (38/65) with GCS<15 | positive CSF culture or CSF with >50 neutrophils/mL in a ppt with pneumococcemia | benzyl penicillin 2.4 g every 6 hours IV and chloramphenicol 1 g every 6 hours IV x3 weeks standard meningitis therapy | not available | up to ~1000 days (median 414 days for survivors of hospitalization) |  |
| Kleynhans 2019 | 2005-2008 and 2013-2016 (only including 2013-2016 here as prior data overlaps with Cohen 2015) | prospective surveillance cohort | South Africa | GERMS-SA surveillance data | 1145 microbiologically-confirmed pneumococcal meningitis cases 2013-2016 / in-hospital mortality outcomes for 1044 | 50% (567/1145) male | 73% (831/1139) ≥15 years of age | 70% (620/888) of those with known HIV status | NS | NS | NS | positive CSF culture, PCR, or latex agglutination with positive gram stain from CSF | NS | NS | In-hospital |  |
| Manga 2008 | 1995-2004 | retrospective cohort | Senegal | referral hospital (Fann University) | 73 microbiologically confirmed / ≥15 years admitted with confirmed pneumococcal meningitis during observation period | 67% (49/73) male | mean 44 (SD 19.5) | 12% (9/73) with known HIV infection | NS | not available | 59% (43/73) presented with "coma"; 62% (45/73) 61.6% with abnormal consciousness | positive CSF culture or antigen test | variety of therapies used: ampicillin (46.5%), gentamicin (45.6%), chloramphenicol (31.8%), cefotaxime (19.3%), ceftriaxone (19.3%) | NS | In-hospital |  |
| Mbelleso 2006 | 1998-2003 | cross-sectional audit | Central African Republic | neurology unit of hospital (l'hôpital de l'Amitié à Bangui) | 227 microbiologically confirmed / adults >15 years admitted with a syndrome of meningitis | NS for pneumococcal meningitis; male: female sex ratio 1.6:1 overall | NS for pneumococcal meningitis; mean 35 (range 15-80) overall | 52% (106/205) of those with known HIV status | NS | NS | NS | positive CSF culture | NS | NS | In-hospital | poor details in study including patient management |
| Ouattara 2007 | 2001-2003 | cross-sectional audit | Ivory Coast | referral hospital (Treichville) | 268 patients with bacterial meningitis including 69% (185/268) with confirmed pneumococcal meningitis / HIV-infected patients hospitalized with meningitis in an infectious diseases hospital unit with positive microbiological testing | NS for pneumococcal meningitis; male:female ratio 1:2 overall | NS for pneumococcal meningitis; mean 37 (range 19-65) overall | 100% (268/268) | mean 250 (range 5-450) | NS | 71% (190/268) with "coma" | isolated bacteria from CSF (185 *S. pneumoniae*, 62 *H. influenzae*, 13 *N. meningitidis*, 7 other *Streptococci*, 1 bacillus for 268 total) | amoxicillin 200 mg/day until fever resolved for 5 days | NS (likely not available) | In-hospital | 268 patients with confirmed bacterial meningitis; 69% (185/268) with confirmed pneumococcal meningitis; results not disaggregated by type but majority pneumococcal meningitis |
| Scarborough 2007 * | 2002-2005 | RCT | Malawi | referral hospital (Queen Elizabeth) | 275 patients with microbiologically confirmed pneumococcal meningitis (note analysed 272, not clear if not included for loss to follow-up or met exclusion criteria and include 274 patients in 10-day mortality) / inclusion criteria included clinical suspicion of bacterial meningitis and either positive CSF on microscopy (organisms seen on Gram's stain or >100 white cell count /mm3 with >50% neutrophils or cloudy CSF when immediate microscopy not available); exclusion criteria included age <16 years, corticosteroids in the past 48 hours, CSF India ink positive, or contraindication to any study drug | NS for pneumococcal cases; 49% (230/465) male overall | NS for pneumococcal cases; of 465 patients overall with bacterial meningitis, mean 32.3 (SD 10.1) corticosteroid group and 32.6 (SD 11.4) placebo group | NS for pneumococcal cases; 90% (389/434) of those tested overall | NS for pneumococcal cases; overall median 102 (IQR 51-169) | 0 on ART at baseline | overall mean GCS 11 (SD 4) corticosteroid group and 11 (SD 3) placebo group | pneumococcal meningitis diagnosed as positive CSF PCR or culture or blood culture in the context of CSF containing white cell count >100/mm3 with >50% neutrophils | randomized to ceftriaxone 2 g IV/IM twice daily for 10 days with adjunctive dexamethasone or placebo | patients enrolled after March 2004 who survived to follow-up referred by study team to the hospital ART clinic | 6 months | note: 9 patients died before randomization could occur |
| Tenforde 2019 | 2004-2015 | retrospective cohort | Botswana | national meningitis audit | 238 microbiologically confirmed / included cases with positive CSF culture for *Streptococcus pneumoniae* | 41% (97/238) male | median 33 (IQR 18-42) | 64% (79/123) HIV-infected among those with HIV-related data available | median 221 (IQR 141-421) | 44% (35/79) on ART among HIV-infected | NS | positive CSF culture | NS | NS | up to 1 year |  |
| Yaro 2006 | 2002-2005 (not continuous) | cross-sectional audit | Burkina Faso | variety of surveillance hospitals and health centres from 3 districts in Burkina Baso | 96 cases of confirmed Streptococcus pneumoniae meningitis in those ≥15 years of age / Local health providers prospectively identified patients with suspected meningitis; an aliquot of CSF and case report form were transported to central lab for microbiological testing | NS for pneumococcal meningitis | all patients ≥15 years | NS | NS | NS | NS | positive CSF PCR, latex agglutination, or culture | treatment regimen specified for overall population of patients with suspected meningitis; of 206 patients with known treatment regimen (with pneumococcus or other etiologies), 34% (71/206) received ceftriaxone (dose and duration NS), 31% (63/206) received 1 or 2 doses of chloramphenicol, and 12% (25/206) received 1 or 2 doses of ampicillin or amoxicillin alone; other regimens not specified | NS | NS (likely acute) | note: Traore *et al.* 2009 CID with more cases of confirmed pneumococcal meningitis from 2002-2006 but missing outcomes on >15% of patients and treatment regimens not specified; this article does not comment on missingness of outcomes data for patients |
| Wall 2017 * | 2012-2013 | prospective cohort | Malawi | referral hospital (Queen Elizabeth) | 132 patients with proven or probable bacterial meningitis, 84 confirmed *Streptococcus* *pneumoniae*  / 2 cohorts; first under routine conditions; second cohort included clinical review and delivery of parenteral ceftriaxone within 1 hour, airway support if GCS<8, and additional enhanced management; inclusion criteria >14 years with temperature >38 Celsius or <35.5 Celsius and one or more of the following: severe headache, neck stiffness, photophobia, confusion, coma or seizures | 61% (81/132) male | NS for pneumococcal meningitis; overall definite/probable bacterial meningitis phase 1 median 32 (IQR 25-42); phase 2 median 34 (IQR 28-44) | NS for pneumococcal; 73% (82/112) overall definite/probable bacterial meningitis HIV-infected among those with known status | NS for pneumococcal meningitis; overall phase 1 median 97 (IQR 41-293) ; phase 2 median 131 (IQR 76-249) | 29% (34/117) overall with definite/probable acute bacterial meningitis (does not add up to number with HIV) | NS for pneumococcal meningitis; overall median GCS 13 for phase 1 and 2; also noted that 42/132 (32%) with GCS < 11 | included patients with proven acute bacterial meningitis (positive CSF gram stain, culture, or PCR) or probable (negative microbiology but acute history and CSF pleocytosis >50 cells/µL or clumped cells with >50% neutrophils or >50% lymphocytes with prior antibiotics and biochemical evidence of meningitis, CSF: blood glucose ratio of <0.4, raised CSF protein >0.5g/L | ceftriaxone 2 grams twice daily standard treatment | NS | 40 days | sequential cohort study with phase 1 usual care and phase 2 with evaluation of a packaged intervention including rapid antibiotic therapy for suspected meningitis; mortality no better in phase 2 of study - for definite / probable acute bacterium meningitis, phase 1 mortality within 40 days 49% (28/57) versus 63% (38/60) for phase 2 among those with outcomes known |

List of abbreviations: ART = antiretroviral therapy; CSF = cerebrospinal fluid; GCS = Glasgow coma scale; IQR = interquartile range; IV = intravenous; NS = not specified; PCR = polymerase chain reaction; RCT = randomized-controlled trial; SD = standard deviation

**Pneumococcal meningitis studies - outcomes (considered routine care studies, except clinical trials as indicated by *):**

| First author | Country | Treatment | Number overall pneumo diagnoses and details | 2-wk mortality | In-hospital mortality | Mean/median length-of-stay (if in-hospital mortality) | 10-wk mortality | 6-mo mortality | Details of loss to follow-up | Notes |
| --- | --- | --- | --- | --- | --- | --- | --- | --- | --- | --- |
| Ajdukiewicz 2011 * | Malawi | ceftriaxone 2 grams twice daily for at least 10 days | 51 patients in ceftriaxone and placebo arm with microbiologically confirmed pneumococcal meningitis |  | 20/51 | reported 40-day mortality |  |  | excluded patients for mortality analysis who were lost to follow-up (loss to follow-up rare) |  |
| Boisier 2007 | Niger | for those with known treatment used, 64.7% (161/249) used oily chloramphenicol, 19% (48/249) ceftriaxone, 16% (40/249) ampicillin or amoxicillin | 303 all microbiologically confirmed |  | 151/303 | do not specify length of follow-up, likely acute mortality; relied on case reporting from facilities |  |  | NS | mix of paediatric and adult cases; included as mortality did not differ between age strata although not disaggregated by age; included as no difference in mortality between paediatric and adult cases and large number of adult cases; note reporting of treatment received and mortality outcomes depended on "goodwill" and vigilance of health care staff |
| Cohen 2015 | South Africa | NS | 1166 all microbiologically confirmed (634 in HIV positive individuals) |  | 641/1166 (336/634 HIV-positive deaths) | in-hospital or within 30 days of invasive pneumococcal disease |  |  | outcomes for all included patients |  |
| Gordon 2002 | Malawi | benzyl penicillin 2.4 g every 6 hours IV and chloramphenicol 1 g every 6 hours IV standard treatment for 3-week duration | 65 all microbiologically confirmed |  | 42/65 | in-hospital; noted median time to in-hospital death 2 days (range 1-9 days) | 45/65 | 45/65 | details on lost to follow-up not provided | 10-week and 6-month mortality derived from visual inspection of Kaplan-Meier curves; 1-year mortality appears 49/65 |
| Kleynhans 2019 | South Africa | NS | 1044 all microbiologically-confirmed |  | 450/1044 | In-hospital; 66% admitted ≥6 days |  |  |  |  |
| Manga 2008 | Senegal | variety of treatments: ampicillin (46.5%), gentamicin (45.6%), chloramphenicol (31.8%), cefotaxime (19.3%), ceftriaxone (19.3%) | 73 all microbiologically confirmed (9 HIV-positive patients) |  | 51/73 (3/9 HIV-positive deaths ) | in-hospital; noted most (48/51) deaths within first 3 days of hospitalization |  |  | NS |  |
| Mbelleso 2006 | Central African Republic | NS | 227 all microbiologically confirmed |  | 72/227 | in-hospital; mean hospital duration overall 14 days including other meningitis types |  |  | mortality outcome not available for 14.7% of cases overall which in manuscript all reported as alive- NS for cryptococcal meningitis | in-hospital mortality specifically for the 106 HIV-associated cases not specified |
| Ouattara 2007 | Ivory Coast | amoxicillin 200 mg/day until fever resolved for 5 days | 268 patients with confirmed bacterial meningitis; 69% (185/268) with confirmed pneumococcal meningitis |  | 212/268 | in-hospital; mean hospitalization 7 days (range 1-21) |  |  | NS | did not disaggregate mortality between pneumococcal and other bacterial meningitis etiologies |
| Scarborough 2007 | Malawi | ceftriaxone 2 g twice daily and dexamethasone | 130 microbiologically confirmed received corticosteroids | 10-day mortality: 61/130 | 68/129 | reported 40-day mortality |  | 74/118 (of those with known outcomes) | 0 lost to follow-up within 10 days; 1 within 40 days; 12 with unknown outcomes at 6 months | no difference in mortality between corticosteroid and placebo arms. |
| Scarborough 2007 * | Malawi | ceftriaxone 2 g twice daily and placebo | 145 microbiologically confirmed received placebo | 10-day mortality 65/144 | 72/143 | reported 40-day mortality |  | 76/127 (of those with known outcomes) | 1 lost to follow-up within 10 days; 2 within 40 day; 18 with unknown outcomes within 6 months | no difference in mortality between corticosteroid and placebo arms |
| Tenforde 2019 | Botswana | First-line ceftriaxone for acute bacterial meningitis | 238 all microbiologically confirmed | 44% (105/238) |  |  | 47% (112/238) | 49% (117/238) | NS; outcomes from national electronic death registry and may have missed a small proportion of deaths | included 1-year rather than 6-month mortality |
| Yaro 2006 | Burkina Faso | Mix of therapies for epidemic meningitis; 34% ceftriaxone, 31% chloramphenicol, 12% ampicillin or amoxicillin | 96 all microbiologically confirmed |  | 42/96 | duration follow-up NS (assume acute mortality) |  |  | NS | note: Traore 2009 from same surveillance data reports ~1/4 patients with unknown outcomes; this study seems to indicate known outcomes for all/most reported cases (mortality same in both studies at 44% with confirmed pneumococcus meningitis) |
| Wall 2017 * | Malawi | ceftriaxone 2 g twice daily | 132 with proven / probable bacterial meningitis (84/132 confirmed pneumococcal) | 59/132 (10 days) |  | Death within 10 days |  |  | followed patients for 40 days; 15 lost to follow-up during this period, include mortality 56% (66/117) in those patients with known 40-day outcomes | note: mortality data reported for proven/probable acute bacterial meningitis, of which only 63% confirmed streptococcal (62% of total proven/probable cases HIV positive) |

List of abbreviations: ART = antiretroviral therapy; CSF = cerebrospinal fluid; GCS = Glasgow coma scale; IQR = interquartile range; IV = intravenous; NS = not specified; PCR = polymerase chain reaction; RCT = randomized-controlled trial; SD = standard deviation


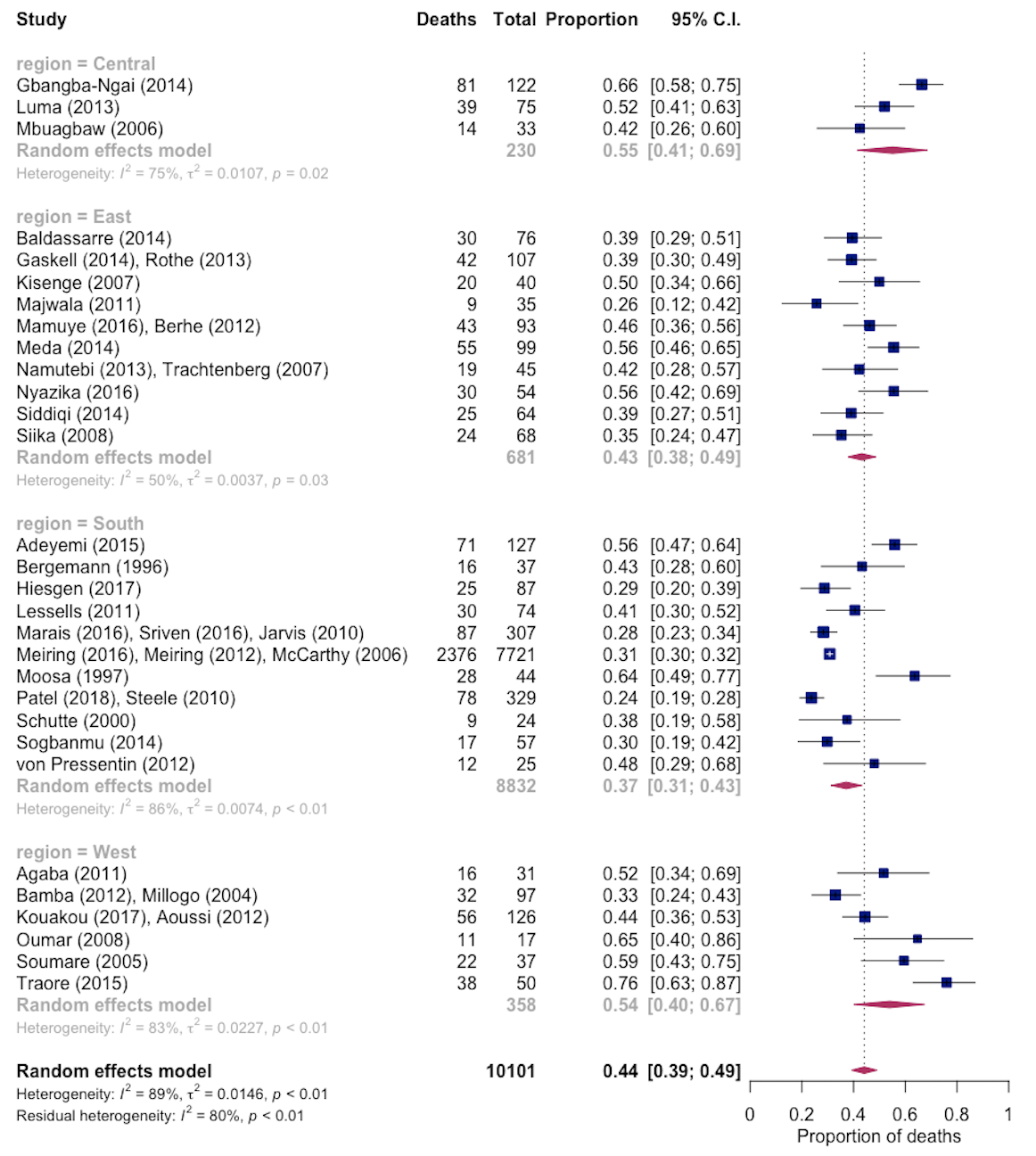


**sFigure 1.** Pooled short-term mortality of cryptococcal meningitis in routine care settings, by region


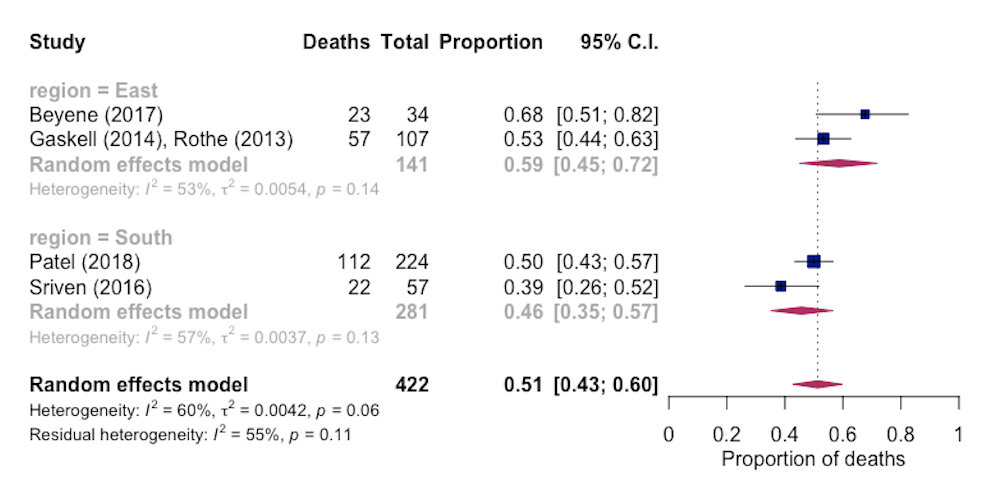


**sFigure 2.** Pooled medium-term mortality of cryptococcal meningitis in routine care settings, by region


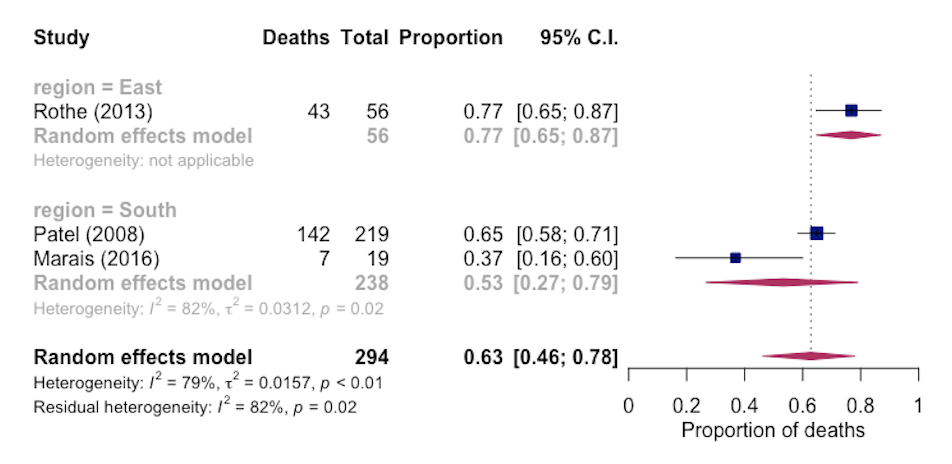


**sFigure 3.** Pooled long-term mortality of cryptococcal meningitis in routine care settings, by region

**
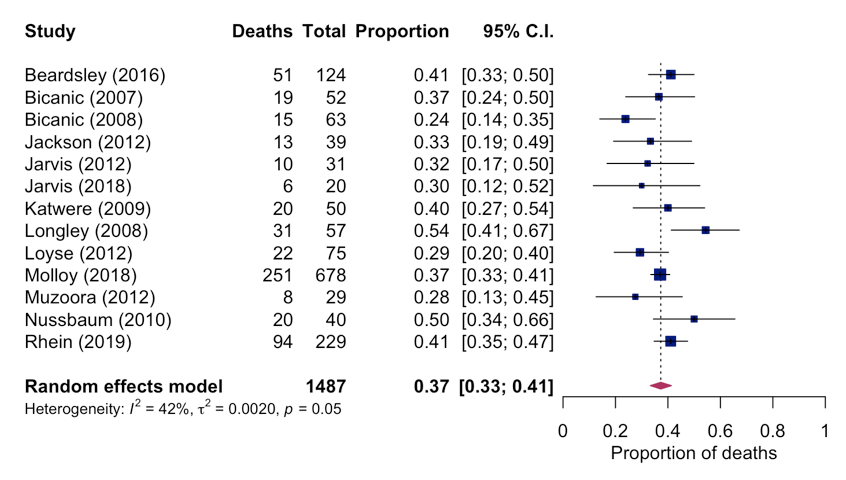
**

**sFigure 4.** Pooled medium-term mortality of cryptococcal meningitis in clinical trial settings


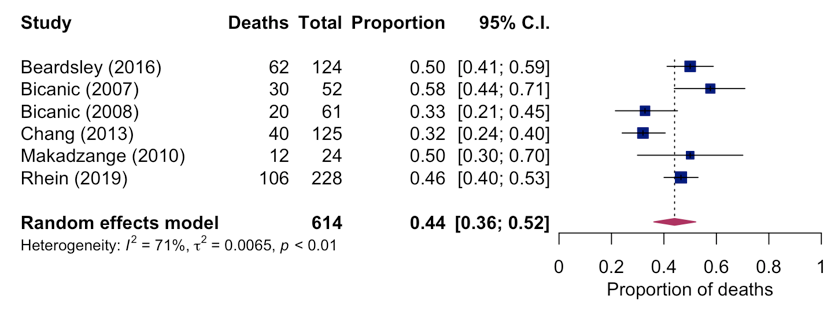


**sFigure 5.** Pooled long-term mortality of cryptococcal meningitis in clinical trial settings.

**Reasons for exclusion**

**Repeat article from included study (43 articles)**

| First author and year | Journal details | Title |
| --- | --- | --- |
| Adeyemi 2014 | African journal of primary health care & family medicine Oct 2014;6(1):E1-6 | Management of cryptococcal meningitis in a district hospital in KwaZulu-Natal: a clinical audit. |
| Bahr 2014 | Open forum infectious diseases Sep 2014;1(2):ofu070 | Standardized electrolyte supplementation and fluid management improves survival during amphotericin therapy for cryptococcal meningitis in resource-limited settings |
| Bamba 2012 | AIDS (London, England) May 2012;26(8):1039-41 | Decreasing incidence of cryptococcal meningitis in West Africa in the era of highly active antiretroviral therapy |
| Beale 2015 | PLoS neglected tropical diseases 2015;9(6):e0003847 | Genotypic Diversity Is Associated with Clinical Outcome and Phenotype in Cryptococcal Meningitis across Southern Africa |
| Bicanic 2009 | AIDS (London, England) Mar 2009;23(6):701-6 | Relationship of cerebrospinal fluid pressure, fungal burden and outcome in patients with cryptococcal meningitis undergoing serial lumbar punctures |
| Bicanic 2009 | Clinical infectious diseases : an official publication of the Infectious Diseases Society of America Sep 2009;49(5):702-9 | Independent association between rate of clearance of infection and clinical outcome of HIV-associated cryptococcal meningitis: analysis of a combined cohort of 262 patients |
| Bisson 2008 | Journal of acquired immune deficiency syndromes (1999) Oct 2008;49(2):227-9 | The use of HAART is associated with decreased risk of death during initial treatment of cryptococcal meningitis in adults in Botswana |
| Butler 2012 | PloS one 2012;7(12):e51291 | Long term 5-year survival of persons with cryptococcal meningitis or asymptomatic subclinical antigenemia in Uganda |
| Carlson 2014 | Metabolic brain disease Jun 2014;29(2):269-279 | Predictors of neurocognitive outcomes on antiretroviral therapy after cryptococcal meningitis: a prospective cohort study |
| Chang 2013 | The Journal of infectious diseases Sep 2013;208(6):898-906 | Cryptococcosis-IRIS is associated with lower cryptococcus-specific IFN-γ responses before antiretroviral therapy but not higher T-cell responses during therapy |
| Fernandes 2018 | MBio. 2018 Oct 23;9(5). pii: e02016-18. doi: 10.1128/mBio.02016-18 | Phenotypic Variability Correlates with Clinical Outcome in Cryptococcus Isolates Obtained from Botswanan HIV/AIDS Patients |
| Gordon 2003 | Malawi medical journal : the journal of Medical Association of Malawi Jun 2003;15(2):38-42 | Patient outcome in adults with pneumococcal meningitis or bacteraemia admitted to QECH |
| Jarvis 2014 | Clinical infectious diseases : an official publication of the Infectious Diseases Society of America Aug 2014;59(4):493-500 | Very low levels of 25-hydroxyvitamin D are not associated with immunologic changes or clinical outcome in South African patients with HIV-associated cryptococcal meningitis |
| Jarvis 2014 | Clinical infectious diseases : an official publication of the Infectious Diseases Society of America Mar 2014;58(5):736-45 | Determinants of mortality in a combined cohort of 501 patients with HIV-associated Cryptococcal meningitis: implications for improving outcomes |
| Jarvis 2013 | The Journal of infectious diseases Jun 2013;207(12):1817-28 | The phenotype of the Cryptococcus-specific CD4+ memory T-cell response is associated with disease severity and outcome in HIV-associated cryptococcal meningitis |
| Kadjo 2011 | Journal de Mycologie Medicale 2011;21(1):6-9 | Current aspects of neuromeningeal cryptococcosis in adults infected with HIV in the internal medicine service of the University Hospital of Treichville Abidjan (Cote d'Ivoire) |
| Kelly 2012 | The Journal of infectious diseases Jan 2012;205(1):106-10 | Epstein-barr virus coinfection in cerebrospinal fluid is associated with increased mortality in Malawian adults with bacterial meningitis |
| Kenneth 2018 | Clin Infect Dis. 2018 Sep 25. doi: 10.1093/cid/ciy817 | Symptomatic Cryptococcal Antigenemia Presenting as Early Cryptococcal Meningitis With Negative Cerebral Spinal Fluid Analysis |
| Kitonsa 2019 | PLoS One. 2019 Jan 30;14(1):e0210287. doi: 10.1371/journal.pone.0210287. eCollection 2019 | Factors affecting mortality among HIV positive patients two years after completing recommended therapy for Cryptococcal meningitis in Uganda |
| Kra 2013 | Bulletin de la Societe de pathologie exotique (1990) Feb 2013;106(1):37-42 | Clinical, biological, therapeutic and evolving profile of patients with HIV infection hospitalized at Infectious and tropical diseases unit in Abidjan (Ivory Coast) |
| Manga 2008 | Medecine tropicale : revue du Corps de sante colonial Dec 2008;68(6):625-8 | [Adult purulent meningitis caused by Streptococcus pneumoniae in Dakar, Senegal] - NOTE: overlapped with cases from Soumare 2005 but majority of observation period in 1990s, Soumare only in 2000s |
| Mdodo 2010 | East African medical journal Dec 2010;87(12):481-7 | The prevalence, clinical features, risk factors and outcome associated with cryptococcal meningitis in HIV positive patients in Kenya; NOTE: same hospitals and time period as Baldassarre |
| Meintjes 2015 | Medicine (United States) 2015;94(50): | HIV-related medical admissions to a South African district hospital remain frequent despite effective antiretroviral therapy scale-up |
| Morgan 2006 | Clinical infectious diseases : an official publication of the Infectious Diseases Society of America Oct 2006;43(8):1077-80 | Cryptococcus gattii infection: characteristics and epidemiology of cases identified in a South African province with high HIV seroprevalence, 2002-2004 |
| Naranbhai 2014 | AIDS (London, England) Mar 2014;28(5):657-66 | Compartmentalization of innate immune responses in the central nervous system during cryptococcal meningitis/HIV coinfection |
| Nyazika 2016 | The Journal of infection Jun 2016;72(6):745-752 | Cryptococcus tetragattii as a major cause of cryptococcal meningitis among HIV-infected individuals in Harare, Zimbabwe |
| Parent du Châtelet 2005 | Clinical infectious diseases : an official publication of the Infectious Diseases Society of America Jan 2005;40(1):17-25 | Bacterial meningitis in Burkina Faso: surveillance using field-based polymerase chain reaction testing |
| Raberahona 2015 | Tropical Medicine and International Health 2015;20((Raberahona M.; Rakotoarivelo R.A.; Razafinambinintsoa T.; Andrianasolo R.L.; Randria M.J.D.) Infectious Diseases Unit, University Hospital Joseph Raseta Befelatanana, Antananarivo, Madagascar):193-194 | Review of 72 cases of tuberculous meningitis in adults in Antananarivo, Madagascar |
| Rajasingham 2015 | The American journal of tropical medicine and hygiene Feb 2015;92(2):274-9 | Epidemiology of meningitis in an HIV-infected Ugandan cohort |
| Rajasingham 2014 | Emerging infectious diseases Apr 2014;20(4):722-4 | Nosocomial drug-resistant bacteremia in 2 cohorts with cryptococcal meningitis, Africa |
| Rhein 2018 | Open Forum Infect Dis. 2018 May 24;5(8):ofy122. doi: 10.1093/ofid/ofy122 | Detrimental Outcomes of Unmasking Cryptococcal Meningitis With Recent ART Initiation |
| Rolfes 2014 | Clinical infectious diseases : an official publication of the Infectious Diseases Society of America Dec 2014;59(11):1607-14 | The effect of therapeutic lumbar punctures on acute mortality from cryptococcal meningitis |
| Rolfes 2015 | Open forum infectious diseases Dec 2015;2(4):ofv157 | Cerebrospinal Fluid Culture Positivity and Clinical Outcomes After Amphotericin-Based Induction Therapy for Cryptococcal Meningitis |
| Schut 2012 | PloS one 2012;7(3):e34311 | Validation of a Dutch risk score predicting poor outcome in adults with bacterial meningitis in Vietnam and Malawi |
| Soumare 2005 | Bulletin de la Societe de pathologie exotique (1990) Jun 2005;98(2):104-7 | [Clear-fluid meningitis in HIV-infected patients in Dakar]; NOTE: instead used article from same hospital and time period and same lead author (Soumare) |
| Tugume 2017 | HIV medicine 01 2017;18(1):13-20 | Prognostic implications of baseline anaemia and changes in haemoglobin concentrations with amphotericin B therapy for cryptococcal meningitis |
| von Mollendorf 2014 | Antimicrobial agents and chemotherapy Jun 2014;58(6):3293-305 | Factors associated with ceftriaxone nonsusceptibility of Streptococcus pneumoniae: analysis of South African national surveillance data, 2003 to 2010 |
| Wajanga 2011 | Journal of the International AIDS Society Oct 2011;14():48 | Universal screening of Tanzanian HIV-infected adult inpatients with the serum cryptococcal antigen to improve diagnosis and reduce mortality: an operational study |
| Wall 2013 | PloS one 2013;8(7):e69783 | High mortality amongst adolescents and adults with bacterial meningitis in sub-Saharan Africa: an analysis of 715 cases from Malawi |
| Wall 2014 | The Journal of infection Nov 2014;69(5):440-6 | Genomic pneumococcal load and CSF cytokines are not related to outcome in Malawian adults with meningitis |
| Wall 2017 | Clinical infectious diseases : an official publication of the Infectious Diseases Society of America 02 2017;64(4):413-419 | Prediction of Outcome From Adult Bacterial Meningitis in a High-HIV-Seroprevalence, Resource-Poor Setting Using the Malawi Adult Meningitis Score (MAMS) |
| Wiesner 2012 | mBio 2012;3(5): | Cryptococcal genotype influences immunologic response and human clinical outcome after meningitis |
| Traore 2009 | Clinical Infectious Diseases 2009;48(SUPPL. 2):S181-S189  2009 | Incidence, seasonality, age distribution, and mortality of pneumococcal meningitis in Burkina Faso and Togo; NOTE: overlapping population with Yaro although larger study; included Yaro 2006 instead as Traore 2009 has missing outcomes data on >15% of patients and also has little information on treatment (mention chloramphenicol standard therapy) but Yaro 2006 shows that patients were treated using a variety of therapies) |

**<15 eligible patients (37 articles)**

| First author and year | Journal details | Title | Notes |
| --- | --- | --- | --- |
| Atangana 2003 | Cahiers Sante 2003;13(3):155-158 | Neurologic disturbancies in Human Immunodeficiency Virus carriers in Yaoundé |  |
| Bekker 2016 | PLoS ONE 2016;11(11): | Tuberculosis disease during pregnancy and treatment outcomes in HIV-infected and uninfected women at a referral Hospital in Cape Town |  |
| Bhagwan 2011 | AIDS research and treatment 2011;2011():180352 | Aetiology, clinical presentation, and outcome of meningitis in patients coinfected with human immunodeficiency virus and tuberculosis |  |
| Bisson 2013 | Clinical infectious diseases : an official publication of the Infectious Diseases Society of America Apr 2013;56(8):1165-73 | Early versus delayed antiretroviral therapy and cerebrospinal fluid fungal clearance in adults with HIV and cryptococcal meningitis | study of timing of ART following incident cryptococcal meningitis in Botswana; excluded group with ART initiated within 1 week which is not recommended as associated with higher mortality; <15 patients in delayed ART group |
| Boaz 2016 | Journal of tropical medicine 2016;2016():6573672 | Pattern, Clinical Characteristics, and Outcome of Meningitis among HIV-Infected Adults Admitted in a Tertiary Hospital in North Western Tanzania: A Cross-Sectional Study |  |
| Bottieau 2015 | Tropical Medicine and International Health 2015;20:106-107 | Clinical spectrum, main etiologies and outcome of neurological disorders in the rural Hospital of Mosango, Province of Bandundu, Democratic Republic of the Congo |  |
| Bottieau 2013 | Tropical Medicine and International Health 2013;18:197-198 | Etiology and outcome of neurological disorders in the Rural Hospital of Mosango, Province of Bandundu, Democratic Republic of Congo: Preliminary results |  |
| Ford 1994 | Journal of epidemiology and community health Jun 1994;48(3):276-80 | Bacterial meningitis in Swaziland: an 18 month prospective study of its impact |  |
| Grandgirard 2013 | Mediators of inflammation 2013;2013():312476 | The causative pathogen determines the inflammatory profile in cerebrospinal fluid and outcome in patients with bacterial meningitis |  |
| Grant 1997 | AIDS (London, England) Sep 1997;11(11):1357-64 | Profound immunosuppression across the spectrum of opportunistic disease among hospitalized HIV-infected adults in Abidjan, Côte d'Ivoire |  |
| Gudina 2016 | Tropical medicine & international health : TM & IH 07 2016;21(7):870-8 | Challenges of bacterial meningitis case management in low income settings: an experience from Ethiopia |  |
| Gudina 2016 | BMC neurology Aug 2016;16(1):153 | Adjunctive dexamethasone therapy in unconfirmed bacterial meningitis in resource limited settings: is it a risk worth taking? |  |
| Gudina 2018 | PLoS One. 2018 Jul 18;13(7):e0200067 | Outcome of patients with acute bacterial meningitis in a teaching hospital in Ethiopia: A prospective study |  |
| Harrington 2015 | Tropical Doctor 2015;45(3):164-167 | Altered mental status is an indicator of mortality and associated with both infectious and non-communicable disease in Lilongwe, Malawi |  |
| Hônnas 1998 | East African medical journal Jul 1998;75(7):396-401 | Bacterial meningitis in a rural Kenyan hospital |  |
| Hovette 1999 | Transactions of the Royal Society of Tropical Medicine and Hygiene ;93(4):368 | Cryptococcal meningitis in AIDS patients: an emerging opportunistic infection in Senegal |  |
| Koulla-Shiro 1997 | International Journal of Infectious Diseases 1997;2(1):9-11 | Etiology and outcome of adult bacterial meningitis in Yaounde, Cameroon |  |
| Kra 2012 | Medecine et sante tropicales ;22(1):75-8 | [Morbidity and mortality from infectious diseases at the Military Hospital of Abidjan, Côte d'Ivoire] |  |
| Longley 2016 | Clinical infectious diseases : an official publication of the Infectious Diseases Society of America Mar 2016;62(5):581-587 | Cryptococcal Antigen Screening in Patients Initiating ART in South Africa: A Prospective Cohort Study |  |
| Mihret 2016 | Emerging infectious diseases Jan 2016;22(1):75-8 | Surveillance of Bacterial Meningitis, Ethiopia, 2012-2013 |  |
| Mihret 2014 | Ethiopian medical journal Jan 2014;Suppl 1():43-8 | Chronic meningitis in immunocompromised adult Ethiopians visiting Tikur Anbessa Teaching Hospital and Ye'huleshet Clinic from 2003-2004 |  |
| Minta 2011 | Medecine tropicale : revue du Corps de sante colonial Dec 2011;71(6):591-5 | [Neuromeningeal cryptococcosis in Mali] |  |
| Mukendi 2017 | The American journal of tropical medicine and hygiene Nov 2017;97(5):1454-1460 | Clinical Spectrum, Etiology, and Outcome of Neurological Disorders in the Rural Hospital of Mosango, the Democratic Republic of Congo |  |
| Murphy 2010 | The international journal of tuberculosis and lung disease : the official journal of the International Union against Tuberculosis and Lung Disease Jul 2010;14(7):903-8 | Low uptake of antiretroviral therapy after admission with human immunodeficiency virus and tuberculosis in KwaZulu-Natal, South Africa |  |
| Nwosu 2001 | East African medical journal Feb 2001;78(2):97-101 | Central nervous system infections in the rainforest zone of Nigeria |  |
| Ole-Nguyaine 2004 | Annals of tropical medicine and parasitology Mar 2004;98(2):171-9 | HIV-associated morbidity, mortality and diagnostic testing opportunities among inpatients at a referral hospital in northern Tanzania |  |
| Opintan 2017 | Transactions of the Royal Society of Tropical Medicine and Hygiene Oct 2017;111(10):464-471 | High rates of cerebral toxoplasmosis in HIV patients presenting with meningitis in Accra, Ghana |  |
| Ouédraogo 2012 | Medecine et sante tropicales ;22(4):412-6 | [Acute bacterial meningitis with soluble antigen detected by latex particle agglutination tests at the Sourô-Sanou University Hospital of Bobo-Dioulasso (Burkina Faso)] |  |
| Ramos 2010 | BMC public health Apr 2010;10():215 | Childhood and adult tuberculosis in a rural hospital in Southeast Ethiopia: a ten-year retrospective study. |  |
| Razanamparany 2002 | Journal of Clinical Microbiology 2002;40(11):3964-3969 | Extrapulmonary and pulmonary tuberculosis in Antananarivo (Madagascar): High clustering rate in female patients |  |
| Siddiqi 2015 | Neurology 2015;84((Siddiqi O.; Elafros M.; Sikazwe I.; Bositis C.; Koralnik I.; Potchen M.; Theodore W.; Kalungwana L.; Birbeck G.)): | Etiologies of new onset seizure in HIV-infected Zambian adults |  |
| Silber 1999 | Journal of the neurological sciences Jan 1999;162(1):20-6 | Meningitis in a community with a high prevalence of tuberculosis and HIV infection |  |
| Siraji 2009 | HIV Medicine 2009;10:41 | Causes and outcomes of hospitalization of HIV patients in first 6 months of antiretroviral therapy (ART) in a teaching hospital in Southwestern Uganda |  |
| Sow 1998 | Medecine et Maladies Infectieuses 1998;28(6-7):511-515 | Cryptococcal meningitis and HIV infection in Dakar |  |
| Szabo 2013 | Neuro endocrinology letters Sep 2013;34(Suppl 1):28-31 | Neurologic complications and sequellae of infectious diseases in Uganda and Kenya: Analysis of 288 cases from two rural hospitals |  |
| Thinyane 2013 | Internet Journal of Infectious Diseases 2013;11(1): | Clinical profiles of HIV-infected, HAART-naive patients admitted to a tertiary level hospital in Maseru, Lesotho |  |
| Wright 1994 | South African medical journal 1994;84(3):178 | Bacterial and tuberculous meningitis in Swaziland | Per Ford 1994 above; letter also included 15 diagnosed cases of TB meningitis with unclear diagnostic criteria and likely some paediatric cases so exclude for number of reasons |

**No mortality outcomes provided (22 articles)**

| First author and year | Journal details | Title |
| --- | --- | --- |
| Amuron 2011 | AIDS research and therapy Oct 2011;8():39 | Mortality in an antiretroviral therapy programme in Jinja, south-east Uganda: a prospective cohort study |
| Benca 2007 | Neuroendocrinology Letters 2007;28(SUPPL. 2):49-50 | Ten years experience with 497 cases of neuroinfections in tropic: In limited laboratory infrastructure initially treat both, cerebral malaria and meningitis |
| Collett 2007 | South African medical journal Mar 2007;97(3):175-6 | Fluconazole donation and outcomes assessment in cryptococcal meningitis |
| Hooker 2003 | The international journal of tuberculosis and lung disease : the official journal of the International Union against Tuberculosis and Lung Disease Aug 2003;7(8):787-96 | Diagnostic utility of cerebrospinal fluid studies in patients with clinically suspected tuberculous meningitis |
| Ige 2005 | African journal of medicine and medical sciences Dec 2005;34(4):329-33 | Pattern of presentation of tuberculosis and the hospital prevalence of tuberculosis and HIV co-infection in University College Hospital, Ibadan: a review of five years (1998 - 2002) |
| Kaburi 2017 | The Pan African medical journal 2017;27():164 | Evaluation of bacterial meningitis surveillance data of the northern region, Ghana, 2010-2015 |
| Kammalac Ngouana 2015 | Journal de mycologie medicale Mar 2015;25(1):11-6 | Cryptococcal meningitis in Yaoundé (Cameroon) HIV infected patients: Diagnosis, frequency and Cryptococcus neoformans isolates susceptibility study to fluconazole |
| Ky-Ba 2016 | African Journal of Clinical and Experimental Microbiology 2016;17(1):10-17 | Dynamics of germs responsible for acute bacterial meningitis in Burkina faso in the last ten years (2005-2014) |
| Matee 2001 | East African medical journal Sep 2001;78(9):458-60 | Pathogenic isolates in meningitis patients in Dar Es Salaam, Tanzania |
| Matubu 2015 | The Central African journal of medicine ;61(1-4):5-11 | Etiology and risk factors of meningitis in patients admitted at a Central Hospital in Harare |
| Mgori 2015 | African journal of primary health care & family medicine 2015;7(1):e1-e7 | HIV and/or AIDS-related deaths and modifiable risk factors: A descriptive study of medical admissions at Oshakati Intermediate Hospital in Northern Namibia |
| Moïsi 2009 | Clinical infectious diseases : an official publication of the Infectious Diseases Society of America Mar 2009;48 Suppl 2():S49-56 | Enhanced diagnosis of pneumococcal meningitis with use of the Binax NOW immunochromatographic test of Streptococcus pneumoniae antigen: a multisite study |
| Nuoh 2016 | The Pan African medical journal 2016;25(Suppl 1):9 | Review of meningitis surveillance data, upper West Region, Ghana 2009-2013 |
| Owusu 2012 | Annals of clinical microbiology and antimicrobials Oct 2012;11():28 | Aetiological agents of cerebrospinal meningitis: a retrospective study from a teaching hospital in Ghana |
| Oyella 2012 | Journal of the International AIDS Society Mar 2012;15(1):15 | Prevalence and factors associated with cryptococcal antigenemia among severely immunosuppressed HIV-infected adults in Uganda: a cross-sectional study |
| Patel 2010 | PloS one Dec 2010;5(12):e15664 | Comparison of a clinical prediction rule and a LAM antigen-detection assay for the rapid diagnosis of TBM in a high HIV prevalence setting. |
| Patel 2013 | PLoS medicine Oct 2013;10(10):e1001536 | Diagnostic accuracy of quantitative PCR (Xpert MTB/RIF) for tuberculous meningitis in a high burden setting: a prospective study |
| Seipone 2018 | PloS one 2018;13(2):e0192060 | Tuberculous meningitis is associated with higher cerebrospinal HIV-1 viral loads compared to other HIV-1-associated meningitides |
| Storz 2016 | Journal of the neurological sciences Jul 2016;366():52-58 | Clinical findings and management of patients with meningitis with an emphasis on Haemophilus influenzae meningitis in rural Tanzania |
| Taelman 1992 | The New England journal of medicine Oct 1992;327(16):1171; author reply 1171-2 | Tuberculous meningitis in patients infected with the human immunodeficiency virus |
| Tegene 2015 | Biomedical Research and Therapy 2015;2(5): | Bacterial Meningitis: a five-year retrospective study among patients who had attended at University of Gondar Teaching Hospital, Northwest Ethiopia |
| Wall 2014 | Clinical infectious diseases : an official publication of the Infectious Diseases Society of America May 2014;58(10):e137-45 | Bacterial meningitis in Malawian adults, adolescents, and children during the era of antiretroviral scale-up and Haemophilus influenzae type b vaccination, 2000-2012 |

**Abstract only (e.g. conference proceeding) [14 articles]**

| First author and year | Journal details | Title |
| --- | --- | --- |
| Adakun 2014 | American Journal of Tropical Medicine and Hygiene 2014;91(5):568 | Validation of a tuberculous meningitis case definition in Mbarara regional referral hospital, Uganda |
| Adam 1990 | Advances in neurology: proceedings of the XIVth World Congress of Neurology. ICS883 1990:203-207 | Neuro-AIDS in a large teaching hospital in Africa |
| Adeyemi 2014 | Journal of the International AIDS Society 2014;17(4 Suppl 3):19623 | Profile and mortality outcome of patients admitted with cryptococcal meningitis to an urban district hospital in KwaZulu-Natal, South Africa |
| Andrew 2017 | Topics in Antiviral Medicine 2017;25(1):317s-318s | Sertraline and high-dose fluconazole treatment of cryptococcal meningitis in Tanzania |
| Awando 2014 | American Journal of Tropical Medicine and Hygiene 2014;91(5):316-317 | Clinical characteristics and etiologies of acute central nervous system infections in children and adults admitted to rural and urban hospitals in Kenya: Preliminary finding, 2011-2014 |
| Beale 2014 | Mycoses 2014;57:61 | Cryptococcus neoformans molecular type VNB is associated with mortality in HIV associated cryptococcal meningitis in South Africa |
| Byakika-Kibwika 2010 | Journal of the International Association of Physicians in AIDS Care 2010;9(1):64-65 | Outcomes of newly diagnosed HIV-positive patients admitted with acute opportunistic infections in Mulago National Referral Hospital, Uganda |
| Evans 2018 | Topics in Antiviral Medicine 2018;26:351s | Etiologies of symptomatic recurrence of HIV-associated cryptococcal meningitis |
| Idris 2017 | European Journal of Neurology 2017;24:40 | An experience from Sudan with tuberculosis of central nervous system: An extensive study of clinical and radiological features, treatment outcomes and predictors of mortality in 60 patients |
| Jarvis 2014 | Mycoses 2014;57:24 | HIV-associated cryptococcal meningitis: Host immunity and immunotherapy |
| Jarvis 2014 | International Journal of Infectious Diseases 2014;21  :284 | Vitamin D deficiency in HIV-infected South Africans: Common, and not associated with susceptibility, immune response, or outcome in HIV-associated cryptococcal meningitis |
| Larsen 2014 | Mycoses 2014;57:8 | High dose fluconazole therapy for cryptococcal meningitis |
| McDonald 2014 | Mycoses 2014;57:74-75 | Clinical parameters of cryptococcosis are associated with Cryptococcus strain genotype |
| Saulters 2015 | American Journal of Tropical Medicine and Hygiene 2015;93(4):321 | A clinical prediction model of in-hospital mortality derived from patients admitted with acute infection to a regional referral hospital in Mbarara, Uganda |

**Non-representative sampling (13 articles)**

| First author and year | Journal details | Title | Reason |
| --- | --- | --- | --- |
| Asselman 2010 | AIDS (London, England) Nov 2010;24(18):2871-6 | Central nervous system disorders after starting antiretroviral therapy in South Africa. | study of central nervous system disease in first year on antiretroviral therapy in South Africa with large proportion of patients with paradoxical immune-reconstitution inflammatory syndrome from prior TB meningitis or cryptococcal meningitis |
| Bicanic 2006 | Clinical Infectious Diseases 2006;43(8):1069-1073 | Symptomatic relapse of HIV-associated cryptococcal meningitis after initial fluconazole monotherapy: The role of fluconazole resistance and immune reconstitution | exclusively relapse cases; not representative of general cryptococcal meningitis cases |
| Boulware 2014 | The New England journal of medicine Jun 2014;370(26):2487-98 | Timing of antiretroviral therapy after diagnosis of cryptococcal meningitis | study of timing of ART after diagnosis of incident cryptococcal meningitis; enrolled patients after 1 week with >10% mortality within first week before enrolment, so large under-estimation of mortality |
| Burton 2011 | Transactions of the Royal Society of Tropical Medicine and Hygiene Dec 2011;105(12):675-82 | Factors associated with mortality and default among patients with tuberculosis attending a teaching hospital clinic in Accra, Ghana | clinic-based study of TB cases with likely selection bias; unclear details of TB meningitis diagnosis in this clinic-based study, but patients may have survived hospitalization / early treatment to be registered in clinic and included |
| Jarvis 2010 | South African medical journal = Suid-Afrikaanse tydskrif vir geneeskunde Jun 2010;100(6):378-82 | Symptomatic relapse of HIV-associated cryptococcal meningitis in South Africa: the role of inadequate secondary prophylaxis | subset of data from Jarvis *et al.* 2010 (J Infect) prospective cohort of patients who had relapsed cryptococcal meningitis; not representative of population developing cryptococcal meningitis |
| Jowi 2007 | East African medical journal Feb 2007;84(2):67-76 | Clinical and laboratory characteristics of hospitalised patients with neurological manifestations of HIV/AIDS at the Nairobi hospital | study from single private hospital in Nairobi; likely not representative of patients treated for cryptococcal meningitis and TB meningitis (selection bias) and excluded (very low in-hospital mortality of 1/33 with cryptococcal meningitis and 0/16 for TB meningitis) |
| Marais 2016 | AIDS 2016;30(3):395-404 | Interleukin-17 mediated differences in the pathogenesis of HIV-1-associated tuberculous and cryptococcal meningitis | very strict exclusion criteria and population with mortality outcomes not representative of general TB meningitis population; cases ineligible if contraindication to lumbar puncture or severe TB meningitis (modified British Medical Research Council grade III disease severity), also excluded 10 from analysis (10/44 initial) due to reported poor adherence to antiretroviral or antituberculous treatment, default from study, 1 of 3 study lumbar punctures not performed, dual infection with another organism, death prior to antiretroviral therapy initiation |
| Marais 2013 | Clinical infectious diseases : an official publication of the Infectious Diseases Society of America Feb 2013;56(3):450-60 | Frequency, severity, and prediction of tuberculous meningitis immune reconstitution inflammatory syndrome | same cohort as Marais *et al.* 2016 (AIDS) |
| Nalintya 2018 | J Acquir Immune Defic Syndr. 2018 Jun 1;78(2):231-238. doi: 10.1097/QAI.0000000000001669 | A Prospective Evaluation of a Multisite Cryptococcal Screening and Treatment Program in HIV Clinics in Uganda | prospective cryptococcal antigen (CrAg) screening program that diagnosed 17 CrAg-positive patients with cryptococcal meningitis by lumbar puncture; large number of symptomatic patients with presumptive cryptococcal meningitis (7 cases) that declined lumbar puncture, all of whom died (6) or were lost to follow-up (1), likely due to undiagnosed cryptococcal meningitis in some/all cases |
| Ousley 2018 | Clinical Infectious Diseases 2018;66((Maman D.) Epicentre, Paris, France):S126-S132 | High Proportions of Patients with Advanced HIV Are Antiretroviral Therapy Experienced: Hospitalization Outcomes from 2 Sub-Saharan African Sites | cohort from 2 hospitals in Democratic Republic of Congo and Kenya managed by an international non-governmental organization (NGO), Médecins sans Frontières; excluded for a couple of reasons, including mention of systematic hospital cryptococcal antigen (CrAg) screening of blood possibly recognizing early central nervous system infection, also likely non-representative management with international NGO, MSF also known to use flucytosine (treatment regimen not specified) |
| Patel 2008 | South African Medical Journal 2008;98(8):610-613 | Temporal evolution of cerebrospinal fluid following initiation of treatment for tuberculous meningitis | likely non-representative sampling and risk of immortality bias of TB meningitis cases in South Africa; excluded patients whose CSF change from serial lumbar punctures was not keeping with TB meningitis, had incomplete information, or treated with any antibiotics other than anti-tuberculous therapy; very few cases definite tuberculous meningitis (5/99) with most probable TB meningitis |
| Sunpath 2012 | The international journal of tuberculosis and lung disease : the official journal of the International Union against Tuberculosis and Lung Disease Jul 2012;16(7):917-23 | Operationalizing early antiretroviral therapy in HIV-infected in-patients with opportunistic infections including tuberculosis | retrospective cohort study evaluating 6-month outcomes of participants in South Africa with hospital admission for an opportunistic infection who started early ART while in-hospital; high risk of selection bias as would have excluded patients who died before they could initiate ART (reported 6-month mortality of 20% [8/40] among cryptococcal meningitis cases) |
| Wake 2018 | Clin Infect Dis. 2018 Feb 10;66(5):686-692 | High Cryptococcal Antigen Titers in Blood Are Predictive of Subclinical Cryptococcal Meningitis Among Human Immunodeficiency Virus-Infected Patients | results from cryptococcal antigen screening cohort in South Africa; a large proportion / majority of patients diagnosed with cryptococcal meningitis were asymptomatic with positive cryptococcal antigen screening in blood; low overall mortality (22%) at 6-months, perhaps representing early asymptomatic meningitis with better prognosis |

**Inadequate diagnostic details or mortality outcomes not disaggregated (13 - 2 included for other meningitis aetiology = 11 articles)**

| First author and year | Journal details | Title | Reason |
| --- | --- | --- | --- |
| Békondi 2006 | International journal of infectious diseases : IJID : official publication of the International Society for Infectious Diseases Sep 2006;10(5):387-95 | Primary and opportunistic pathogens associated with meningitis in adults in Bangui, Central African Republic, in relation to human immunodeficiency virus serostatus. | Central African Republic study with cryptococcal and pneumococcal meningitis cases; for cryptococcal meningitis, 85 cases identified but included in-hospital outcomes for only 30 (reported as 100%); for pneumococcal meningitis, 26 microbiologically-confirmed cases among 103 purulent meningitis cases, did not state mortality for pneumococcal meningitis |
| Bergemann 1996 | QJM : monthly journal of the Association of Physicians Jul 1996;89(7):499-504 | The spectrum of meningitis in a population with high prevalence of HIV disease | included for cryptococcal meningitis but excluded for pneumococcal meningitis; 28 cases of microbiologically-confirmed pneumococcal meningitis; minority of cases with confirmed *S. pneumoniae* (36% of all acute bacterial meningitis) and did not state mortality for pneumococcal cases |
| Berhe 2012 | AIDS research and therapy Apr 2012;9():11 | The pattern and predictors of mortality of HIV/AIDS patients with neurologic manifestation in Ethiopia: a retrospective study | included for cryptococcal meningitis but excluded for TB and pneumococcal meningitis; 78 cases of TB meningitis based on some or all of the following: i) headache, fever, neck rigidity, change in mentation ii) cerebrospinal fluid (CSF) lymphocytosis iii) Acid-fast bacilli (AFB) on CSF smear iv) evidence of active tuberculosis in other part of the body (miliary tuberculosis, pulmonary tuberculosis etc.) v) CT/MRI showing meningeal enhancement vi) response to tuberculosis treatment; only 1/78 cases positive for CSF AFB smear and proportion of cases that met each of the criteria not detailed; excluded for pneumococcal meningitis because reported outcomes for bacterial meningitis but did not state mortality for pneumococcal cases |
| Boumandouki 1993 | Bulletin de la Societe de pathologie exotique (1990) 1993;86(2):141-3 | [Clinical, epidemiological and therapeutic aspects of purulent meningitis in the adult. Apropos of 74 cases treated at CHU of Brazzaville (Congo)] | gave overall mortality for purulent meningitis (with 32% [24/74] confirmed pneumococcal), did not state mortality for pneumococcal meningitis cases |
| Chapp-Jumbo 2006 | African health sciences Mar 2006;6(1):55-8 | Neurologic infections in a Nigerian university teaching hospital | TB meningitis study with unclear criteria for diagnosis other than clinical history and "CSF findings;" states lumbar puncture performed in 10/19 patients with given diagnosis of TB meningitis |
| Elliott 1995 | Transactions of the Royal Society of Tropical Medicine and Hygiene 1995;89(1):78-82 | The impact of human immunodeficiency virus on mortality of patients treated for tuberculosis in a cohort study in Zambia | study from Zambia that included large number of extra-pulmonary TB cases (126 extrapulmonary or combined pulmonary and extrapulmonary) but did not state location of extrapulmonary disease including if meningitis |
| French 2002 | AIDS (London, England) May 2002;16(7):1031-8 | Cryptococcal infection in a cohort of HIV-1-infected Ugandan adults | most patients did not have confirmed cryptococcal meningitis; defined cryptococcal meningitis as positive blood cryptococcal antigen with fever with absence of other explanation or positive preterminal blood cryptococcal antigen in a patient who died and without confirmatory clinical information |
| Gordon 2000 | Clinical infectious diseases : an official publication of the Infectious Diseases Society of America Jul 2000;31(1):53-7 | Bacterial meningitis in Malawian adults: pneumococcal disease is common, severe, and seasonal | multiple reasons; pneumococcal meningitis cases in Gordon *et al.* 2002 (included); cryptococcal meningitis cases did not have treatment outcomes and patients discharged home with analgesia after diagnosis as treatment not available during period of study; 44 patients given provisional diagnosis of TB meningitis based on CSF WCC >100, >65% lymphocytes and no other pathogen identified; no patients diagnosed microbiologically or based on combination of CSF and other supportive clinical findings |
| Kendi 2013 | Postgraduate medical journal Feb 2013;89(1048):73-7 | Predictors of outcome in routine care for Cryptococcal meningitis in Western Kenya: lessons for HIV outpatient care in resource-limited settings | cohort of patients who received serum cryptococcal antigen testing in outpatient settings; defined cryptococcal meningitis as positive serum cryptococcal antigen with at least 1 symptom of meningitis without lumbar puncture confirmation; low case-fatality reported and most patients treated as outpatients, unclear if many of these patients had cryptococcal meningitis |
| Letsa 2018 | BMC Public Health. 2018 Jun 22;18(1):781 | Pneumococcal meningitis outbreak and associated factors in six districts of Brong Ahafo region, Ghana, 2016 | excluded as most suspected cases of pneumococcal meningitis during outbreak did not have microbiological confirmation (141/969 cases confirmed *Streptococcus* *pneumoniae*); median age young adult and don’t give breakdown of case-fatality by age; unclear completeness of outcomes records with low overall mortality (9% case-fatality rate) |
| Nonkala 2018 | Southern African Journal of Infectious Diseases (2018) 33:1 (8-11) | Diagnostic and mortality outcomes in a cohort of adult meningitis suspects in KwaZulu-Natal | included cases of cryptococcal and TB meningitis but did not provide outcomes differentiated by meningitis type; also appears most patients treated for TB meningitis based on lymphocytic pleocytosis without diagnostic details |
| Siika 2008 | East African medical journal Nov 2008;85(11):523-8 | Admission characteristics, diagnoses and outcomes of HIV-infected patients registered in an ambulatory HIV-care programme in western Kenya | included for cryptococcal meningitis; excluded for TB meningitis; 27 cases of TB meningitis, but did not include any diagnostic details or criteria |
| Thinyane 2015 | J Trop Med. 2015;2015:423161 | Clinical Presentation, Aetiology, and Outcomes of Meningitis in a Setting of High HIV and TB Prevalence | none of the cases of TB meningitis microbiologically diagnosed; details on imaging and other characteristics lacking; diagnostic criteria included clinical features of meningitis with negative CSF gram stain and culture and negative CSF cryptococcal antigen along with at least one of following: CSF pleocytosis with lymphocytic predominance, low glucose and elevated protein, evidence of active TB at another site, or brain CT findings suggestive of TB meningitis such as basal meningeal enhancement or hydrocephalus; proportion of suspected cases meeting each of these criteria not specified |

**No treatment provided or non-conventional treatment (11 articles) [plus one excluded from reference list]**

| First author and year | Journal details | Title | Reason |
| --- | --- | --- | --- |
| Heyderman 1998 | Clinical infectious diseases : an official publication of the Infectious Diseases Society of America Feb 1998;26(2):284-9 | Cryptococcal meningitis in human immunodeficiency virus-infected patients in Harare, Zimbabwe | cohort study of cryptococcal meningitis cases in which antifungal therapy was not available for patients |
| Joly 1997 | Clinical infectious diseases : an official publication of the Infectious Diseases Society of America Sep 1996;23(3):556-62 | Randomized comparison of amphotericin B deoxycholate dissolved in dextrose or Intralipid for the treatment of AIDS-associated cryptococcal meningitis | several reasons for exclusion: 1) study published mid-1990s that enrolled patients over 24 months but do not give dates of enrolment; 2) atypical dosing schedule for amphotericin of 2 weeks daily dosing then every other day for additional 4 weeks |
| Katende 2019 | Mycoses. 2019 Aug 28. doi: 10.1111/myc.12995. [Epub ahead of print] | Short course amphotericin B in addition to sertraline and fluconazole for treatment of HIV-associated cryptococcal meningitis in rural Tanzania | small clinical trial in Tanzania that compared treatment of fluconazole with sertraline or fluconazole and amphotericin B with sertraline for HIV-associated cryptococcal meningitis; excluding as sertraline is not standard treatment for cryptococcal meningitis and included in both regimens |
| Mayanja-Kizza 1998 | Clinical infectious diseases : an official publication of the Infectious Diseases Society of America Jun 1998;26(6):1362-6 | Combination therapy with fluconazole and flucytosine for cryptococcal meningitis in Ugandan patients with AIDS | cryptococcal meningitis RCT comparing low-dose fluconazole (200 mg/day) versus low-dose fluconazole (200 mg/day) with flucytosine (150 mg/kg/day) in Uganda; exclude as low-dose fluconazole used in this study not recommended in guidelines, also atypical dosing and frequency of flucytosine |
| Mwaba 2001 | Postgraduate medical journal Dec 2001;77(914):769-73 | Clinical presentation, natural history, and cumulative death rates of 230 adults with primary cryptococcal meningitis in Zambian AIDS patients treated under local conditions | pre-ART era study of cryptococcal meningitis in Zambia; for half of study period patients did not receive any antifungal therapy because of lack of hospital stock; large proportion of patients not offered any treatment |
| Maher 1994 | The Journal of infection Jan 1994;28(1):59-64 | Cryptococcal meningitis in Lilongwe and Blantyre, Malawi | study of cryptococcal meningitis in Malawi in which antifungal therapy not provided; most patients could not afford antifungal therapy and died following diagnosis |
| Orem 2005 | Tropical doctor Jan 2005;35(1):19-21 | Feasibility study of serial lumbar puncture and acetazolamide combination in the management of elevated cerebrospinal fluid pressure in AIDS patients with cryptococcal meningitis in Uganda | small cryptococcal meningitis clinical trial with or without adjunctive acetazolamide; acetazolamide associated with increased mortality and not used in clinical practice; <15 patients in control group that did not receive acetazolamide |
| Ouattara 2007 | Journal de Mycologie Medicale 2007;17(2):82-86 | Retrospective study of bacterial and cryptococcal meningitis occurring in HIV adult patients in Abidjan (Ivory Coast) | cross-sectional audit in Ivory Coast from 2001-2003 with cryptococcal meningitis cases; excluded for non-traditional dosing regimen of amphotericin B of 1 mg/kg every other day x8-10 weeks; similar study at same hospital obtained from references (Eholie Bull Soc Path Exotique 1997) also excluded for amphotericin B dosing every other day x6-8 weeks |
| Rhein 2016 | The Lancet. Infectious diseases Jul 2016;16(7):809-818 | Efficacy of adjunctive sertraline for the treatment of HIV-associated cryptococcal meningitis: an open-label dose-ranging study | phase II dose-finding study of adjunctive sertraline with amphotericin and fluconazole for treatment of cryptococcal meningitis with outcomes compared to historic control (Boulware 2014); exclude as all patients in study treated with sertraline, which is not used in clinical practice |
| Schaars 2006 | BMC Infectious Diseases 2006; 6:118 | Outcome of AIDS-associated cryptococcal meningitis initially treated with 200 mg/day or 400 mg/day of fluconazole | observational cryptococcal meningitis trial in South Africa; excluded as very low fluconazole dose (200-400 mg/day) used which is not used/recommended |
| Seboxa 2010 | Ethiopian medical journal Jul 2010;48(3):237-41 | Cryptococcal meningitis in patients with acquired immunodeficiency syndrome in prehaart era at Gondar College of Medical Sciences Hospital north-west Ethiopia | small retrospective Ethiopian study of patients with cryptococcal meningitis 1994-1997 at referral hospital; now non-traditional treatment with AmB at 0.1 mg/kg as test dose escalated over 48-72 hours, excluded for this reason; note that 31/31 patients died in this study, 17 of whom died before they received any treatment |
| Wang 2001 | Tropical doctor Oct 2001;31(4):221-2 | A clinical manifestation of AIDS with cryptococcal meningitis in Equatorial Guinea | patients with cryptococcal meningitis treated with acetazolamide, which is associated with increased mortality and not recommended in clinical practice |

**Large proportion of cases with missing mortality data or unclear follow-up (11 articles) [also listing additional study from reference review, Yassibanda et al.]**

| First author and year | Journal details | Title | Note |
| --- | --- | --- | --- |
| Aku 2017 | MMWR. Morbidity and mortality weekly report Aug 2017;66(30):806-810 | Meningitis Outbreak Caused by Vaccine-Preventable Bacterial Pathogens - Northern Ghana, 2016 | surveillance study of pneumococcal and meningococcal meningitis in Ghana; missing outcomes data on 25% (11/44) of patients with PCR-confirmed pneumococcal meningitis |
| Bahr 2015 | The international journal of tuberculosis and lung disease : the official journal of the International Union against Tuberculosis and Lung Disease Oct 2015;19(10):1209-15 | Improved diagnostic sensitivity for tuberculous meningitis with Xpert(®) MTB/RIF of centrifuged CSF | Small validation study for XPert MTB/RIF on CSF samples; missing outcomes data for 17% (3/18) of patients |
| Bozio 2018 | PLoS One. 2018 Sep 7;13(9):e0203205. doi: 10.1371/journal.pone.0203205 | Continued occurrence of serotype 1 pneumococcal meningitis in two regions located in the meningitis belt in Ghana five years after introduction of 13-valent pneumococcal conjugate vaccine | surveillance study of pneumococcal meningitis in Ghana; missing outcomes on high proportion of patients (36% [55/153]) |
| Cresswell 2018 | Wellcome Open Res. 2018 May 29;3:64. doi: 10.12688/wellcomeopenres.14610.2. eCollection 2018. | Can improved diagnostics reduce mortality from Tuberculous meningitis? Findings from a 6.5-year cohort in Uganda | a 6.5-year cohort of TB meningitis - including definite and probable cases - in Uganda; some overlap with included diagnostic accuracy studies from Uganda; large proportion of patients missing mortality outcomes or discharged against medical advice (27% [53/195]) |
| Eholie 2000 | Bulletin de la Societe de pathologie exotique (1990) Feb 2000;93(1):50-4 | [Adult non-viral lymphocytic meningitis in Abidjan (Côte d'Ivoire)] | included 70 cases of cryptococcal meningitis diagnosed at referral hospital in Ivory Coast from 1997-1999; the reported mortality was 59% but there are inadequate details provided on the period of follow-up in this study (if restricted to in-hospital or for longer duration of time); note that additional 13% of patients with diagnosed cryptococcal meningitis were lost to follow-up over unknown period; also included 45 cases of suspected TB meningitis (only 4 microbiologically-confirmed with mortality of 51% and 13% lost to follow-up over unknown time period; note that an Eholie et al. Bull Soc Path Ex 1997 article was found covering cryptococcal meningitis cases at same centre in 1995 which was also excluded for a number of reasons - non-standard treatment of amphotericin B every other day for unclear duration of time, unclear length of patient follow-up and also noted high loss to follow-up of 14% (42/64 still known to have died during follow-up |
| Kanyama 2019 | Clin Infect Dis. 2019 Jun 1. pii: ciz454. doi: 10.1093/cid/ciz454. [Epub ahead of print] | One year mortality outcomes from the ACTA trial of cryptococcal meningitis treatment in Malawi | Subset of patients from ACTA trial in Malawi only, not including all sites; mention as comment in discussion although exclude from meta-analysis |
| Kambiré 2016 | PloS one 2016;11(11):e0166384 | Nationwide Trends in Bacterial Meningitis before the Introduction of 13-Valent Pneumococcal Conjugate Vaccine-Burkina Faso, 2011-2013 | study of pneumococcal meningitis cases using national surveillance data in Burkina Faso; found low case-fatality from pneumococcal meningitis; however likely had significant under-ascertainment of outcomes through passive surveillance (comment on likely under-ascertainment of mortality in manuscript discussion) |
| Okome-Nkoumou 1999 | Bulletin de la Societe de pathologie exotique (1990) Dec 1999;92(5):288-91 | Bacterial meningitis in the adult. Study of 85 cases observed in the infectious disease unit of the Fondation Jeanne Ebori (F.J.E.), Libreville, Gabon | retrospective review of pneumococcal meningitis cases at referral hospital in Gabon; provided inpatient mortality of 10/45 (22%) excluding 10 additional patients (10/55 total) without outcomes |
| Park 2011 | International journal of STD & AIDS Apr 2011;22(4):199-203 | Long-term follow-up and survival of antiretroviral-naive patients with cryptococcal meningitis in the pre-antiretroviral therapy era, Gauteng Province, South Africa | reported long-term outcomes data from GERMS-SA cohort (McCarthy 2006) but had outcomes data on minority of patients post-discharge from hospital; of 721 persons discharged from hospital, 465 (64%) lost to follow-up |
| Patel 2010 | American journal of respiratory and critical care medicine Aug 2010;182(4):569-77 | Cerebrospinal T-cell responses aid in the diagnosis of tuberculous meningitis in a human immunodeficiency virus- and tuberculosis-endemic population | 38 cases of definite TBM (microbiological confirmation) but duration of follow-up / details on loss to follow-up not specified in text or supplemental material; supplemental material table E4 suggests significant number of patients with definite TB meningitis may have been to follow-up |
| Woldeamanuel 2001 | Ethiopian medical journal Jul 2001;39(3):185-92 | Cryptococcosis in patients from Tikur Anbessa Hospital, Addis Ababa, Ethiopia | small study in of 20 patients with cryptococcal meningitis; no outcomes data for 20%; noted high mortality 75% (12/16) in those with outcomes; notably high proportion of patients received no antifungal therapy |
| Yassibanda 2002 | Médecine d’Afrique Noire 2002;49(6):299-303 | Les infections neuromeningees de l’adulte en milieu hospitalier a Bangui: Aspects étiologiques, cliniques et évolutifs | study from reference list originally included for observational cryptococcal meningitis studies: however, excluded as does not specify length of follow-up in a prospective cohort (in discussion, mentions medium- to long-term outcomes but no time specified); also high overall loss to follow-up overall at 11.8% |

**Paediatric study (10 articles)**

| First author and year | Journal details | Title | Comments |
| --- | --- | --- | --- |
| Bernardino 2003 | Lancet (London, England) May 2003;361(9368):1564-5 | Bacterial meningitis in Angola. |  |
| Coldiron 2018 | Emerg Infect Dis. 2018 Sep;24(9):1720-1722 | Outbreak of Pneumococcal Meningitis, Paoua Subprefecture, Central African Republic, 2016-2017 |  |
| Crellen 2019 | Version 2. Wellcome Open Res. 2019 Mar 29 [revised 2019 Jan 1];3:134. doi:10.12688/wellcomeopenres.14868.2. eCollection 2018. | Seasonal upsurge of pneumococcal meningitis in the Central African Republic. | surveillance study including pneumococcal meningitis cases confirmed by latex agglutination; majority of patients were children with results not disaggregated for adults (median age of those with microbiologically-confirmed meningitis 11 years) |
| du Plessis 2008 | International journal of antimicrobial agents Nov 2008;32 Suppl 1():S66-70 | Serotype 6C is associated with penicillin-susceptible meningeal infections in human immunodeficiency virus (HIV)-infected adults among invasive pneumococcal isolates previously identified as serotype 6A in South Africa |  |
| Holliman 2007 | Transactions of the Royal Society of Tropical Medicine and Hygiene Apr 2007;101(4):405-13 | Epidemiology of invasive pneumococcal disease in Kumasi, Ghana |  |
| Kambire 2018 | J Infect. 2018 Mar; 76(3): 270–279 | Early impact of 13-valent pneumococcal conjugate vaccine on pneumococcal meningitis—Burkina Faso, 2014–2015 | majority of pneumococcal meningitis cases in children; mortality not differentiated for children vs. adults in this surveillance study from Burkina Faso |
| Klugman 1997 | South African medical journal 1997;87(7):912 | Impact of antimicrobial resistance and antibiotic choice on the outcome of pneumococcal meningitis |  |
| Moisi 2017 | PloS one 2017;12(1):e0170412 | Burden of Pneumococcal Disease in Northern Togo before the Introduction of Pneumococcal Conjugate Vaccine |  |
| Mueller 2012 | PLoS One. 2012;7(12):e52464. doi: 10.1371/journal.pone.0052464 | Pneumococci in the African Meningitis Belt: Meningitis Incidence and Carriage Prevalence in Children and Adults | surveillance study of pneumococcal infection in Burkina Faso with 114 cases of confirmed pneumococcal meningitis 2007-2009 at surveillance sites; excluded as majority of patients children (<15 years) with only 42% (48/114) ≥15 years of age; note overall mortality for pneumococcal meningitis 40% |
| Wiersinga 2004 | Annals of tropical medicine and parasitology Apr 2004;98(3):271-8 | High mortality among patients with bacterial meningitis in a rural hospital in Tanzania |  |

**Period of observation <1990 (7 articles)**

| First author and year | Journal details | Title |
| --- | --- | --- |
| Bissagnene 1994 | Medecine et Maladies Infectieuses 1994;24(SPEC. ISSUE MAY):580-585 | Current aspects of neuromeningitic cryptococcus in Abidjan |
| Bogaerts 1999 | The Journal of infection Jul 1999;39(1):32-7 | AIDS-associated cryptococcal meningitis in Rwanda (1983-1992): epidemiologic and diagnostic features |
| Bouzouaia 1994 | Semaine des Hopitaux 1994;70(7-8):205-210 | Pneumococcal infections in Tunisia. A review of seventy-five cases |
| Campagne 1999 | Bulletin of the World Health Organization 1999;77(6):499-508 | Epidemiology of bacterial meningitis in Niamey, Niger, 1981-96 |
| Laroche 1992 | Journal of medical and veterinary mycology : bi-monthly publication of the International Society for Human and Animal Mycology 1992;30(1):71-8 | Cryptococcal meningitis associated with acquired immunodeficiency syndrome (AIDS) in African patients: treatment with fluconazole |
| Pallangyo 1992 | AIDS (London, England) Sep 1992;6(9):971-6 | High HIV seroprevalence and increased HIV-associated mortality among hospitalized patients with deep bacterial infections in Dar es Salaam, Tanzania |
| Pécoul 1991 | Lancet (London, England) Oct 1991;338(8771):862-6 | Long-acting chloramphenicol versus intravenous ampicillin for treatment of bacterial meningitis |

**Review article (7 articles)**

| First author and year | Journal details | Title |
| --- | --- | --- |
| Alkali 2013 | Annals of African medicine ;12(1):1-10 | NeuroAIDS in sub-Saharan Africa: a clinical review |
| Gessner 2009 | BMC Infectious Diseases 2009;10: | African meningitis belt pneumococcal disease epidemiology indicates a need for an effective serotype 1 containing vaccine, including for older children and adults |
| Jullien 2016 | Cochrane Database of Systematic Reviews 2016;2016(9): | Six months therapy for tuberculous meningitis |
| Pasquier 2018 | Clin Infect Dis. 2018 Mar 19;66(7):1122-1132. doi: 10.1093/cid/cix870 | Long-term Mortality and Disability in Cryptococcal Meningitis: A Systematic Literature Review |
| Veltman 2014 | Journal of the International AIDS Society 2014;17():19184 | Meningitis in HIV-positive patients in sub-Saharan Africa: a review |
| Woldeamanuel 2014 | Journal of neurology May 2014;261(5):851-65 | A 43-year systematic review and meta-analysis: case-fatality and risk of death among adults with tuberculous meningitis in Africa |
| [no authors listed] | Releve epidemiologique hebdomadaire Jun 2016;91(23):298-302 | Pneumococcal meningitis outbreaks in sub-Saharan Africa |

**Case series or research letter (5 studies)**

| First author and year | Journal details | Title | Reason |
| --- | --- | --- | --- |
| Bourée 2014 | Medecine et sante tropicales ;24(2):146-7 | [Decline of cryptococcal meningitis] | research news summary of another published study |
| Marais 2018 | Clinical infectious diseases : an official publication of the Infectious Diseases Society of America Jan 2018;(): | Spinal tuberculosis: Clinicoradiological findings in 274 patients | case series of spinal TB with or without other manifestations |
| Nkoumou 2003 | Journal of Acquired Immune Deficiency Syndromes 2003;32(3):345-346 | Bacterial and mycobacterial meningitis in HIV-positive compared with HIV-negative patients in an internal medicine ward in Libreville, Gabon | letter to the editor; also TB meningitis defined as lymphocytic meningitis without identified cause which likely included number of non-TB meningitis cases |
| Patel 2004 | Clinical infectious diseases : an official publication of the Infectious Diseases Society of America Mar 2004;38(6):851-6 | Multidrug-resistant tuberculous meningitis in KwaZulu-Natal, South Africa | case series of multi-drug resistant TB meningitis cases; not representative of TB meningitis cases as restricted to drug-resistant cases |
| Pepper 2009 | Clinical infectious diseases : an official publication of the Infectious Diseases Society of America Jun 2009;48(11):e96-107 | Neurologic manifestations of paradoxical tuberculosis-associated immune reconstitution inflammatory syndrome: a case series | case series of patients with TB-IRIS |

**Not in sub-Saharan Africa (2 articles)**

| First author and year | Journal details | Title | Notes |
| --- | --- | --- | --- |
| Alvarez-Uria 2015 | Journal of tropical medicine 2015;2015():864271 | Short-Course Induction Treatment with Intrathecal Amphotericin B Lipid Emulsion for HIV Infected Patients with Cryptococcal Meningitis |  |
| Zurcher 2019 | J Int AIDS Soc. 2019 Sep; 22(9): e25392. | Diagnosis and clinical outcomes of extrapulmonary tuberculosis in antiretroviral therapy programmes in low- and middle-income countries: a multicohort study | Included 22 countries in several regions; provided mortality outcomes for 43 TB meningitis overall but not restricted to countries from sub-Saharan Africa |
